# Asymmetric Counteranion-Directed Halogen Bonding Catalysis

**DOI:** 10.1021/jacs.4c18378

**Published:** 2025-03-03

**Authors:** Dominik L. Reinhard, Anna Iniutina, Sven Reese, Tushar Shaw, Christian Merten, Benjamin List, Stefan M. Huber

**Affiliations:** † Fakultät für Chemie und Biochemie, Organische Chemie II, Ruhr-Universität Bochum, 44801 Bochum, Germany; ‡ Max-Planck-Institut für Kohlenforschung, 45470 Mülheim an der Ruhr, Germany

## Abstract

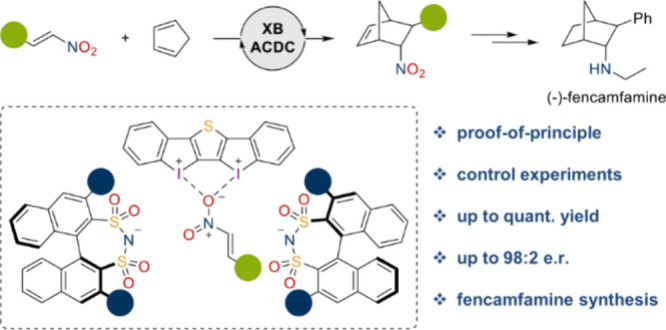

Halogen bonding has been established as a promising tool in organocatalysis. Asymmetric processes are nevertheless scarce, and their applications are limited to a few studies applying chiral halogen bond donors. Herein, we combine halogen bonding with asymmetric counteranion-directed catalysis, providing the first highly enantioselective example of such an approach. A strong bidentate iodine(III)-based catalyst with chiral disulfonimides as counteranions is applied in the first asymmetric organocatalysis of the Diels–Alder reaction between cyclopentadiene and *trans*-β-nitrostyrene, the key step in the synthesis of the drug fencamfamine, which was prepared with high enantioselectivity.

The use of noncovalent interactions such as hydrogen bonding in asymmetric organocatalysis has been established since the late 1990s.^1−3^ Halogen bonding (XB) describes a topologically related interaction between a Lewis acidic halogen atom in a molecule (the XB donor) and a Lewis base (XB acceptor).^4^ It has gained, besides its use in fields like crystal engineering^5,6^ and molecular recognition,^7^ increased interest in organocatalysis.^8−10^ In the past few years, several reports were published on asymmetric catalysis employing bi- or multifunctional catalysts including XB donor sites.^11−14^ For example, Yoshida et al. recently showed remarkable enantioselectivities with chiral halonium(III)-based XB catalysts that featured additional hydrogen bonding sites.^12,13^ However, examples with XB as the decisive mode of activation are scarce: The proof-of-principle case was reported by Huber et al. in 2020, reaching 33% enantiomeric excess (e.e.);^15^ García Mancheño et al. published two studies reaching up to 90% e.e. but for substrates involving an additional XB donor site.^16,17^ In 2024, high enantioselectivites of 98% e.e. were achieved with XB as the key interaction, both by Huber et al.^18^ and Nachtsheim et al.^19^ In all of these cases, enantioinduction was achieved with a chiral halogen bond donor.

Another approach for asymmetric catalysis would be to activate the reaction by an *achiral* cationic XB donor in the presence of a chiral counteranion, following the general concept of Asymmetric Counteranion-Directed Catalysis (ACDC),^20^ which has been applied in several areas such as covalent organocatalysis,^21^ transition-metal catalysis,^22^ and photoredox catalysis.^23^ In 2015, Han and Liu et al. reported the catalysis of a Mannich reaction with diaryliodonium(III) salts, but the use of a chiral phosphate counteranion yielded only a small e.e. of 7%.^24−26^ In 2019, two groups used monodentate cationic XB donors with chiral phosphate counteranions in asymmetric catalysis (Figure 1). Scheidt et al. reported the catalysis of a conjugate addition reaction by a iodotriazolium salt with 22% e.e. (corresponding acid: 8% e.e.), but control experiments suggested hidden Brønsted acid catalysis to be the true mode of action.^27^ Yeung et al. used a iodoimidazolium salt to promote an addition reaction with 44% e.e. (corresponding acid: 31%).^28^ However, additional control experiments to exclude hidden Brønsted acid catalysis were not provided. Thus, there is currently no conclusive proof-of-principle for Asymmetric Counteranion-Directed Halogen Bonding Catalysis (XB-ACDC).

**Figure 1:**
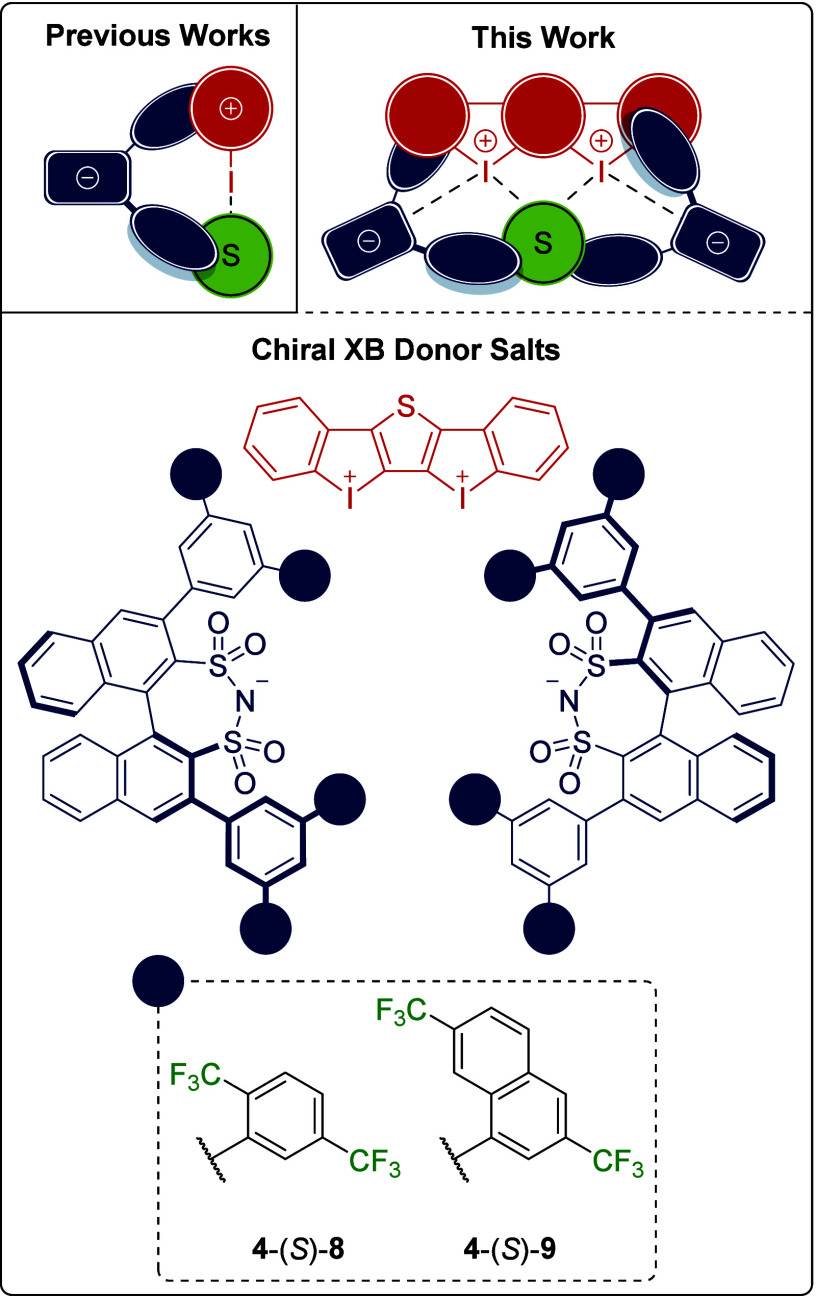
Concepts of previous approaches to XB-ACDC^27,28^ versus the concept of this work (red: XB catalyst, blue: chiral counteranion, green: substrate) and our catalyst system.

Herein, we aimed to find a clear case for this concept with high enantioselectivity. Arguably, the main obstacle in demonstrating the feasibility of XB-ACDC is the exclusion of hidden Brønsted acid catalysis. Therefore, we were interested in challenging reactions that cannot be readily catalyzed by (chiral) acids. We thus report the asymmetric catalysis of the Diels–Alder reaction between cyclopentadiene (**2**) and *trans*-β-nitrostyrene (**1a**) (Scheme 1) with salts consisting of a bidentate XB donor (**XBD**) and chiral disulfonimides (**DSI**s) (Figure 1).

**Scheme 1:**
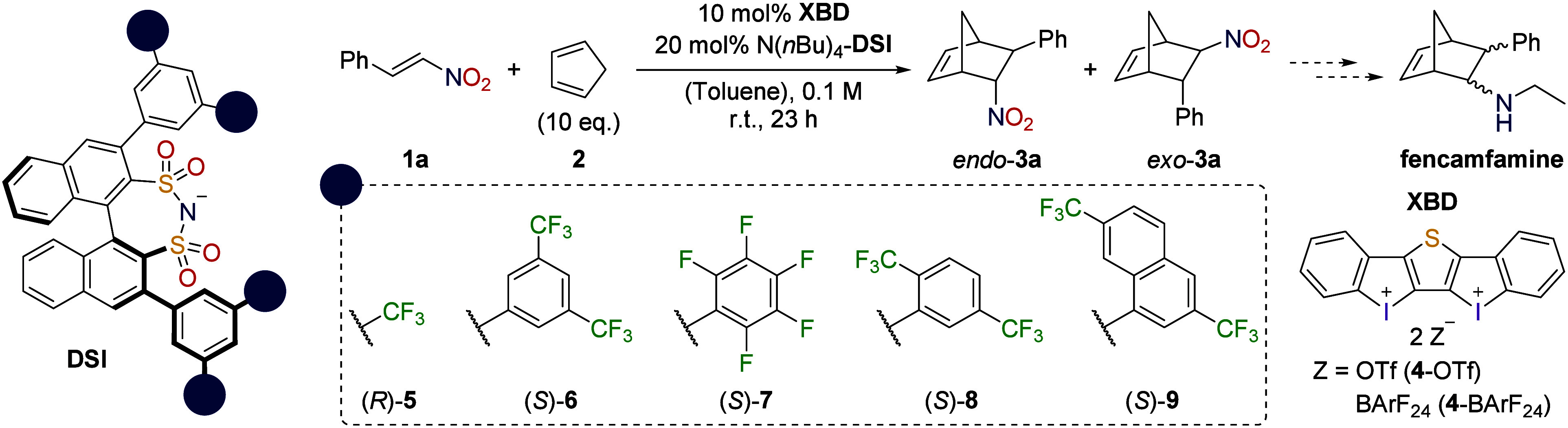
Catalysis of the Diels–Alder Reaction between *trans*-β-Nitrostyrene (**1a**) and Cyclopentadiene (**2**) Using an Achiral XB Donor Salt (XBD) with Chiral DSI Additives For clarity, *endo*- and *exo*-**3a** are depicted as defined enantiomers.

This cycloaddition reaction was first reported in 1939^29,30^ and is the key step in the synthesis of the drug fencamfamine.^31,32^ Only one example of efficient asymmetric catalysis of this cycloaddition has been reported by Carmona and Ferrer et al., who employed a chiral iridium-catalyst (up to 90% e.e.).^33^ While the reaction can also be activated via hydrogen bonding,^34^ no asymmetric organocatalysis has been reported.^35^ Recently, one of our groups developed several applications of a bidentate iodine(III)-based XB catalyst, bis(iodolium) **4**-BArF_24_ (Scheme 1), among them the activation of *trans*-β-nitrostyrene (**1a**).^36^ Therefore, we hypothesized that it may also be suited to catalyze the above-mentioned Diels–Alder reaction, potentially enantioselectively, using an ACDC process. The corresponding transition state with catalyst **4** was obtained via DFT calculations (M06-2X-D3,^37,38^ def2-TZVP(D)^39−41^) and showed bidentate XB in the center toward one nitro oxygen (Figure 2).^42^ This catalyst has two additional monodentate binding sites at the iodines, which may bind the anions, forming a chiral pocket (Figure 1).

**Figure 2:**
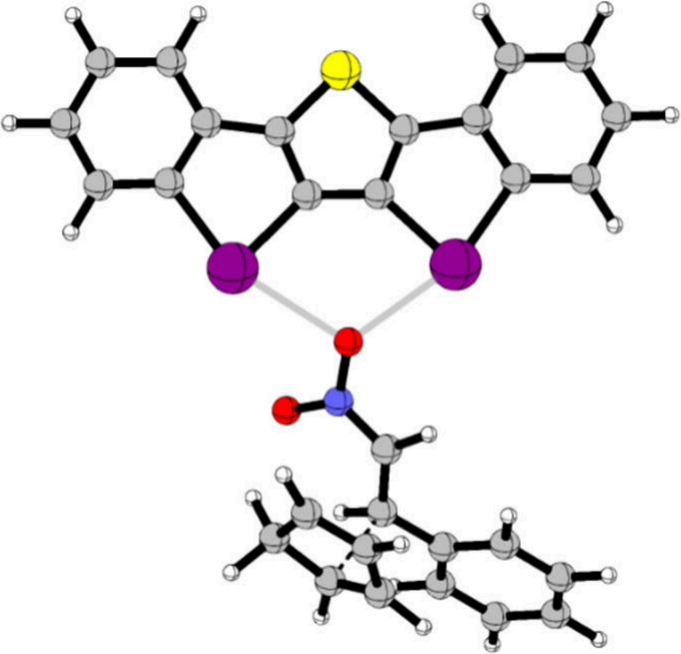
Transition state of the Diels–Alder reaction (Scheme 1) catalyzed by XB donor **4**, calculated using DFT (M06-2X-D3,^37,38^ def2-TZVP(D)^39−41^). Graphic via CYLview20.^43^

Initially, we used an additive approach: XB donor **4**-BArF_24_ was applied as salt with the weakly coordinating anion tetrakis[3,5-bis(trifluoromethyl)phenyl]borate (^−^BArF_24_) at 10 mol % catalyst loading combined with 20 mol % DSI salt N(*n*Bu)_4_-(*R*)-**5** in toluene at room temperature (Scheme 1). We hypothesized an *in situ* anion metathesis to the bis(iodolium)-DSI salt, accompanied by the formation of innocent N(*n*Bu)_4_-BArF_24_. The mixture was prestirred for 30 min before the addition of cyclopentadiene to ensure the formation of the chiral salt. After 23 h, a yield of 37% of *endo*-**3a** was determined via ^1^H NMR spectroscopy (Table 1, entry 1).^44^ A significant enantioselectivity for *endo*-**3a** was observed (75:25 e.r.).

**Table 1:** Reaction Optimization and Control Experiments

entry^a^	cat. (mol %)	add. (mol %)	yield^b^ (%)	e.r.^c^
1	**4**-BArF_24_ (10)	N(*n*Bu)_4_-(*R*)-**5** (20)	37	75:25
2	**4**-BArF_24_ (10)	-	60	-
3	**4**-OTf (10)	-	19	-
4	**4**-OTf (10)	N(*n*Bu)_4_-(*R*)-**5** (20)	19	50:50
5	-	N(*n*Bu)_4_-(*R*)-**5** (20)	8	51:49
6	-	H-(*R*)-**5** (20)	12	51:49
7	-	-	12	-
8	**4**-BArF_24_ (10)	H-(*R*)-**5** (20)	<5	53:47
9	**4**-BArF_24_ (10)	N(*n*Bu)_4_-(*S*)-**6** (20)	23	69:31
10	**4**-BArF_24_ (10)	N(*n*Bu)_4_-(*S*)-**7** (20)	35	76:24
11	**4**-BArF_24_ (10)	N(*n*Bu)_4_-(*S*)-**8** (20)	46	86:14
12	**4**-BArF_24_ (10)	N(*n*Bu)_4_-(*S*)-**9** (20)	69	85:15
13	**4**-BArF_24_ (10)	N(*n*Bu)_4_-(*S*)-**9** (10)	34	48:52
14	**4**-BArF_24_ (10)	N(*n*Bu)_4_-(*S*)-**9** (30)	54	82:18
15	**-**	Na-(*S*)-**9** (20)	25	49:51
16	**4**-(*S*)-**9** (10)	-	>95	88:12
17^d^	**4**-(*S*)-**9** (10)	-	>95	89:11
18^d^^,^^e^	**4**-(*S*)-**8** (10)	-	94^f^	93:7
19^g^^,^^h^	**4**-(*S*)-**8** (10)	-	56^f^	94.5:5.5
20^g^	**4**-(*S*)-**8** (5)	-	32	93.5:6.5
21^g^	**4**-(*S*)-**8** (2)	-	10	87:13
22^d^	**-**	H-(*S*)-**8** (20)	9	51:49
23^d^	**-**	-	9	-

a Reactions (Scheme 1) performed at 12.5/25 μmol scale using 10 equiv of cyclopentadiene (**2**) and the catalyst (cat.)/additive (add.) in the given amounts in toluene (100 mM).

b Determined for *endo*-**3a**^44^ via ^1^H NMR spectroscopy using methyl 3,5-dinitrobenzoate as internal standard.

c Determined for *endo*-**3a** via chiral HPLC.

d 50 mM concentration.

e 50 μmol scale.

f Isolated yield of **3a** (endo:exo >100:1).

g 12.5 mM concentration.

h 50 and 100 μmol scale.

Without DSI additive, a yield of 60% was achieved (Table 1, entry 2). The *in situ* anion metathesis required the weakly coordinating ^–^BArF_24_ anion, as experiments employing the triflate salt **4**-OTf were unsuccessful (Table 1, entries 3 and 4).^45^ While other mono- and bidentate iodine(III)-based XB donors^46−48^ were also able to catalyze the reaction as ^–^BArF_24_-salts, none of them gave any enantioselectivity when used in combination with DSI-additives (see the SI).

Next, control experiments were conducted to exclude hidden Brønsted acid catalysis. With the pure salt additive (20 mol %, Table 1, entry 5) or the corresponding acid (20 mol %, Table 1, entry 6), yields comparable to the background reactivity (12% after 23 h, Table 1, entry 7) were achieved without any enantioselectivity. The combination of 10 mol % bis(iodolium) **4**-BArF_24_ and 20 mol % of the acid H-(*R*)-**5** (Table 1, entry 8) also provided only trace amounts of product (<5% yield) but with full consumption of the diene.^49^ Possibly, the acid is activated via coordination of the DSI-anion by the XB donor, leading to side reactions. In the product obtained, a minimal enantioselectivity (53:47 er) was determined. All of these results clearly contradict hidden Brønsted acid catalysis.

With the working XB-ACDC method in hand, we screened different DSI additives. The exchange of the −CF_3_ groups on prototypic DSI (*R*)-**5** by fluorinated arenes gave promising results (see the SI). While the use of anions (*S*)-**6** and (*S*)-**7** gave lower or similar enantioselectivity (Table 1, entries 9 and 10), with (*S*)-**8** and (*S*)-**9** better yields and already good enantioselectivity (Table 1, entries 11 and 12, up to 86:14 e.r.) were achieved. When employing only 10 mol % of additive N(*n*Bu)_4_-(*S*)-**9**, the enantioselectivity was shut down (Table 1, entry 13). This indicates that two counteranions are needed for this process and could be considered as a first hint that a supramolecular catalyst complex as shown in Figure 1 may be formed. When increasing the amount of additive to 30 mol %, the results were also worse than with 20 mol % (Table 1, entry 14). Presumably, the additional ion pairs (N(*n*Bu)_4_-BArF_24_ or -DSI) hinder the reaction to some extent, as the yield also decreased. In order to further improve the enantioinduction, we chose to move away from the additive approach and synthesized defined complexes **4**-(*S*)-**8** and **4**-(*S*)-**9** (Figure 1) via anion metathesis using the sodium salt of the corresponding DSI (Na-(*S*)-**8** and Na-(*S*)-**9**) and the bis(iodolium) triflate **4**-OTf under microwave irradiation (see the Supporting Information).

With these compounds in hand, the reaction conditions were optimized starting with complex **4**-(*S*)-**9**.^50^ At the initial conditions of 10 mol % catalyst loading, quantitative conversion and a slightly improved enantioselectivity (Table 1, entry 16, 88:12 e.r.) were found. The corresponding sodium salt Na-(*S*)-**9** used for the anion metathesis was also able to (slightly) activate the reaction, presumably via cation−π interactions, but not in an enantioselective fashion (Table 1, entry 15). When reducing the concentration of nitrostyrene **1a** from 100 to 50 mM, a minimal increase in enantioselectivity to 89:11 e.r. was observed (Table 1, entry 17). With the other catalyst **4**-(*S*)-**8**, a significantly better result was achieved: at 10 mol % catalyst loading and 50 mM substrate concentration, 94% isolated yield and 93:7 e.r. were obtained (Table 1, entry 18). By further dilution to 12.5 mM using 10 mol % **4**-(*S*)-**8**, the result was finally optimized to 94.5:5.5 e.r. (Table 1, entry 19) at a lower isolated yield of 56% after 23 h. Under both of these conditions, excellent diastereoselectivity was determined (*endo*:*exo* > 100:1). The absolute configuration of the major enantiomer was determined to be (1*R*,4*S*,5*R*,6*S*) by Vibrational Circular Dichroism (VCD) spectroscopy.^51^ A reduced catalyst loading of 5 mol % led to a minimal decrease in enantioselectivity (93.5:6.5 e.r.), while a markedly lower yield of 32% was determined (Table 1, entry 20). Further reduction to 2 mol % resulted in even less enantioselectivity (87:13 er) and a poor yield of 10% (Table 1, entry 21). As expected from the control experiments, the corresponding acid H-(*S*)-**8** (Table 1, entry 22) did not catalyze this reaction (same yield as without catalyst, Table 1, entry 23) and also did not induce any noticeable enantioselectivity.

After the reaction optimization, a substrate scope was investigated employing **4**-(*S*)-**8** at a 50 mM substrate concentration (Scheme 2). All products derived from nitrostyrenes formed with high diastereoselectivity (*endo*:*exo* > 100:1). While using methyl-substituted substrate **1b**, 95:5 e.r. was achieved at 91% yield of **3b** even at a reduced catalyst loading of 5 mol %. Halogenated nitrostyrenes **1c** and **1d** yielded very good enantioselectivity, similar to prototypical nitrostyrene **1a** (**3c**: 93:7 e.r., **3d**: 94.5:5.5 e.r. at 10 mol % catalyst loading), and yields of 75 and 98%, respectively. Methoxy-substituted nitrostyrenes worked especially well, also with 5 mol % catalyst: those with one (**3e**: 96.5:3.5 e.r., **3f**: 97:3 e.r.) or two (**3g**: 98:2 e.r., **3h**: 97.5:2.5 e.r.) of these substituents gave excellent results. Due to the electron-donating nature of these substituents, the nitrostyrenes are less reactive for the Diels–Alder reaction but exhibit a higher Lewis basicity. Therefore, these compounds should bind more strongly to the catalyst. When extending the scope to aliphatic nitroolefin **1i**, low activity and moderate diastereo- and enantioselectivity were observed (8% yield after 65 h, 9:1 d.r., 79:21 e.r.).^52^

**Scheme 2:**
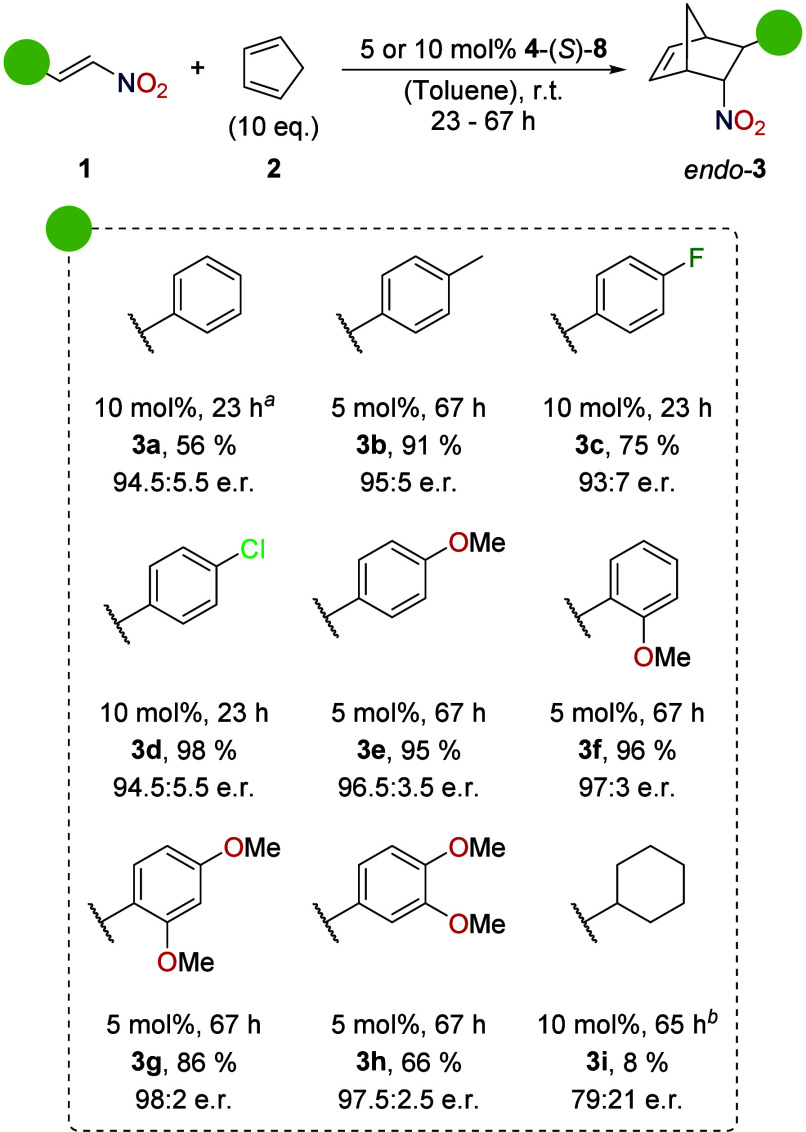
Substrate Scope (at 100 μmol Scale, 50 mM Concentration; e.r. Determined via Chiral HPLC; Products Isolated with >100:1 d.r.) Performed at 50 and 100 μmol scale, at 12.5 mM concentration. 9:1 d.r. The depicted absolute configuration (1*R*,4*S*,5*R*,6*S*) was proven for *endo*-**3a** by VCD spectroscopy.^51^

Finally, the XB-catalyzed reaction employing nitrostyrene **3a** was successfully scaled (0.76 mmol) with the same selectivity, isolating the product with 91% yield at an increased reaction time (54 h) with the e.r. of 94.5:5.5 (Scheme 3). In this case, the catalyst was recovered (73%) by precipitation containing minor impurities of dicyclopentadiene. Furthermore, this compound was then successfully transformed into highly enantiopure (−)-fencamfamine (*endo*-**12**, 94.5:5.5 e.r.) in 64% yield over three steps (Scheme 3). This is the first reported route toward this pharmaceutical.

**Scheme 3:**
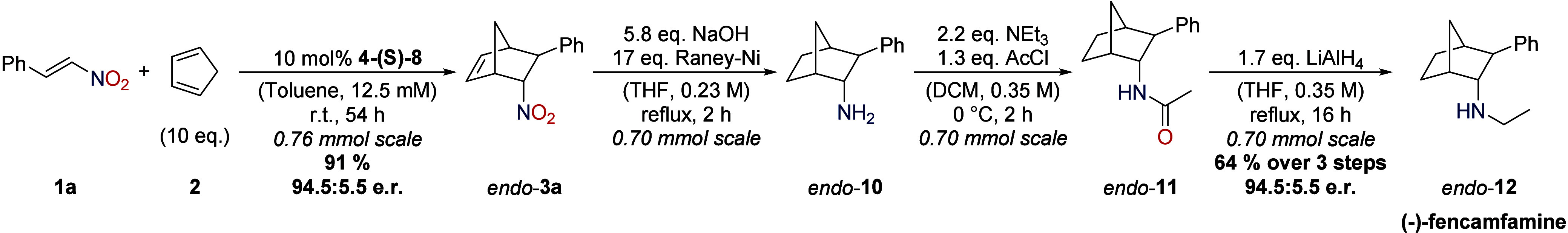
Scale-up and Isolation of Enantiopure (−)-Fencamfamine The depicted absolute configuration (1*R*,4*S*,5*R*,6*S*) was proven for *endo*-**3a** by VCD spectroscopy.^51^

In conclusion, the catalysis of the Diels–Alder cycloaddition of cyclopentadiene (**2**) and nitrostyrenes **1** by Asymmetric Counteranion-Directed Halogen Bonding Catalysis (XB-ACDC) with high enantioselectivities of up to 98:2 er was described (including the synthesis of enantiopure fencamfamine from the catalysis product). This represents the first report on asymmetric organocatalysis of this type of Diels–Alder reaction. By control experiments, hidden Brønsted acid catalysis was excluded, and therefore, the first conclusive and highly enantioselective example for XB-ACDC is provided herein. The importance of the 1:2 ratio between the cationic XB donor and chiral anions indicates that our initially devised supramolecular complex (Figure 1) could indeed be relevant. Further investigations on the mechanism and scope of this reaction are underway.

## Supporting Information Available

Additional experimental details for the synthesis of the catalysts, for the catalysis experiments and their evaluation as well as for the VCD experiments, computational data, and analytical data for all new compounds (PDF)
