# Supplementary material for: Asymmetric Counteranion-Directed Halogen Bonding Catalysis
Source: J Am Chem Soc. 2025 Mar 3;147(10):8107–12. doi: 10.1021/jacs.4c18378 (PMC11912313; doi:10.1021/jacs.4c18378)
Supplement: Supplementary file 1 — ja4c18378_si_001.pdf [file ja4c18378_si_001.pdf]

SUPPORTING INFORMATION

Asymmetric Counteranion-Directed Halogen Bonding Catalysis

Dominik L. Reinhard,[a,b] Anna Iniutina,[b] Sven Reese,[a] Tushar Shaw,[a] Christian Merten,[a]
Benjamin List,[b]* Stefan M. Huber[a]*

[a]Fakultät für Chemie und Biochemie, Ruhr-Universität Bochum,

44801 Bochum, Germany

stefan.m.huber@rub.de

[b]Max-Planck-Institut für Kohlenforschung

45470 Mülheim an der Ruhr, Germany

list@kofo.mpg.de

CONTENTS

Contents I
1. General Remarks 2
  1.1 Chemicals & Solvents 2
  1.2 Methods for Synthesis and Chromatography 2
  1.3 Analytical Methods 3
2. Catalyst synthesis 4
  2.1 Synthesis of known catalysts 4
  2.2 Synthesis of new catalysts 5
3. Screening 11
  3.1 General Preparation of $N(nBu)_4$-Salts 11
  3.2 Screening Procedure for Diels-Alder Reaction 11
  3.3 Additional Screening Results 12
4. Isolation and Scope 15
  4.1 Preparative Procedure for Diels-Alder Reaction 15
  4.2 (-)-(1*R*,4*S*,5*R*,6*S*)-*Endo*-5-nitro-6-phenylbicyclo[2.2.1]hept-2-ene (*endo*-3a) 15
  4.3 (-)-*Endo*-5-nitro-6-(*p*-tolyl)bicyclo[2.2.1]hept-2-ene (*endo*-3b) 18
  4.4 (-)-*Endo*-5-nitro-6-(4-fluorophenyl)bicyclo[2.2.1]hept-2-ene (*endo*-3c) 19
  4.5 (-)-*Endo*-5-nitro-6-(4-chlorophenyl)bicyclo[2.2.1]hept-2-ene (*endo*-3d) 20
  4.6 (-)-*Endo*-5-nitro-6-(4-methoxyphenyl)bicyclo[2.2.1]hept-2-ene (*endo*-3e) 21
  4.7 (-)-*Endo*-5-nitro-6-(2-methoxyphenyl)bicyclo[2.2.1]hept-2-ene (*endo*-3f) 22
  4.8 (-)-*Endo*-5-nitro-6-(2,4-dimethoxyphenyl)bicyclo[2.2.1]hept-2-ene (*endo*-3g) 23
  4.9 (-)-*Endo*-5-nitro-6-(3,4-dimethoxyphenyl)bicyclo[2.2.1]hept-2-ene (*endo*-3h) 24
  4.10 (-)-*Endo*-5-cyclohexyl-6-nitrobicyclo[2.2.1]hept-2-ene (*endo*-3i) 25
  4.11 Limitations 26
5. Synthesis of (-)-fencamfamine (*endo*-12) 28
  5.1 *Endo*-phenylbicyclo[2.2.1]heptan-2-amine (*endo*-10) 28
  5.2 *Endo*-phenylbicyclo[2.2.1]heptan-2-amine (*endo*-11) 28
  5.3 (-)-*Endo*-N-ethyl-3-phenylbicyclo[2.2.1]heptan-2-amine (12) 29
6. Absolute Configuration Determination by VCD spectroscopy 30
  6.1 Experimental details 30
  6.2 Computational details 30
  6.3 Analysis of the spectra 30
  6.4 Cartesian coordinates of c1 and c2 31
7. DFT Calculation of the Transition State 32
  7.1 Computational details 32
  7.2 Transition state of the Diels-Alder reaction between *trans*-β-nitrostyrene (1a) and cyclopentadiene (2) involving catalyst 4 (without counteranions) 32
8. References 33
9. NMR spectra 34
10. HPLC traces 53
  10.1 5-Nitro-6-phenylbicyclo[2.2.1]hept-2-ene (3a) 53
  10.2 5-Nitro-6-(*p*-tolyl)bicyclo[2.2.1]hept-2-ene (3b) 57
  10.3 5-Nitro-6-(4-fluorophenyl)bicyclo[2.2.1]hept-2-ene (3c) 59
  10.4 5-Nitro-6-(4-chlorophenyl)bicyclo[2.2.1]hept-2-ene (3d) 61
  10.5 5-Nitro-6-(4-methoxyphenyl)bicyclo[2.2.1]hept-2-ene (3e) 63
  10.6 5-Nitro-6-(2-methoxyphenyl)bicyclo[2.2.1]hept-2-ene (3f) 65
  10.7 5-Nitro-6-(2,4-dimethoxyphenyl)bicyclo[2.2.1]hept-2-ene (3g) 67
  10.8 5-Nitro-6-(3,4-dimethoxyphenyl)bicyclo[2.2.1]hept-2-ene (3h) 69
  10.9 2-(3-Nitrobicyclo[2.2.1]hept-5-en-2-yl)thiophene (SI-25) 71
  10.10 5-(2-chlorophenyl)-6-nitrobicyclo[2.2.1]hept-2-ene (SI-26) 73
  10.11 *N*-ethyl-3-phenylbicyclo[2.2.1]heptan-2-amine (12) 76
12. GC traces 78
  12.1 *Endo*-5-cyclohexyl-6-nitrobicyclo[2.2.1]hept-2-ene (*endo*-3i) 78

1. General Remarks

Most of the described experiments and collection of analytical data were done at the "*Ruhr-Universität Bochum*" (RUB). A part of the experiments was done at the "*Max-Planck-Institut für Kohlenforschung*" (MPI-KoFo) and the different circumstances will be clarified.

1.1 Chemicals & Solvents

All used fine chemicals and solvents were purchased from commercial sources and were used without further purification, if not stated otherwise. Technical grade *n*-pentane and diethyl ether were purified by distillation. Cyclopentadiene was prepared by thermal cracking of dicyclopentadiene similar as reported in literature[1] and then stored under argon in a Schlenk tube in dry-ice. Dry dichloromethane, diethyl ether, and tetrahydrofuran were received from an *MBRAUN MB SPS-800*. Other dry solvents were obtained by storage over flame-dried 3 Å or 4 Å molecular sieves under argon. The water content of solvents was determined with a *Karl Fischer Titroline®7500KF trace* from *SI Analytics* with *Honeywell (Fluka) Hydranal Coulomat-AD.*

For experiments done at the MPI-KoFo: (Dry) solvents were either purchased from commercial sources or obtained by the technical department of the MPI-KoFo. Dry solvents were distilled from appropriate drying agents.

1.2 Methods for Synthesis and Chromatography

All moisture or air-sensitive reactions were performed using standard Schlenk technique under argon as inert gas using dry solvents and flame- or oven-dried glassware, if necessary. Microwave-assisted synthesis was done in a *CEM Discover SP* microwave reactor. Analytical thin-layer chromatography (TLC) was performed using *Merck* (*Supelco®*) TLC aluminum sheets (silica gel 60, F254) and spots were visualized using a UV lamp at 254 nm or by staining with $KMnO_4$. For column chromatography silica gel of grain size 0.04-0.063 mm (*Machery-Nagel Si60*) was used. It was performed under atmospheric or increased pressure (by use of a hand- or peristaltic pump). Used eluents and retardation factors (R_f) are mentioned in the experiments. Preparative thin-layer chromatography (pTLC) was performed on *Merck* (*Supelco®*) TLC glass plates (silica gel 60, F254) and spots were visualized using a UV lamp at 254 nm.

For experiments done at the MPI-KoFo: Thin layer chromatography (TLC) was performed using silica gel precoated plastic sheets (Polygram SIL G/UV254, 0.2 mm, with fluorescent indicator; Macherey-Nagel) which was visualized with a UV lamp (254 nm) and/or Cerium Ammonium Molybdate (CAM) stain. Column chromatography was performed using Merck silica gel (60 Å, 230−400 mesh, particle size 0.040−0.063 mm) and technical grade solvents. Preparative TLC was performed on silica gel pre-coated glass plates by *Machery-Nagel* and spots were visualized using a UV lamp at 254 nm.

1.3 Analytical Methods

Nuclear Magnetic Resonance (NMR) Spectroscopy:

NMR spectra were measured at ca. 300 K. NMR data was obtained at the RUB using *Bruker DPX 250*, *AVIII 300*, *AVIII 400*, *DRX 400*, or *Neo 400* spectrometers. At the MPI-KoFo, the data was recorded on a Bruker *Avance III 500*. Chemical shifts (δ) are given in parts per million (ppm) and are internally referenced to tetramethylsilane by residual solvent signals.[2] Spectra were analyzed using *MestReNova*. Multiplicities are abbreviated as s (singlet), d (doublet), t (triplet), q (quartet), p (pentet), sept (septet), m (multiplet), and combinations of those, e.g. td (triplet of doublets). The relative integral and the coupling constant (*J* in Hz) are indicated if possible. NMR signals were assigned to the corresponding atoms with the best possible accuracy using COSY, HSQC, and HMBC spectra.

Mass Spectroscopy:

Electrospray ionization (ESI) mass spectrometry was conducted on a Bruker ESQ 3000 spectrometer. High-resolution ESI-MS was performed on a *Bruker APEX III FTMS* (7 T magnet) (MPI-KoFo) or on a *Thermo LTQ XL Orbitrap* (RUB).

IR Spectroscopy:

IR spectra were obtained using a *Shimadzu IR Affinity – 1S* spectrometer with a *Specac-Quest* ATR unit. Peaks are reported in $\tilde{\upsilon}$ = cm^{-1} and are indicated with w (weak), m (medium), s (strong), vs (very strong) or br (broad).

Elemental Analysis:

CHNS Elemental Analysis was performed on a *vario MICRO cube* from *Elementar Analysentechnik*.

High-Pressure Liquid Chromatography (HPLC):

HPLC measurements were performed at MPI-KoFo & RUB on instruments from *Shimadzu* using chiral columns from *Daicel*, *Dr. Maisch*, and *YMC*. The conditions and retention times (t_R) are specified in the corresponding experiment.

Gas Chromatography (GC):

Gas chromatography (GC) analyses on a chiral stationary phase were performed on *HP Agilent* 6890 and 5890 series instruments (split-mode capillary injection system, flame ionization detector (FID), helium carrier gas). The conditions employed are described in detail for the individual experiments.

Optical Rotation:

Optical rotations were determined at RUB using an *MCP 500* polarimeter from *Anton Paar* at 589 nm (D line) and at 20 °C and at MPI-KoFo with a *Rudolph RA Autopol IV*. Data is reported as $[\alpha]_{\lambda}^{T}$ (temperature *T* [°C] and wavelength λ) accompanied by the concentration [g/100 ml] and solvent.

2. Catalyst synthesis

2.1 Synthesis of known catalysts

Precursor H-(*S*)-**SI-1** was prepared adapting a literature method,[3] but starting from enantiopure BINOL and therefore skipping the chiral resolution step.[4] Boronic esters **SI-2**[5,6] and **SI-3**[7] were prepared as described in the literature (Figure S1).

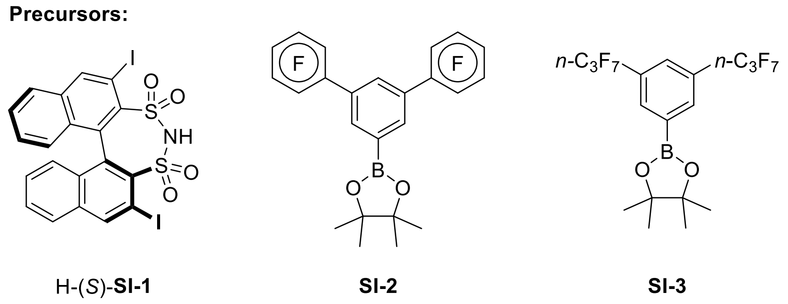

Figure S1: Catalyst precursors.

XB donors **4**-OTf,[8] **4**-BArF$_{24}$ (containing 2 eq. of Et_2O),[8] **SI-4**,[9] **SI-5**,[9] **SI-6**,[9] **SI-7** (containing 0.9 eq. acetone),[10] **SI-8**[10] and **SI-9**[8] were prepared following our published procedures (Figure S2).

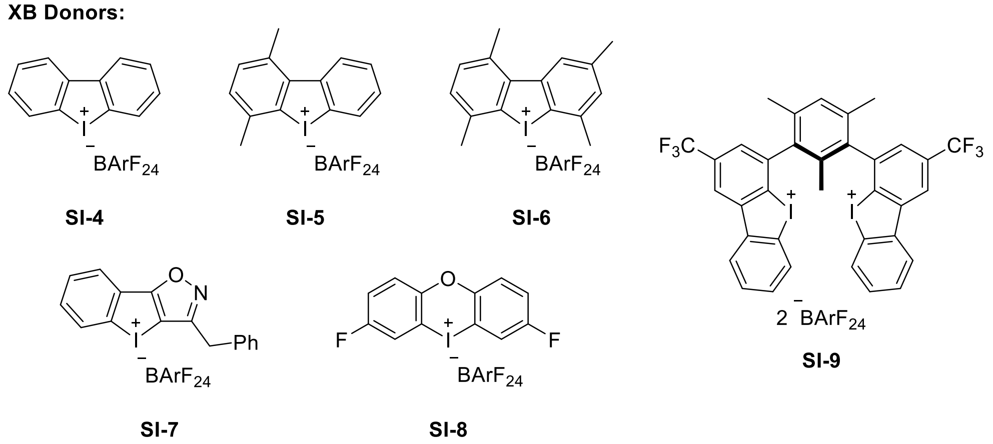

Figure S2: XB catalysts.

The imidodiphosphorimidates H-(*S*,*S*)-**SI-10**,[11] H-(*S*,*S*)-**SI-11**,[11] H-(*S*,*S*)-**SI-12**[11] & H-(*S*,*S*)-**SI-13**[12] were prepared following our previous reports (Figure S3).

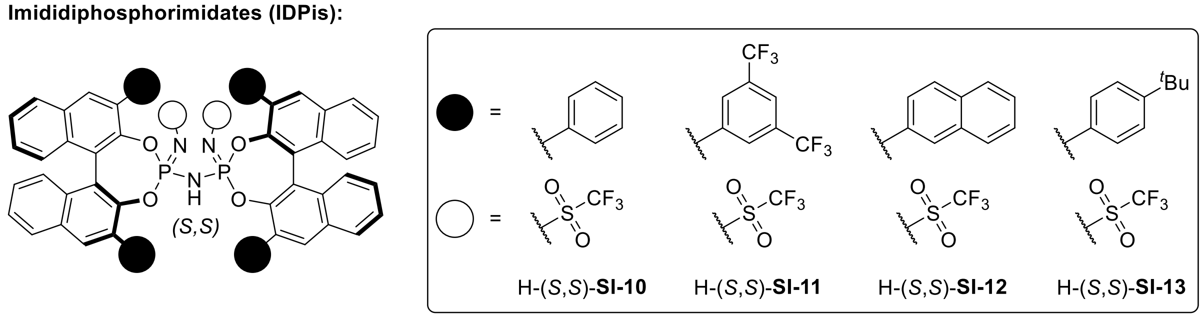

Figure S3: Used IDPis

The DSIs H-(*R*)-**5**,[3] H-(*R*)-**SI-14**,[4] H-(*R*)-**SI-15**,[13] H-(*R*)-**SI-16**,[14] H-(*S*)-**6**,[15] H-(*S*)-**8**[16] & H-(*S*)-**9**[17] were synthesized similar as described in previous publications (Figure S4).

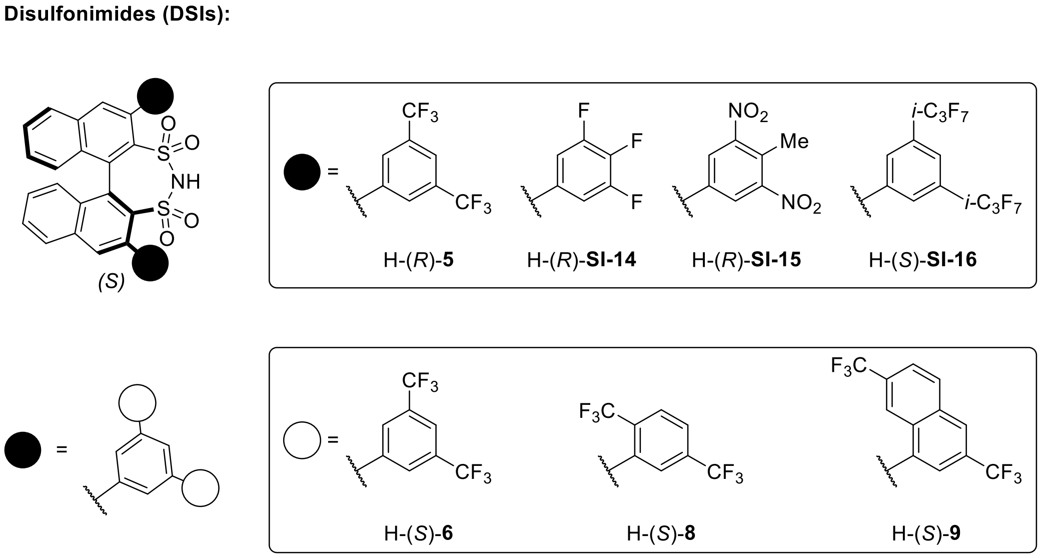

Figure S4: Used literature-known DSIs

2.2 Synthesis of new catalysts

In the following section, the synthesis and characterization of DSIs H-(*S*)-**7** and H-(*S*)-**SI-17**, as well as the bis(iodolium)-DSI complexes **4**-(*S*)-**8** and **4**-(*S*)-**9** (Figure S5) is described.

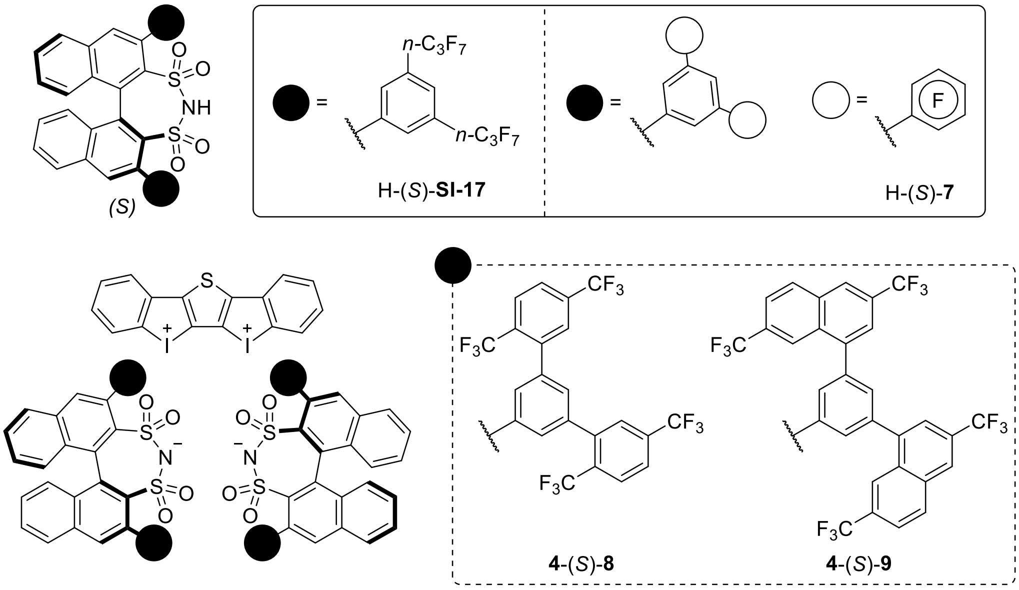

Figure S5: Synthesized new DSIs and bis(iodolium) DSI salts.

2.2.1 Synthesis of DSI H-(*S*)-7

<u>Step 1: Deprotection of Boronic Ester</u>

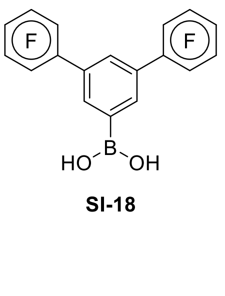

SI-18

This reaction was performed following a literature method by HARTWIG *et al.*.[18] After dissolving 506 mg of the corresponding boronic pinacol ester **SI-2** (944 µmol, 1.0 eq.) in 8 ml THF and 2 ml water, 602 mg sodium periodate (2.82 mmol, 3.0 eq.) were added. The mixture was stirred for 30 min at r.t.

and then 1.9 ml of 1.0 M aqueous HCl (1.9 mmol, 2.0 eq.) were added. The reaction was stirred for 14 h at r.t. and then extracted thrice with EtOAc. The combined organic extracts were washed with water and saturated aqueous NaCl, and then dried over $MgSO_4$. After solvent removal *in vacuo*, the boronic acid **SI-18** was obtained as a colourless solid and used without further purification (419 mg, 923 µmol, 98 %).

Step 2: Suzuki coupling

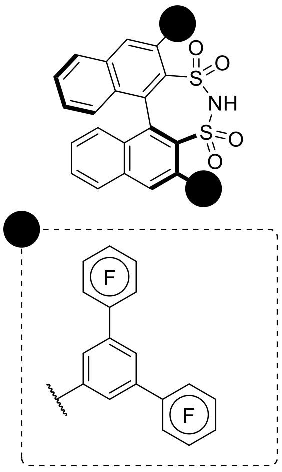

H-(*S*)-**7**

This reaction was performed following a literature method by LEE *et al.*[3] and was conducted under argon. A Schlenk vessel containing 64.8 mg Diiodo-DSI H-(*S*)-**SI-1** (100 µmol, 1.0 eq.) and 136 mg of the boronic acid **SI-18** from the first step (300 µmol, 3.0 eq.) was argonated. To this, 6.0 ml dry THF and 0.6 ml of 2.0 M aqueous K_2CO_3 (1.20 mmol, 12 eq.) were added. The mixture was degassed by 10 min of argon bubbling. Finally, 1.2 mg $Pd(OAc)_2$ (5.3 µmol, 5.3 mol% Pd^{II}) and 25 µL of a 10 % solution of $P(tBu)_3$ in hexanes (1.7 mg, 8.4 µmol, 8.4 mol%) were added, the reaction was heated to 70 °C and stirred at this temperature for 62 h. After cooling to r.t., 10 ml of 1 M aqueous HCl were added, and the mixture was extracted thrice with 10 ml DCM. The combined organic extracts were dried over Na_2SO_4, and the solvent was removed *in vacuo*. The product was purified *via* silica column chromatography (liquid loading, 10 → 30 % EtOAc in *iso*-hexane). After solvent removal *in vacuo*, the residual EtOAc was co-evaporated thrice with DCM and the product dried in HV. The residue was dissolved in 10 ml DCM and filtered through a glass column filled with AMBERCHROM® 50WX8 resin (hydrogen form, washed with 0.125 M H_2SO_4, MeOH, and finally DCM just before use) with the aid of additional DCM. The solvent was removed *in vacuo* and the product was dried in HV overnight yielding the free acid.

Yield: 84.1 mg (69.4 µmol, 69 %) of a colorless solid.

***R*f (25 % EtOAc in *iso*-hexane):** 0.2

^{1}H NMR (501 MHz, Chloroform-*d1*): δ = 8.16 (s, 2H), 8.06 (d, *J* = 8.3 Hz, 2H), 7.76 (s, 2H), 7.73 (t, *J* = 7.6 Hz, 2H), 7.59 (s, 2H), 7.57 (s, 2H), 7.45 (t, *J* = 7.7 Hz, 2H), 7.17 (d, *J* = 8.6 Hz, 2H), 5.40 (bs, 1H) ppm.

^{19}F NMR (471 MHz, Chloroform-*d1*): δ = -142.43 (dd, *J* = 24.4, 10.0 Hz, 4F), -142.57 (dd, *J* = 24.2, 9.7 Hz, 4F), -154.38 (t, *J* = 22.4 Hz, 2F), -154.59 (t, *J* = 22.2 Hz, 2F), -161.56 (td, *J* = 23.0, 9.2 Hz, 4F,), -161.82 (td, *J* = 23.7, 23.2, 9.3 Hz, 4F) ppm.

^{13}C{^{1}H} NMR (126 MHz, Chloroform-*d1*): δ = 145.8 – 143.2 (m), 142.1 – 139.4 (m), 140.1, 138.6, 139.3 – 136.6 (m), 135.0, 134.5, 134.1, 133.3, 132.1, 132.1, 131.5, 131.1, 130.3, 129.0, 128.8, 128.2, 127.0, 126.2, 115.1 – 114.6 (m) ppm.

HRMS (ESI-) (m/z): calc. for $C_{56}H_{16}F_{20}N_1O_4S_2$ [M-H]-: 1210.02069; found: 1210.02176.

2.2.2 Synthesis of DSI H-(*S*)-SI-17

Step 1: Deprotection of Boronic Ester

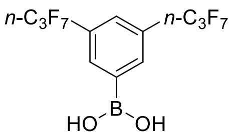

SI-19

This reaction was performed following a literature method by HARTWIG *et al.*.[18] After dissolving 161 mg of the corresponding boronic pinacol ester **SI-2** (298 µmol, 1.0 eq.) in 2.4 ml THF and 0.6 ml water, 191 mg sodium periodate (893 µmol, 3.0 eq.) were added. The mixture was stirred for 30 min at r.t. and then 0.6 ml of 1.0 M aqueous HCl (0.60 mmol, 2.0 eq.) were added. The reaction was stirred for 15 h at r.t. and then extracted thrice with EtOAc. The combined organic extracts were washed with water and saturated aqueous NaCl, and then dried over $MgSO_4$. After solvent removal *in vacuo*, the boronic acid **SI-19** was obtained as an off-white solid and used without further purification (136 mg, 298 µmol, quant. yield).

Step 2: Suzuki coupling

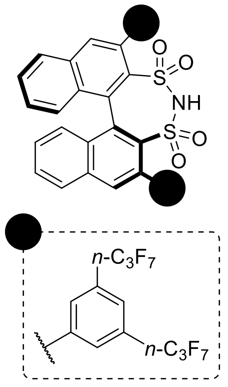

H-(*S*)-**SI-17**

This reaction was performed following a literature method by LEE *et al.*[3] and was conducted under argon. Into a flame-dried Schlenk vessel, 135 mg of the boronic acid **SI-19** from step 1 (295 µmol, 3.0 eq.) dissolved in 6.0 ml dry THF were added. To this 64.5 mg Diiodo-DSI H-(*S*)-**SI-1** (99.7 µmol, 1.0 eq.) and 0.6 ml of a 2.0 M aqueous K_2CO_3 solution (1.20 mmol, 12 eq.) were added. Argon was bubbled through the mixture for 10 min under stirring to degas. Finally, 1.0 mg $Pd(OAc)_2$ (4.5 µmol, 5 mol%) and 30 µL of a 10% solution of $P(^tBu)_3$ in hexanes (2.0 mg, 10 µmol, 10 mol%) were added and it was heated up to 66 °C and stirred at this temperature for 14 h. After cooling to r.t., 10 ml of a 1 M aqueous HCl solution were added, and it was extracted thrice with 10 ml DCM. The combined organic extracts were dried over Na_2SO_4, filtered, celite was added and the solvent removed *in vacuo*. It was purified by silica column chromatography (gradient: 0-3% MeOH in DCM). After solvent removal *in vacuo*, the residue was dissolved in DCM and filtered through a glass column filled with Amberchrom 50WX8 resin (hydrogen form, washed with 0.125 M H_2SO_4, MeOH and finally DCM just before use) with the aid of additional DCM. After solvent removal *in vacuo* and drying overnight in HV, no pure acid was yielded. It was then dissolved in DCM and washed thrice with 6 M aqueous HCl. The solvent of the organic phase was removed *in vacuo,* and it was coevaporated thrice with toluene. After drying in HV the product was obtained clean as free acid.

Yield: 92.5 mg (75.8 µmol, 76 % yield) of a beige solid.

***R*f (5 % MeOH in DCM):** 0.2

^{1}H NMR (501 MHz, Chloroform-*d1*): δ = 8.08 (d, *J* = 8.2 Hz, 2H), 8.05 (s, 2H), 7.97 (s, 2H), 7.89 (s, 2H), 7.86 (s, 2H), 7.78 (t, *J* = 7.5 Hz, 2H), 7.52 (t, *J* = 7.7 Hz, 2H), 7.25 (d, *J* = 9.3 Hz, 2H), 5.40 (bs, 1H) ppm.

^{19}F NMR (471 MHz, Chloroform-*d1*): δ = -79.54 – -80.24 (m, 12F), -111.59 – -112.42 (m, 8F), -126.01 – -126.40 (m, 8F) ppm.

$^{13}C\{^1H\}$ NMR (126 MHz, Chloroform-*d1*): δ = 141.0, 138.9, 134.4, 134.2, 133.8, 132.5 – 132.2 (m), 132.3, 131.9, 130.7, 130.9 – 130.5 (m), 129.8 (t, *J* = 25.1 Hz), 129.5, 129.1 (t, *J* = 25.2 Hz), 128.9, 128.3, 125.0 – 124.7 (m), 119.83 – 105.74 (several multiplets in this area, corresponding to fluorinated C^{sp3}) ppm.
HRMS (ESI-) (m/z): calc. for $C_{44}H_{16}F_{28}N_1O_4S_2$ [M-H]-: 1218.00791; found: 1218.00836.

2.2.3 Synthesis of Bis(iodolium)-DSI-Salt 4-(*S*)-8

Step 1: Deprotonation

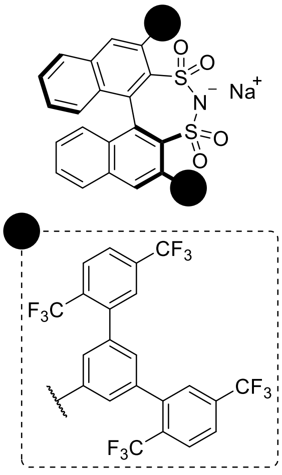

Na-(*S*)-**8**

First, 369 mg DSI H-(*S*)-**8** (284 µmol, 1.0 eq.) were dissolved in 30 ml DCM (0.01 M). After the addition of 1.51 g Na_2CO_3 (14.2 mmol, 50 eq.), the suspension was stirred at r.t. for 30 min, filtered and the solvent removed *in vacuo*. The purity of the compound was checked *via* ^{1}H and ^{19}NMR spectroscopy and it was used without further characterization.

Yield: 387 mg (273 µmol, 96 %) of a slightly beige solid.

^{1}H NMR (400 MHz, Methylene Chloride-*d2*): δ = 8.00 (d, *J* = 8.3 Hz, 2H), 7.96 (s, 2H), 7.80 (d, *J* = 8.2 Hz, 2H), 7.68 (dd, *J* = 17.4, 9.1 Hz, 10H), 7.58 (ddd, *J* = 8.1, 6.8, 1.1 Hz, 2H), 7.47 (d, *J* = 1.7 Hz, 4H), 7.23 (s, 2H), 7.19 (ddd, *J* = 8.3, 6.8, 1.3 Hz, 2H), 6.97 (d, *J* = 8.6 Hz, 2H) ppm.

^{19}F NMR (376 MHz, Methylene Chloride-*d2*): δ = -57.65 (s, 3F), -57.71 (s, 3F), -63.48 (s, 3F), -63.64 (s, 3F) ppm.

Step 2: Metathesis

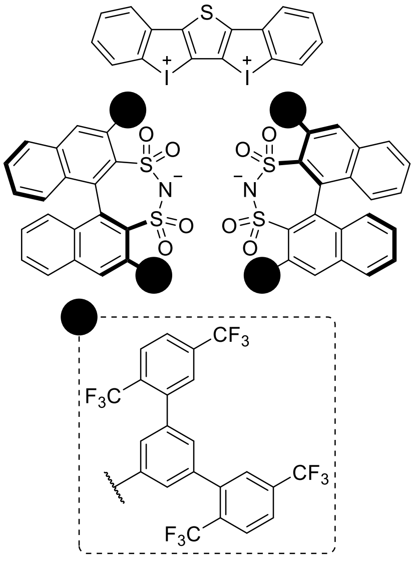

4-(*S*)-**8**

In a microwave vial, 101 mg of bis(iodolium) triflate **4**-OTf (128 µmol, 1.0 eq.) and 365 mg Na-(*S*)-**8** (Step 1, 258 µmol, 2.0 eq.) were suspended in 13 ml dry MeOH (0.01 M). The vial was placed in a *CEM Discover SP* microwave reactor and the mixture was stirred in dynamic mode at 50 °C for 2 h under microwave irradiation. After cooling to r.t., the solvent was removed *in vacuo*. The residue was suspended in 13 ml DCM, shortly sonicated and stored in the freezer (-30 °C) over the weekend. The suspension was filtered through a PP syringe filter (0.22 µm) and the filter was washed twice with 3 ml DCM. The solvent of the filtrate was removed *in vacuo*. The residue was dissolved in a minimal amount of DCM and precipitated with *n*-pentane. The precipitate was collected *via* vacuum filtration (paper filter, Buchner funnel), washed thrice with *n*-pentane, and dried in HV.

Yield: 332 mg (101 µmol, 79 %) of a slightly grey solid.

^{1}H NMR (400 MHz, DMSO-*d6*): δ = 8.20 (dd, *J* = 7.8, 1.3 Hz, 2H), 8.15 – 8.09 (m, 8H), 8.08 – 7.91 (m, 18H), 7.87 – 7.70 (m, 14H), 7.58 (t, *J* = 7.6 Hz, 4H), 7.55 – 7.48 (m, 6H), 7.34 – 7.26 (m, 8H), 6.92 (d, *J* = 8.6 Hz, 4H) ppm.

^{19}F NMR (376 MHz, DMSO-*d6*): δ = -56.08 (s, 24 F), -61.82 (s, 12 F), -61.91 (s, 12 F) ppm. (< 0.1 eq. $^-$OTf left)

$^{13}C\{^{1}H\}$ NMR (101 MHz, DMSO-*d6*): δ = 148.1, 141.3, 140.8, 137.8, 136.8, 136.2, 135.6, 135.5, 134.9, 132.7, 132.3 (q, *J* = 32.4 Hz), 131.9, 131.1, 130.8, 130.7 (q, *J* = 30.4 Hz), 130.4, 129.3, 128.8, 128.2, 127.9 – 127.6 (broad, overlapping signals), 127.5, 127.2 – 127.0 (broad, overlapping signals), 126.3, 126.0, 125.2, 123.3 (q, *J* = 274.7 Hz), 123.2 (q, *J* = 273.0 Hz), 110.8.

HRMS (ESI+) (m/z): calc. for $C_{80}H_{36}F_{24}N_1O_4S_3I_2$ [M-DSI]$^+$: 1879.9513; found: 1879.9483.

MS (ESI-) (m/z): calc. for $C_{64}H_{28}F_{24}N_1O_4S_2$ [DSI]$^-$: 1394.1, found: 1394.1.

ATR-IR [$\tilde{\upsilon}$ = cm^{-1}]: 515 (m), 548 (m), 646 (m), 664 (w), 702 (m), 712 (m), 719 (m), 752 (m), 785 (m), 808 (w), 841 (m), 885 (w), 901 (w), 1038 (s), 1082 (s), 1121 (s), 1177 (m), 1263 (m), 1312 (s), 1408 (w).

EA: calc.: 52.79 %C, 1.97 %H, 0.86 %N, 4.89 %S, found: 52.42 %C, 2.06 %H, 0.87 %N, 5.79 %S.

$[\alpha]_D^{20}$ = -170° (c = 0.70, $CHCl_3$)

2.2.4 Synthesis of Bis(iodolium)-DSI-Salt 4-(*S*)-9

Step 1: Deprotonation

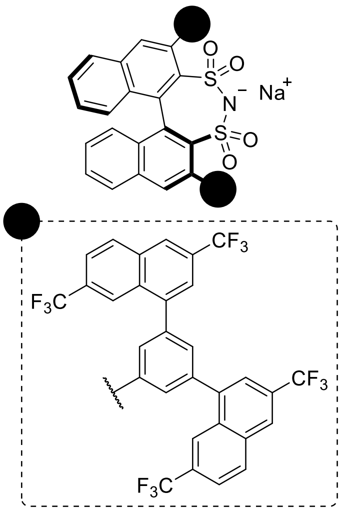

Na-(*S*)-**9**

First, 146 mg DSI H-**S-(9)** (91.5 µmol, 1.0 eq.) were dissolved in 9 ml DCM (0.01 M). After the addition of 491 mg Na_2CO_3 (4.63 mmol, 51 eq.), the suspension was stirred at r.t. for 30 min, filtered and the solvent removed *in vacuo*. The purity of the compound was checked *via* ^{1}H and ^{19}NMR spectroscopy and it was used without further characterization.

Yield: 146 mg (90.2 µmol, 99 %) of a beige solid.

^{1}H NMR (400 MHz, Acetone-*d6*): δ = 8.74 (s, 4H), 8.60 – 8.36 (m, 8H), 8.19 – 7.82 (m, 16H), 7.75 (s, 2H), 7.51 (t, *J* = 7.5 Hz, 2H), 7.25 – 7.13 (m, 2H), 7.02 (d, *J* = 8.6 Hz, 2H) ppm.

^{19}F NMR (376 MHz, Acetone-*d6*): δ = -62.87 – -63.14 (m) ppm.

Step 2: Metathesis

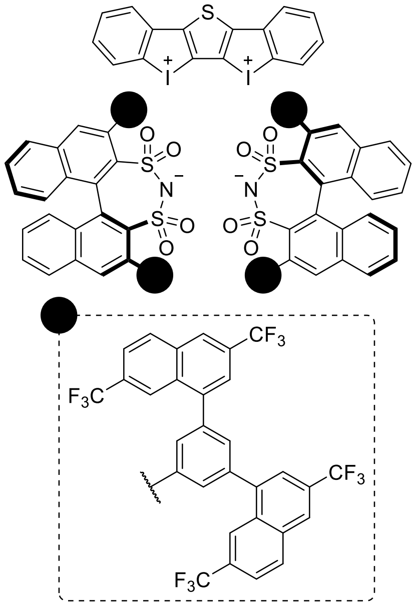

4-(*S*)-**9**

In a microwave vial, 35.2 mg of bis(iodolium) triflate **4**-OTf (44.9 µmol, 1.0 eq.) and 146 mg Na-(*S*)-**9** (Step 1, 90.2 µmol, 2.0 eq.) were suspended in 4.5 ml dry MeOH (0.01 M). The vial was placed in a *CEM Discover SP* microwave reactor and the mixture was stirred in dynamic mode at 50 °C for 2 h under microwave irradiation. After cooling to r.t., the solvent was removed *in vacuo*. The residue was suspended in 4.5 ml DCM, shortly sonicated and stored in the freezer (-30 °C) for 1 h. The suspension was filtered through a PP syringe filter (0.22 µm) and the filter was washed twice with 1 ml DCM. The solvent of the filtrate was removed *in vacuo*. The residue was dissolved in a minimal amount of DCM and precipitated with *n*-pentane. The precipitate was collected *via* vacuum filtration (paper filter, Buchner funnel), washed thrice with *n*-pentane, and dried in HV.

Yield: 94.2 mg (25.6 µmol, 57 %) of a brownish solid.

^{1}H NMR (400 MHz, DMSO-*d6*): δ = 8.64 (s, 8H), 8.60 – 8.38 (m, 14H), 8.31 – 7.42 (m, 50H), 7.28 (t, *J* = 7.7 Hz, 4H), 6.88 (d, *J* = 8.7 Hz, 4H) ppm.

^{19}F NMR (376 MHz, DMSO-*d6*): δ = -60.78 – -62.21 (m) ppm. The multiplet consists of two sharp singlets and one broad signal. (< 0.1 eq. $^{-}$OTf left)

^{13}C{^{1}H} NMR (101 MHz, DMSO-*d6*): δ = 142.0, 141.6, 137.2, 136.7, 136.4, 135.1, 133.8, 132.8, 132.1, 131.9, 131.4, 131.2, 130.8, 130.1, 129.6, 129.3, 129.0, 128.0, 127.8, 127.7, 127.5, 127.3, 125.7, 125.3, 125.3, 125.1, 123.7, 123.1, 122.7, 122.6, 122.6, 122.4, 119.9, 119.7, 109.6.

Due to the complexity of the spectrum, which may be attributed to rotamers, a clear classification of the present signals was not possible. Given are clear peaks. Some of these may be part of quartets/multiplets. Additionally, a signal at 147.8 is present but only borderline visible. In the area of 122 – 133 ppm, the spectrum is especially complex and multiplets visible "under" the given signals.

HRMS (ESI+) (m/z): calc. for $C_{96}H_{44}F_{24}N_{1}O_{4}S_{3}I_{2}$ [M-DSI]$^{+}$: 2080.0139; found:.2080.0089.

MS (ESI-) (m/z): calc. for $C_{80}H_{36}F_{24}N_{1}O_{4}S_{2}$ [DSI]$^{-}$: 1594.2, found: 1594.1.

ATR-IR [$\tilde{\upsilon}$ = cm^{-1}]: 515 (m), 530 (m), 548 (m), 633 (w), 648 (m), 694 (m), 725 (m), 733 (m), 754 (m), 773 (m), 816 (w), 849 (w), 893 (m), 1067 (s), 1119 (s), 1165 (m), 1206 (w), 1238 (w), 1258 (m), 1275 (m), 1310 (s), 1385 (w).

$[\alpha]_D^{20}$ = -201° (c = 0.60, $CHCl_3$)

3. Screening

3.1 General Preparation of $N(nBu)_4$-Salts

The corresponding acid was weighed into an appropriate vessel and MeOH (c = 10 mM) was added. If necessary, the same amount of DCM was added for complete dissolution. To this, 1.0 eq. of $N(nBu)_4$-OH was added in one portion as 1.0 M solution in MeOH. The reaction was stirred at r.t. for 15 min and then the solvent was removed *in vacuo*. The formed water was co-evaporated thrice with dry toluene and afterward, the residue was dried on a rotary evaporator and in HV yielding the chiral salt **N^nBu_4-Z*** in up to quantitative yield. The successful transformation was checked *via* ^1H and if appropriate ^{19}F and ^{31}P NMR spectroscopy. These salts were used without further purification and characterization in the catalysis reactions.

3.2 Screening Procedure for Diels-Alder Reaction

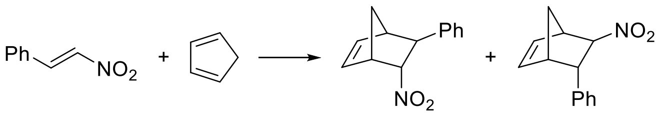

1a **2** *endo*-**3a** *exo*-**3a**

The reaction was performed in oven-dried glassware under argon using dry solvents. It was conducted on a 25 µmol scale at 100 mM concentration. At lower concentrations, a 12.5 µmol scale was chosen in most cases.

In an oven-dried 1.5 ml GC vial, *trans*-β-nitrostyrene (**1a**, 1.0 eq.), the corresponding XB catalyst and/or additives, as well as toluene at given concentration were added (this process can also be done using stock solutions). Then, the reaction was stirred at r.t. for 30 min. Afterward, cyclopentadiene (**2**, 10 eq.) was added. After stirring at r.t. for 23 h, the catalyst was deactivated by the addition of a solution of $N(nBu)_4$-Br (0.5 or 1.0 M, 1.0 eq.) in $CHCl_3$ and for reactions employing acids additionally a drop of NEt_3 ($\geq$ 1.0 eq.) was added to quench these. As NMR standard a solution of methyl 3,5-dinitrobenzoate (0.5 or 1.0 M, 1.0 eq.) in $CHCl_3$ was added. The mixture was stirred for further 5-10 min and then 500 µL $CDCl_3$ were added, and all was mixed. Now, 500 µL of the mixture was transferred into an NMR tube, and a ^1H NMR spectrum was measured to determine the yield by integration of the characteristic triplet signal of *endo*-**3a** at ~5.15 ppm ($C(NO_2)$-**H**, 1H) against the signal of the NMR standard at ~4.3 ppm (3H). The remaining mixture was used for pTLC (10x10 cm, 250 µm, eluent: 5 % EtOAc, in *n*-pentane, R_f = 0.6). The product line was scraped off, suspended in *n*-heptane, filtered through a syringe filter, and analyzed via chiral HPLC.

Chiral HPLC (MPI): (Daicel Chiralpak IA-3 (3 µm), 220 nm, 25 °C, 1 % *i*PrOH in heptane, 0.5 ml/min):

endo-**3a (major)**: t_{RA1} = 15.7 min, t_{RA2} = 23.3 min.

exo-**3a (minor)**: t_{RB1} = 14.9 min (slight overlap with t_{RA1}), t_{RB2} = 19.0 min.

Chiral HPLC (RUB): (YMC CHIRAL ART Amylose-SA S-5 µm, 220 nm, 25 °C, 1 % *i*PrOH in heptane, 1.0 ml/min):

endo-**3a (major)**: t_{RA1} = 6.5 min, t_{RA2} = 9.4 min.

exo-**3a (minor)**: t_{RA1} = 6.2 min, t_{RB2} = 8.0 min.

3.3 Additional Screening Results

3.3.1 Effect of the XB catalyst structure

For this comparison, the catalysts were used as salts with the weakly coordinating counteranion $BArF_{24}$ and at 20 mol% concentration of iodonium centres for better comparison between mono- and bidentate systems (Table S1). The reaction was done as described in section 3.2 (100 mM, 23 h, toluene at r.t.). The prototypical monodentate catalyst **SI-4** is also able to catalyze this reaction (47 % yield). By implementation of an *ortho*-methyl group (**SI-5**), the activity is reduced (29 % yield). The addition of a second one diminished it to background reactivity (13 % yield). This effect is known, as these groups can block the halogen bonding axis.[9] Of course, the electronic effect of the methyl groups also plays a role. The tuned[10,19] monodentate catalysts **SI-7** and **SI-8** exhibit increased activity (67 and 59 % yield, respectively) with comparable results to the bidentate catalyst **4**-$BArF_{24}$. The bidentate catalyst **SI-9**, which was previously reported to be a weaker XB donor compared to catalyst **4**-$BArF_{24}$,[8] also performed well (66 % yield). As the best results in terms of yield were accomplished with bis(iodolium) DSI salts like **4**-(*S*)-**8**, which contain more coordinating counteranions than $BArF_{24}$, we suspect that **4**-$BArF_{24}$ may have selectivity issues.

In combination with 20 mol% of the additive N(*n*Bu)$_4$-(*R*)-**5**, only catalyst **4**-$BArF_{24}$ showed significant activity and enantioenriched product. This shows that the core structure is important for efficient asymmetric catalysis of this reaction.

Table S1: Effect of the XB catalyst. The reactions were performed as described in section 3.2.

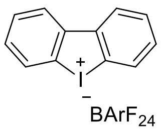

SI-4

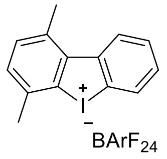

SI-5

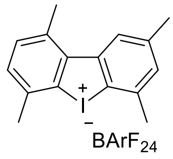

SI-6

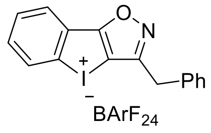

SI-7

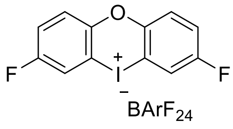

SI-8

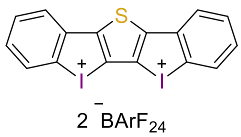

4-$BArF_{24}$

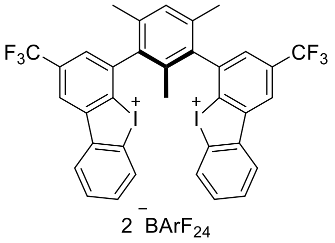

SI-9

Entry	Catalyst (mol%)	Additive (20 mol%)	Yield (%)	e.r.
1	-	-	12	-
2	**4**-$BArF_{24}$ (1)	-	19	-
3	**4**-$BArF_{24}$ (5)	-	44	-
4	**4**-$BArF_{24}$ (10)	-	60	-
5	**4**-$BArF_{24}$ (10)	N(*n*Bu)$_4$-(*R*)-**5**	37	75:25
6	**SI-4** (20)	-	47	-
7	**SI-4** (20)	N(*n*Bu)$_4$-(*R*)-**5**	16	51:49
8	**SI-5** (20)	-	29	-
9	**SI-6** (20)	-	13	-
10	**SI-7** (20)	-	67	-
11	**SI-7** (20)	N(*n*Bu)$_4$-(*R*)-**5**	15	51:49
12	**SI-8** (20)	-	59	-
13	**SI-8** (20)	N(*n*Bu)$_4$-(*R*)-**5**	16	51:49
14	**SI-9** (10)	-	66	-
15	**SI-9** (10)	N(*n*Bu)$_4$-(*R*)-**5**	20	50:50

3.3.2 Screening of different counteranions

Besides DSIs also imidodiphosphorimidates (IDPis) were tested as counteranions in this reaction. In these cases, the activity was reduced and therefore the reaction was stirred over 65 h. The reaction was performed mostly as described as described in section 3.2 (100 mM, toluene, r.t.), but the signals of the $N(nBu)_4$ ions (of the additive and the added bromide salt) were used as internal standard. Unfortunately, only one tested IDPi showed a yield higher than the background reaction. In all cases racemic product formed.

Table S2: Screening of IDPi counteranions. Changes to procedure from section 3.2 are given or described above.

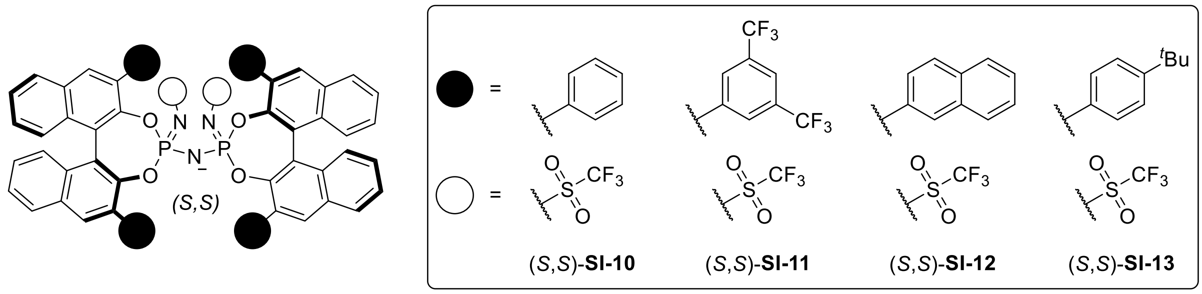

Entry	Catalyst (10 mol%)	Additive (20 mol%)	Yield (%) after 65 h	e.r.
1	-	-	32	-
2	**4**-BArF24	N(*n*Bu)4-(*S*,*S*)-**SI-10**	26	52:48-
3	**4**-BArF24	N(*n*Bu)4-(*S*,*S*)-**SI-11**	49	48:52
4	**4**-BArF24	N(*n*Bu)4-(*S*,*S*)-**SI-12**	33	51:49
5	**4**-BArF24	N(*n*Bu)4-(*S*,*S*)-**SI-13**	35	51:49

Beside the counteranions mentioned in the main paper, other DSIs were screened. The reactions were done as described in section 3.2 (100 mM, toluene, r.t.). In the two cases stirring for 20 h, a $N(nBu)_4$-signal was used as NMR standard. The anions showed low activity and enantioselectivity (Table S3).

Table S3: Screening of IDPi counteranions. Changes to procedure from section 3.2 are given or described above.

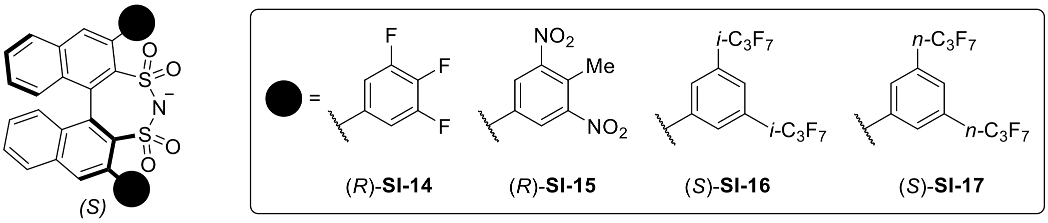

Entry	Catalyst (10 mol%)	Additive (20 mol%)	Time (h)	Yield (%)	e.r.
1	**4**-BArF24	N(*n*Bu)4-(*R*)-**SI-14**	20	14	52:48
2	**4**-BArF24	N(*n*Bu)4-(*R*)-**SI-15**	20	16	56:44
3	**4**-BArF24	N(*n*Bu)4-(*S*)-**SI-16**	23	21	49:51
4	**4**-BArF24	N(*n*Bu)4-(*S*)-**SI-17**	23	21	47:53

3.3.3 Influence of solvent

Different solvents were screened using the N(*n*Bu)$_4$-(*S*)-**9** and the typical screening procedure (23 h at r.t.). As defined catalyst **4**-(*S*)-**9**, this counteranion showed good results (Table S4, entries 1 & 2) and as additive it performed slightly worse (entries 3 & 4). The solvent screening was done at 50 mM concentration. Other aromatic solvents as well as MTBE showed worse results in terms of yield and enantioselectivity (entries 5-8). In cyclohexane, no enantioselectivity was observed (entry 9).

Table S4: Solvent screening. Changes to procedure from section 3.2 are given or described above.

Entry	Catalyst (10 mol%)	Additive (20 mol%)	Solvent	Conc. (mM)	Yield (%)	e.r.
1	**4**-(*S*)-**9**	-	Toluene	100	> 95	88:12
2	**4**-(*S*)-**9**	-	Toluene	50	> 95	89:11
3	**4**-BArF$_{24}$	N(*n*Bu)$_4$-(*S*)-**9**	Toluene	100	69	85:15
4	**4**-BArF$_{24}$	N(*n*Bu)$_4$-(*S*)-**9**	Toluene	50	55	85:15
5	**4**-BArF$_{24}$	N(*n*Bu)$_4$-(*S*)-**9**	*m*-Xylene	50	35	80:20
6	**4**-BArF$_{24}$	N(*n*Bu)$_4$-(*S*)-**9**	Mesitylene	50	38	73:27
7	**4**-BArF$_{24}$	N(*n*Bu)$_4$-(*S*)-**9**	Cumene	50	40	76:24
8	**4**-BArF$_{24}$	N(*n*Bu)$_4$-(*S*)-**9**	MTBE	50	16	72:28
9	**4**-BArF$_{24}$	N(*n*Bu)$_4$-(*S*)-**9**	Cyclohexane	50	26	51:49

3.3.4 Influence of temperature

The influence of the temperature was tested using the additive method as well as the defined catalyst **4**-(*S*)-**9**. In all cases the typical method was used (Toluene, 100 mM). In the latter case (entries 8 to 10), a reduced catalyst loading of 5 mol% was used as this did not show worse results at r.t.. In this case and when the additive method was tested with three different anions (entries 1-7), the lower temperature reduced or did not improve the enantioselectivity of the reaction.

Table S5: Temperature screening. Changes to procedure from section 3.2 are given or described above.

Entry	Catalyst (mol%)	Additive (20 mol%)	Temperature	Time (h)	Yield (%)	e.r.
1	**4**-BArF$_{24}$ (10)	N(*n*Bu)$_4$-(*R*)-**5**	r.t.	23	37	75:25
2	**4**-BArF$_{24}$ (10)	N(*n*Bu)$_4$-(*R*)-**5**	10 °C	68	32	73:27
3	**4**-BArF$_{24}$ (10)	N(*n*Bu)$_4$-(*R*)-**5**	0 °C	68	11	69:31
4	**4**-BArF$_{24}$ (10)	N(*n*Bu)$_4$-(*S*)-**7**	r.t.	23	35	76:24
5	**4**-BArF$_{24}$ (10)	N(*n*Bu)$_4$-(*S*)-**7**	0 °C	68	14	69:31
6	**4**-BArF$_{24}$ (10)	N(*n*Bu)$_4$-(*S*)-**8**	r.t.	23	46	86:14
7	**4**-BArF$_{24}$ (10)	N(*n*Bu)$_4$-(*S*)-**8**	0 °C	68	19	86:14
8	**4**-(*S*)-**9** (10)	-	r.t.	23	> 95	88:12
9	**4**-(*S*)-**9** (5)	-	r.t.	23	92	88:12
10	**4**-(*S*)-**9** (5)	-	0-5 °C	92	18	85:15

4. Isolation and Scope

4.1 Preparative Procedure for Diels-Alder Reaction

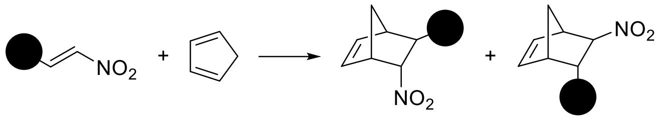

1 **2** *endo*-**3** *exo*-**3**

In an oven-dried 1.5 ml GC vial (50 µmol scale) or a flame-dried Schlenk tube (100 µmol scale), substituted nitrostyrene **1** (1.0 eq.), bis(iodolium) DSI **4**-(*S*)-**8** (5-10 mol%), as well as toluene (50 mM) were added. Then the mixture was stirred at r.t. for 30 min, followed by the addition of cyclopentadiene (**2**, 10 eq.). After stirring at r.t. for 23 h, the catalyst was deactivated by the addition of a 0.5 M solution N(*n*Bu)$_4$-Br in $CHCl_3$ (1.0 eq.). The suspension was stirred for further 5 min and then purified *via* pTLC (20x20 cm, 250 µm at 50 µmol scale or 20x20 cm, 2 mm at 100 µmol scale). Racemic reference compounds of the endo:exo mixtures were synthesized thermically in dichloroethane. The absolute configuration of *endo*-**3a** was determined by Vibrational Circular Dichroism (VCD) spectroscopy (see section 6). For clarity, in the following the same enantiomers are depicted for the other products even though in these cases the absolute configurations are unknown.

4.2 (-)-(1*R*,4*S*,5*R*,6*S*)-*Endo*-5-nitro-6-phenylbicyclo[2.2.1]hept-2-ene (*endo*-3a)

4.2.1 Isolation at 50 µmol scale and 50 mM concentration with 10 mol% catalyst loading:

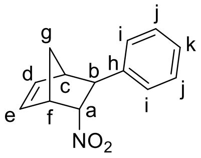

endo-**3a**

Used were 7.45 mg *trans*-β-nitrostyrene (**1a**, 50.0 µmol, 1.0 eq.), 16.5 mg **4**-(*S*)-**8** (5.03 µmol, 10 mol%)**,** 42 µl cyclopentadiene (**2**, 0.50 mmol, 10 eq.) in 1.0 ml dry toluene (50 mM). Eluent (pTLC): 5 % EtOAc in *n*-pentane.

Yield: 10.1 mg (46.8 µmol, 94 %) of a yellowish oil.

R_f (5 % EtOAc in *n*-pentane): 0.6

^{1}H NMR (300 MHz, Chloroform-*d1*): δ = 7.40 – 7.31 (m, 4H, C[i]-**H**, C[j]-**H**), 7.30 – 7.22 (m, 1H, C[k]-**H**), 6.62 (dd, *J* = 5.7, 3.2 Hz, 1H, C[d]-**H**), 6.14 (dd, *J* = 5.7, 2.7 Hz, 1H, C[e]-**H**), 5.02 (t, *J* = 4.0 Hz, 1H, C[a]-**H**), 3.62 (hept, *J* = 1.4 Hz, 1H C[f]-**H**), 3.46 (dd, *J* = 4.1, 2.7 Hz, 1H, C[b]-**H**), 3.19 (dq, *J* = 3.3, 1.6 Hz, 1H, C[c]-**H**), 1.90 (dt, *J* = 9.3, 1.5 Hz, 1H, C[g]-**H**), 1.75 (dq, *J* = 9.3, 2.1 Hz, 1H, C[g]-**H**) ppm.

^{13}C{^{1}H} NMR (75 MHz, Chloroform-*d1*): δ = 141.5 (**C**[k]), 140.8 (**C**[d]), 133.3 (**C**[e]), 129.0 (**C**[i] or (**C**[j]), 127.5 (**C**[i] or (**C**[j]), 127.1 (**C**[k]), 92.4 (**C**[a]), 49.1 (**C**[b]), 48.2 (**C**[c]), 48.0 (**C**[f]), 46.3 (**C**[g]) ppm.

endo:exo: >100:1

HRMS: (ESI+) (m/z): calc. for $C_{13}H_{14}N_1O_2$ $[M+H]^+$: 216.1025; found: 216.1020.

Chiral HPLC: (YMC CHIRAL ART Amylose-SA S-5 µm, 220 nm, 20 °C, 1 % *i*PrOH in heptane, 1.0 ml/min): t_{R1} (major) = 6.6 min, t_{R2} (minor) = 9.8 min, e.r. = 93:7, 86 % e.e.

4.2.2 Isolation at 50 µmol scale and 12.5 mM concentration with 10 mol% catalyst loading:

Used were 7.38 mg *trans*-β-nitrostyrene (**1a**, 49.5 µmol, 1.0 eq.), 16.4 mg **4**-(*S*)-**8** (5.00 µmol, 10 mol%), 42 µl cyclopentadiene (**2**, 0.50 mmol, 10 eq.) in 4.0 ml dry toluene (12.5 mM). Eluent (pTLC): 5 % EtOAc in *n*-pentane. Due to the high amount of solvent, two pTLC plates were used simultaneously.

Yield: 5.97 mg (27.7 µmol, 56 %) of a yellowish oil.

R_f (5 % EtOAc in *n*-pentane): 0.6

NMR spectral data as above.

endo:exo: >100:1

Chiral HPLC: (YMC CHIRAL ART Amylose-SA S-5 µm, 220 nm, 25 °C, 1 % *i*PrOH in heptane, 1.0 ml/min): t_{R1} (major) = 6.6 min, t_{R2} (minor) = 9.7 min, e.r. = 94.5:5.5, 89 % e.e.

4.2.3 Isolation at 100 µmol scale and 12.5 mM concentration with 10 mol% catalyst loading:

Used were 14.9 mg *trans*-β-nitrostyrene (**1a**, 99.9 µmol, 1.0 eq.), 32.6 mg **4**-(*S*)-**8** (10.0 µmol, 10 mol%), 85 µl cyclopentadiene (**2**, 1.0 mmol, 10 eq.) in 8.0 ml dry toluene (12.5 mM). Eluent (pTLC): 5 % EtOAc in *n*-pentane. Due to the high amount of solvent, two pTLC plates were used simultaneously.

Yield: 11.9 mg (55.3 µmol, 55 %) of a yellowish oil.

R_f (5 % EtOAc in *n*-pentane): 0.3

NMR spectral data as above.

endo:exo: >100:1

Chiral HPLC: (YMC CHIRAL ART Amylose-SA S-5 µm, 220 nm, 25 °C, 1 % *i*PrOH in heptane, 1.0 ml/min): t_{R1} (major) = 6.6 min, t_{R2} (minor) = 9.7 min, e.r. = 95:5, 90 % e.e.

$[\alpha]_D^{20}$ = -116° (c = 0.77, $CHCl_3$)

The absolute configuration was determined by VCD spectroscopy (see section 6).

4.2.4 Isolation at 0.76 mmol scale and 12.5 mM concentration with 10 mol% catalyst loading:

In a flame-dried 100 ml Schlenk flask 114 mg *trans*-β-nitrostyrene (**1a**, 0.76 mmol, 1.0 eq.) and 250 mg of **4**-(*S*)-**8** (0.076 mmol, 10 mol%) were added, the flask's gaseous content was evacuated and backfilled with argon. Then 60.0 ml of dry toluene (12.5 mM) were added, and the mixture was stirred at r.t. for 30 min, followed by the addition of 0.635 ml cyclopentadiene (**2**, 7.6 mmol, 10 eq.). After stirring at r.t. for 54 h, the catalyst was precipitated from the reaction mixture by adding an excess of pentane (160 ml). The suspension was stirred for further 10 min and then centrifugated. The liquid phase was separated, and the solvents were evaporated in vacuo. The obtained residue was dissolved in DCM and purified via column chromatography eluting with EtOAc:pentane mixture from 5 to 10 % yielding clean *endo*-**3a**. The solid phase was collected as well and dried in high vacuum for 2 days delivering the recovered catalyst 182 mg (73 %) **4**-(*S*)-**8** with minor admixtures of dicyclopentadiene.

Yield: 150 mg (0.70 mmol, 91 %) of a yellowish oil.

NMR spectral data as above.

endo:exo: >100:1

Chiral HPLC: (Daicel Chiralpak IA-3 (3 µm), 220 nm, 25 °C, 1 % *i*PrOH in heptane, 0.5 ml/min):

t_{R1} (major) = 15.7 min, t_{R2} (minor) = 21.4 min, e.r. = 94.5:5.5, 89 % e.e.

4.3 (-)-*Endo*-5-nitro-6-(*p*-tolyl)bicyclo[2.2.1]hept-2-ene (*endo*-3b)

4.3.1 Isolation at 50 µmol scale and 50 mM concentration with 10 mol% catalyst loading:

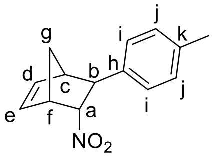

endo-**3b**

Used were 8.22 mg *trans*-4-methyl-β-nitrostyrene (**1b**, 50.4 µmol, 1.0 eq.), 16.6 mg **4**-(*S*)-**8** (5.05 µmol, 10 mol%)**,** 42 µl cyclopentadiene (**2**, 0.50 mmol, 10 eq.) in 1.0 ml dry toluene (50 mM). Eluent (pTLC): 5 % EtOAc in *n*-pentane.

Yield: 10.7 mg (46.5 µmol, 92 %) of a yellowish oil.

R_f (5 % EtOAc in *n*-pentane): 0.4

^{1}H NMR (300 MHz, Chloroform-*d1*): δ = 7.22 (d, *J* = 8.2 Hz, 2H, C[i]-**H**), 7.16 (d, *J* = 8.1 Hz, 2H, C[j]-**H**), 6.61 (dd, *J* = 5.7, 3.2 Hz, 1H, C[d]-**H**), 6.13 (dd, *J* = 5.7, 2.8 Hz, 1H, C[e]-**H**), 5.00 (t, *J* = 4.0 Hz, 1H, C[a]-**H**), 3.66 – 3.56 (m, 1H, C[f]-**H**), 3.41 (t, *J* = 3.4 Hz, 1H, C[b]-**H**), 3.15 (dq, *J* = 3.4, 1.6 Hz, 1H, C[c]-**H**), 2.34 (s, 3H, C[k]-C**H**$_3$), 1.89 (dtt, *J* = 9.3, 1.4, 0.7 Hz, 1H, C[g]-**H**), 1.74 (ddt, *J* = 9.3, 2.7, 1.9 Hz, 1H, C[g]-**H**) ppm.

^{13}C{^{1}H} NMR (75 MHz, Chloroform-*d1*): δ = 140.9 (**C**[d]), 138.5 (**C**[h]), 136.7 (**C**[k]), 133.1 (**C**[e]), 129.7 (**C**[j]), 127.3 (**C**[i]), 92.5 (**C**[a]), 48.8 (**C**[b]), 48.4 (**C**[c]), 48.0 (**C**[f]), 46.2 (**C**[g]), 21.1 (C[k]-**C**H$_3$) ppm.

endo:exo: >100:1

HRMS: (ESI+) (m/z): calc. for $C_{14}H_{16}N_1O_2$ $[M+H]^+$: 230.1181; found: 230.1177.

Chiral HPLC: (YMC CHIRAL ART Amylose-SA S-5 µm, 220 nm, 20 °C, 1 % *i*PrOH in heptane, 1.0 ml/min): t_{R1} (major) = 6.5 min, t_{R2} (minor) = 7.7 min, e.r. = 95:5, 90 % e.e.

4.3.2 Isolation at 100 µmol scale and 50 mM concentration with 5 mol% catalyst loading:

Used were 16.2 mg *trans*-4-methyl-β-nitrostyrene (**1b**, 99.3 µmol, 1.0 eq.), 16.4 mg **4**-(*S*)-**8** (5.00 µmol, 5.0 mol%)**,** 85 µl cyclopentadiene (**2**, 1.0 mmol, 10 eq.) in 2.0 ml dry toluene (50 mM). This reaction was stirred for 67 h instead of 23 h. Eluent (pTLC): 5 % EtOAc in *n*-pentane.

Yield: 20.7 mg (90.3 µmol, 91 %) of a colorless oil.

R_f (5 % EtOAc in *n*-pentane): 0.4

NMR spectral data as above.

endo:exo: >100:1

Chiral HPLC: (YMC CHIRAL ART Amylose-SA S-5 µm, 220 nm, 25 °C, 1 % *i*PrOH in heptane, 1.0 ml/min): t_{R1} (major) = 6.5 min, t_{R2} (minor) = 7.7 min, e.r. = 95:5, 90 % e.e.

$[\alpha]_D^{20}$ = -128° (c = 1.02, $CHCl_3$)

4.4 (-)-*Endo*-5-nitro-6-(4-fluorophenyl)bicyclo[2.2.1]hept-2-ene (*endo*-3c)

4.4.1 Isolation at 50 µmol scale and 50 mM concentration with 10 mol% catalyst loading:

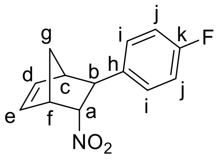

endo-**3c**

Used were 8.36 mg *trans*-4-fluoro-β-nitrostyrene (**1c**, 50.0 µmol, 1.0 eq.), 16.5 mg **4**-(*S*)-**8** (5.05 µmol, 10 mol%)**,** 42 µl cyclopentadiene (**2**, 0.50 mmol, 10 eq.) in 1.0 ml dry toluene (50 mM). Eluent (pTLC): 5 % EtOAc in *n*-pentane.

Yield: 7.35 mg (31.5 µmol, 63 %) of a yellowish oil.

R_f (5 % EtOAc in *n*-pentane): 0.4

NMR spectral data as below.

endo:exo: >100:1

HRMS: (ESI+) (m/z): calc. for $C_{13}H_{13}N_1O_2CF_1$ $[M+H]^+$: 234.0930; found: 234.0927.

Chiral HPLC: (YMC CHIRAL ART Amylose-SA S-5 µm, 220 nm, 25 °C, 1 % *i*PrOH in heptane, 1.0 ml/min): t_{R1} (major) = 7.4 min, t_{R2} (minor) = 10.3 min, e.r. = 93:7, 86 % e.e.

4.4.2 Isolation at 100 µmol scale and 50 mM concentration with 10 mol% catalyst loading:

Used were 16.8 mg *trans*-4-fluoro-β-nitrostyrene (**1c**, 101 µmol, 1.0 eq.), 32.8 mg **4**-(*S*)-**8** (10.0 µmol, 10 mol%)**,** 85 µl cyclopentadiene (**2**, 1.0 mmol, 10 eq.) in 2.0 ml dry toluene (50 mM). Eluent (pTLC): 5 % EtOAc in *n*-pentane.

Yield: 17.6 mg (75.5 µmol, 75 %) of a yellow oil.

R_f (5 % EtOAc in *n*-pentane): 0.4

^{1}H NMR (400 MHz, Chloroform-*d1*): δ = 7.31 (dddd, *J* = 8.3, 5.3, 2.6, 1.5 Hz, 2H, C[i]-**H**), 7.04 (ddt, *J* = 8.7, 6.6, 2.7 Hz, 2H, C[j]-**H**), 6.60 (dd, *J* = 5.7, 3.2 Hz, 1H, C[d]-**H**), 6.15 (dd, *J* = 5.7, 2.8 Hz, 1H, C[e]-**H**), 4.94 (t, *J* = 4.0 Hz, 1H, C[a]-**H**), 3.62 (dhept, *J* = 4.1, 1.5 Hz, 1H, C[f]-**H**), 3.42 (t, *J* = 3.3 Hz, 1H, C[b]-**H**), 3.16 (dq, *J* = 3.4, 1.6 Hz, 1H, C[c]-**H**), 1.87 (dt, *J* = 9.3, 1.5 Hz, 1H, C[g]-**H**), 1.77 (dq, *J* = 9.4, 2.0 Hz, 1H, C[g]-**H**) ppm.

^{19}F NMR (376 MHz, Chloroform-*d1*): δ = -115.89 (ddd, *J* = 13.8, 8.7, 5.2 Hz) ppm.

^{19}F{^{1}H} NMR (376 MHz, Chloroform-*d1*): δ = -115.89 (s) ppm.

^{13}C{^{1}H} NMR (101 MHz, Chloroform-*d1*): δ = 161.9 (d, *J* = 245.9 Hz, **C**[k]), 140.7 (**C**[d]), 137.3 (d, *J* = 3.3 Hz, **C**[h]), 133.4 (**C**[e]), 129.0 (d, *J* = 7.9 Hz, **C**[i]), 115.8 (d, *J* = 21.2 Hz, **C**[j]), 92.6 (**C**[a]), 48.5 (**C**[b]), 48.2 (**C**[c]), 47.9 (**C**[f]), 46.2 (**C**[g]) ppm.

endo:exo: >100:1

Chiral HPLC: (YMC CHIRAL ART Amylose-SA S-5 µm, 220 nm, 25 °C, 1 % *i*PrOH in heptane, 1.0 ml/min): t_{R1} (major) = 7.5 min, t_{R2} (minor) = 10.6 min, e.r. = 93:7, 86 % e.e.

$[\alpha]_D^{20}$ = -121° (c = 1.00, $CHCl_3$)

4.5 (-)-*Endo*-5-nitro-6-(4-chlorophenyl)bicyclo[2.2.1]hept-2-ene (*endo*-3d)

4.5.1 Isolation at 50 µmol scale and 50 mM concentration with 10 mol% catalyst loading:

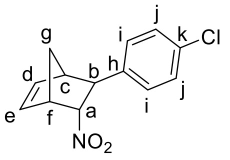

endo-**3d**

Used were 8.36 mg *trans*-4-chloro-β-nitrostyrene (**1d**, 49.9 µmol, 1.0 eq.), 16.5 mg **4**-(*S*)-**8** (5.04 µmol, 10 mol%), 42 µl cyclopentadiene (**2**, 0.50 mmol, 10 eq.) in 1.0 ml dry toluene (50 mM). Eluent (pTLC): 5 % EtOAc in *n*-pentane.

Yield: 9.33 mg (37.4 µmol, 75 %) of a yellowish solid.

R_f (5 % EtOAc in *n*-pentane): 0.3

^{1}H NMR (300 MHz, Chloroform-*d1*): δ = 7.35 – 7.30 (m, 2H, C[i]-**H** or C[j]-**H**), 7.30 – 7.25 (m, 2H, C[i]-**H** or C[j]-**H**), 6.60 (dd, *J* = 5.7, 3.2 Hz, 1H, C[d]-**H**), 6.15 (dd, *J* = 5.7, 2.8 Hz, 1H, C[e]-**H**), 4.93 (t, *J* = 4.0 Hz, 1H, C[a]-**H**), 3.62 (dtt, *J* = 6.3, 2.4, 1.5 Hz, 1H, C[f]-**H**), 3.41 (t, *J* = 3.2 Hz, 1H, C[b]-**H**), 3.16 (dq, *J* = 3.4, 1.6 Hz, 1H, C[c]-**H**), 1.89 – 1.81 (m, 1H, C[g]-**H**), 1.76 (dq, *J* = 9.4, 2.0 Hz, 1H, C[g]-**H**) ppm.

^{13}C{^{1}H} NMR (75 MHz, Chloroform-*d1*): δ = 140.7 (**C**[d]), 140.0 (**C**[h]), 133.5 (**C**[k]), 133.0 (**C**[e]), 129.1 (**C**[i] or **C**[j]), 128.8 (**C**[i] or **C**[j]), 92.4 (**C**[a]), 48.6 (**C**[f]), 48.0 (**C**[c]), 47.8 (**C**[f]), 46.2 (**C**[g]) ppm.

endo:exo: >100:1

HRMS: (ESI+) (m/z): calc. for $C_{13}H_{13}N_1O_2Cl_1$ $[M+H]^+$: 250.0635; found: 250.0630.

Chiral HPLC: (YMC CHIRAL ART Amylose-SA S-5 µm, 220 nm, 25 °C, 1 % *i*PrOH in heptane, 1.0 ml/min): t_{R1} (major) = 8.0 min, t_{R2} (minor) = 10.9 min, e.r. = 93.5:6.5, 86 % e.e.

4.5.2 Isolation at 100 µmol scale and 50 mM concentration with 10 mol% catalyst loading:

Used were 18.3 mg *trans*-4-chloro-β-nitrostyrene (**1d**, 99.7 µmol, 1.0 eq.), 33.2 mg **4**-(*S*)-**8** (10.1 µmol, 10 mol%), 85 µl cyclopentadiene (**2**, 1.0 mmol, 10 eq.) in 2.0 ml dry toluene (50 mM). Eluent (pTLC): 5 % EtOAc in *n*-pentane. Due to separation issues two pTLC steps were performed.

Yield: 24.5 mg (98.1 µmol, 98 %) of a yellowish solid.

R_f (5 % EtOAc in *n*-pentane): 0.3

NMR spectral data as above

endo:exo: >100:1

Chiral HPLC: (YMC CHIRAL ART Amylose-SA S-5 µm, 220 nm, 25 °C, 1 % *i*PrOH in heptane, 1.0 ml/min): t_{R1} (major) = 8.1 min, t_{R2} (minor) = 11.1 min, e.r. = 94.5:5.5, 89 % e.e.

$[\alpha]_D^{20}$ = -107° (c = 1.00, $CHCl_3$)

4.6 (-)-*Endo*-5-nitro-6-(4-methoxyphenyl)bicyclo[2.2.1]hept-2-ene (*endo*-3e)

4.6.1 Isolation at 50 µmol scale and 50 mM concentration with 10 mol% catalyst loading:

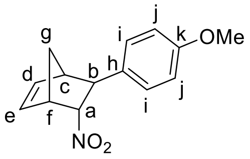

endo-**3e**

Used were 8.99 mg *trans*-4-methoxy-β-nitrostyrene (**1e**, 50.2 µmol, 1.0 eq.), 16.3 mg **4**-(*S*)-**8** (4.97 µmol, 10 mol%)**,** 42 µl cyclopentadiene (**2**, 0.50 mmol, 10 eq.) in 1.0 ml dry toluene (50 mM). Eluent (pTLC): 5 % EtOAc in *n*-pentane.

Yield: 11.5 mg (46.8 µmol, 93 %) of a yellowish oil.

R_f (5 % EtOAc in *n*-pentane): 0.4

^{1}H NMR (300 MHz, Chloroform-*d1*): δ = 7.30 – 7.22 (m, 2H, C[i]-**H**), 6.89 (dt, *J* = 8.8, 3.1, 2.2 Hz, 2H, C[j]-**H**), 6.60 (dd, *J* = 5.7, 3.2 Hz, 1H, C[d]-**H**), 6.13 (dd, *J* = 5.7, 2.8 Hz, 1H, C[e]-**H**), 4.96 (t, *J* = 4.0 Hz, 1H, C[a]-**H**), 3.81 (s, 3H, C[k]-OC**H₃**), 3.60 (ddt, *J* = 4.1, 2.6, 1.4 Hz, 1H, C[f]-**H**), 3.38 (t, *J* = 3.4 Hz, 1H, C[b]-**H**), 3.13 (dq, *J* = 3.4, 1.6 Hz, 1H, C[c]-**H**), 1.89 (dtt, *J* = 9.3, 1.4, 0.7 Hz, 1H, C[g]-**H**), 1.74 (dq, *J* = 9.3, 2.5, 1.9, 1.9 Hz, 1H, C[g]-**H**) ppm.

^{13}C{^{1}H} NMR (75 MHz, Chloroform-*d1*): δ = 158.6 (**C**[k]), 140.9 (**C**[d]), 133.5 (**C**[h]), 133.2 (**C**[e]), 128.5 (**C**[i]), 114.4 (**C**[j]), 92.6 (**C**[a]), 55.5 (C[k]-O**C**H_3), 48.5 (**C**[b]), 48.4 (**C**[c]), 47.9 (**C**[f]), 46.2(**C**[g]) ppm.

endo:exo: >100:1

HRMS: (ESI+) (m/z): calc. for $C_{14}H_{16}N_1O_3$ [M+H]$^+$: 246.1130; found: 246.1127.

Chiral HPLC: (YMC CHIRAL ART Amylose-SA S-5 µm, 220 nm, 20 °C, 1 % *i*PrOH in heptane, 1.0 ml/min): t_{R1} (major) = 10.9 min, t_{R2} (minor) = 13.8 min, e.r. = 97:3, 94 % e.e.

4.6.2 Isolation at 100 µmol scale and 50 mM concentration with 5 mol% catalyst loading:

Used were 17.8 mg *trans*-4-methoxy-β-nitrostyrene (**1e**, 99.3 µmol, 1.0 eq.), 16.2 mg **4**-(*S*)-**8** (4.95 µmol, 5.0 mol%)**,** 85 µl cyclopentadiene (**2**, 1.0 mmol, 10 eq.) in 2.0 ml dry toluene (50 mM). This reaction was stirred for 67 h instead of 23 h. Eluent (pTLC): 5 % EtOAc in *n*-pentane.

Yield: 23.1 mg (94.2 µmol, 95 %) of a slightly yellowish solid.

R_f (5 % EtOAc in *n*-pentane): 0.3

NMR spectral data as above

endo:exo: >100:1

Chiral HPLC: (YMC CHIRAL ART Amylose-SA S-5 µm, 220 nm, 25 °C, 1 % *i*PrOH in heptane, 1.0 ml/min): t_{R1} (major) = 11.0 min, t_{R2} (minor) = 14.3 min, e.r. = 96.5:3.5, 93 % e.e.

$[\alpha]_D^{20}$ = -128° (c = 1.02, $CHCl_3$)

4.7 (-)-*Endo*-5-nitro-6-(2-methoxyphenyl)bicyclo[2.2.1]hept-2-ene (*endo*-3f)

4.7.1 Isolation at 50 µmol scale and 50 mM concentration with 10 mol% catalyst loading:

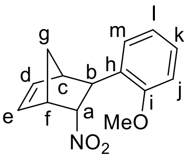

endo-**3f**

Used were 9.00 mg *trans*-2-methoxy-β-nitrostyrene (**1f**, 50.2 µmol, 1.0 eq.), 16.3 mg **4**-(*S*)-**8** (4.97 µmol, 10 mol%), 42 µl cyclopentadiene (**2**, 0.50 mmol, 10 eq.) in 1.0 ml dry toluene (50 mM). Eluent (pTLC): 5 % EtOAc in *n*-pentane.

Yield: 11.8 mg (48.0 µmol, 96 %) of a yellowish solid.

R_f (5 % EtOAc in *n*-pentane): 0.3

^{1}H NMR (300 MHz, Chloroform-*d1*): δ = 7.29 – 7.17 (m, 2H, C[k]-**H** & C[m]-**H**), 6.95 (td, *J* = 7.5, 1.2 Hz, 1H, C[l]-**H**), 6.86 (dd, *J* = 8.2, 1.2 Hz, 1H, C[j]-**H**), 6.62 (dd, *J* = 5.7, 3.3 Hz, 1H, C[d]-**H**), 6.04 (dd, *J* = 5.7, 2.8 Hz, 1H, C[e]-**H**), 4.85 (t, *J* = 4.0 Hz, 1H, C[a]-**H**), 3.73 (s, 3H, C[i]-OC**H$_3$**), 3.53 – 3.43 (m, 2H, C[b]-**H**, C[f]-**H**), 3.34 (dt, *J* = 3.4, 1.7 Hz, 1H, C[c]-**H**), 1.80 (dddd, *J* = 9.1, 2.1, 1.4, 0.6 Hz, 1H, C[g]-**H**), 1.72 (ddt, *J* = 9.1, 2.7, 1.8 Hz, 1H, C[g]-**H**) ppm.

^{13}C{^{1}H} NMR (75 MHz, Chloroform-*d1*): δ = 157.8 (**C**[i]), 139.9 (**C**[d]), 132.3 (**C**[e]), 130.3 (**C**[h]), 128.1 (**C**[k]), 125.8 (**C**[m]), 120.6 (**C**[l]), 110.3 (**C**[j]), 91.5 (**C**[a]), 55.1 (C[i]-O**C**H$_3$), 49.3 (**C**[b] or **C**[f]), 46.9 (**C**[g]), 45.7 (**C**[c]), 44.1 (**C**[b] or **C**[f]) ppm.

endo:exo: >100:1

HRMS: (ESI+) (m/z): calc. for $C_{14}H_{16}N_1O_3$ [M+H]$^+$: 246.1130; found: 246.1127.

Chiral HPLC: (YMC CHIRAL ART Amylose-SA S-5 µm, 220 nm, 20 °C, 1 % *i*PrOH in heptane, 1.0 ml/min): t_{R1} (minor) = 7.1 min, t_{R2} (major) = 8.1 min, e.r. = 97:3, 94 % e.e.

4.7.2 Isolation at 100 µmol scale and 50 mM concentration with 5 mol% catalyst loading:

Used were 17.8 mg *trans*-2-methoxy-β-nitrostyrene (**1f**, 99.3 µmol, 1.0 eq.), 16.4 mg **4**-(*S*)-**8** (5.01 µmol, 5.0 mol%), 85 µl cyclopentadiene (**2**, 1.0 mmol, 10 eq.) in 2.0 ml dry toluene (50 mM). This reaction was stirred for 67 h instead of 23 h. Eluent (pTLC): 5 % EtOAc in *n*-pentane.

Yield: 23.5 mg (95.8 µmol, 96 %) of a yellowish solid.

R_f (5 % EtOAc in *n*-pentane): 0.3

NMR spectral data as above

endo:exo: >100:1

Chiral HPLC: (YMC CHIRAL ART Amylose-SA S-5 µm, 220 nm, 25 °C, 1 % *i*PrOH in heptane, 1.0 ml/min): t_{R1} (minor) = 7.0 min, t_{R2} (major) = 7.9 min, e.r. = 97:3, 94 % e.e.

$[\alpha]_D^{20}$ = -24.6° (c = 1.01, $CHCl_3$)

4.8 (-)-*Endo*-5-nitro-6-(2,4-dimethoxyphenyl)bicyclo[2.2.1]hept-2-ene (*endo*-3g)

4.8.1 Isolation at 50 µmol scale and 50 mM concentration with 10 mol% catalyst loading:

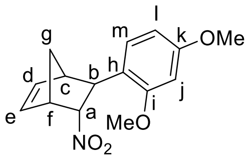

endo-**3g**

Used were 10.4 mg *trans*-2,4-dimethoxy-β-nitrostyrene (**1g**, 49.9 µmol, 1.0 eq.), 16.5 mg **4**-(*S*)-**8** (5.04 µmol, 10 mol%)**,** 42 µl cyclopentadiene (**2**, 0.50 mmol, 10 eq.) in 1.0 ml dry toluene (50 mM). Eluent (pTLC): 10 % EtOAc in *n*-pentane.

Yield: 12.3 mg (44.6 µmol, 89 %) of a yellowish solid.

R_f (10 % EtOAc in *n*-pentane): 0.3

^{1}H NMR (300 MHz, Chloroform-*d1*): δ = 7.08 (dq, *J* = 8.9, 1.3 Hz, 1H, C[m]-**H**), 6.60 (dd, *J* = 5.7, 3.3 Hz, 1H, C[d]-**H**), 6.50 – 6.42 (m, 2H, C[j]-**H** & C[l]-**H**), 6.03 (dd, *J* = 5.7, 2.8 Hz, 1H, C[e]-**H**), 4.82 (t, *J* = 4.0 Hz, 1H, C[a]-**H**), 3.80 (s, 3H, C[i]-OC**H$_3$** or C[k]-OC**H$_3$**), 3.70 (s, 3H, C[i]-OC**H$_3$** or C[k]-OC**H$_3$**), 3.48 (tt, *J* = 2.6, 1.0 Hz, 1H, C[f]-**H**), 3.40 (td, *J* = 3.0, 1.5 Hz, 1H, C[b]-**H**), 3.29 (dt, *J* = 3.5, 1.7 Hz, 1H, C[c]-**H**), 1.78 (dp, *J* = 9.1, 0.7 Hz, 1H, C[g]-**H**), 1.70 (ddt, *J* = 9.1, 2.6, 1.8 Hz, 1H, C[g]-**H**) ppm.

^{13}C{^{1}H} NMR (75 MHz, Chloroform-*d1*): δ = 159.9 (**C**[i] or **C**[k]), 158.8 (**C**[i] or **C**[k]), 139.9 (**C**[d]), 132.2 (**C**[e]), 126.2 (**C**[m]), 122.8 (**C**[h]), 103.9 (**C**[l]), 98.6 (**C**[j]), 91.7 (**C**[a]), 55.6 (C[i]-O**C**H$_3$ or C[k]-O**C**H$_3$), 55.2 (C[i]-O**C**H$_3$ or C[k]-O**C**H$_3$), 49.3 (**C**[f]), 46.9 (**C**[g]), 45.8 (**C**[c]), 43.7 (**C**[b]) ppm.

endo:exo: >100:1

HRMS: (ESI+) (m/z): calc. for $C_{15}H_{18}N_1O_4$ [M+H]$^+$: 276.1236; found: 276.1235.

Chiral HPLC: (YMC CHIRAL ART Amylose-SA S-5 µm, 220 nm, 25 °C, 1 % *i*PrOH in heptane, 1.0 ml/min): t_{R1} (minor) = 10.4 min, t_{R2} (major) = 12.9 min, e.r. = 98.5:1.5, 97 % e.e.

4.8.2 Isolation at 100 µmol scale and 50 mM concentration with 5 mol% catalyst loading:

Used were 21.1 mg *trans*-2,4-dimethoxy-β-nitrostyrene (**1g**, 101 µmol, 1.0 eq.), 16.4 mg **4**-(*S*)-**8** (5.01 µmol, 5.0 mol%)**,** 85 µl cyclopentadiene (**2**, 1.00 mmol, 10 eq.) in 2.0 ml dry toluene (50 mM). This reaction was stirred for 67 h instead of 23 h. Eluent (pTLC): 10 % EtOAc in *n*-pentane.

Yield: 23.8 mg (86.5 µmol, 86 %) of a yellowish solid.

R_f (10 % EtOAc in *n*-pentane): 0.3

NMR spectral data as above

endo:exo: >100:1

Chiral HPLC: (YMC CHIRAL ART Amylose-SA S-5 µm, 220 nm, 25 °C, 1 % *i*PrOH in heptane, 1.0 ml/min): t_{R1} (minor) = 10.4 min, t_{R2} (major) = 12.8 min, e.r. = 98:2, 96 % e.e.

$[\alpha]_D^{20}$ = -44.3° (c = 0.99, $CHCl_3$)

4.9 (-)-*Endo*-5-nitro-6-(3,4-dimethoxyphenyl)bicyclo[2.2.1]hept-2-ene (*endo*-3h)

4.9.1 Isolation at 50 µmol scale and 50 mM concentration with 10 mol% catalyst loading:

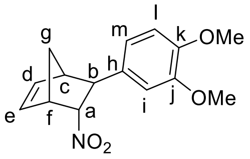

endo-**3h**

Used were 10.5 mg *trans*-3,4-dimethoxy-β-nitrostyrene (**1h**, 50.1 µmol, 1.0 eq.), 16.5 mg **4**-(*S*)-**8** (5.03 µmol, 10 mol%)**,** 42 µl cyclopentadiene (**2**, 0.50 mmol, 10 eq.) in 1.0 ml dry toluene (50 mM). Eluent (pTLC): 20 % EtOAc in *n*-pentane. Due to separation issues several pTLC steps were performed.

Yield: 9.63 mg (35.0 µmol, 70 %) of a yellowish solid.

R_f (20 % EtOAc in *n*-pentane): 0.3

^{1}H NMR (300 MHz, Chloroform-*d1*): δ = 6.90 – 6.83 (m, 3H, C[i]-**H**, C[l]-**H** & C[m]-**H**), 6.60 (dd, *J* = 5.7, 3.2 Hz, 1H, C[d]-**H**), 6.14 (dd, *J* = 5.7, 2.7 Hz, 1H, C[e]-**H**), 4.97 (t, *J* = 4.0 Hz, 1H, C[a]-**H**), 3.88 (s, 3H, C[j]-OC**H₃** or C[k]-OC**H₃**), 3.87 (s, 3H, C[j]-OC**H₃** or C[k]-OC**H₃**), 3.63 – 3.58 (m, *J* = 1.2 Hz, 1H, C[f]-**H**), 3.37 (dd, *J* = 4.0, 2.6 Hz, 1H, C[b]-**H**), 3.15 (dt, *J* = 3.4, 1.6 Hz, 1H, C[c]-**H**), 1.90 (dddd, *J* = 9.3, 2.1, 1.4, 0.6 Hz, 1H, C[g]-**H**), 1.75 (dq, *J* = 9.3, 2.1 Hz, 1H, C[g]-**H**) ppm.

^{13}C{^{1}H} NMR (75 MHz, Chloroform-*d1*): δ = 149.3 (**C**[j] or **C**[k]), 148.2 (**C**[j] or **C**[k]), 140.8 (**C**[d]), 134.1 (**C**[h]), 133.3 (**C**[e]), 118.6 (**C**[i]), 111.4 (**C**[m] or **C**[l]), 111.4 (**C**[m] or **C**[l]), 92.6 (**C**[a]), 56.1 (C[j]-O**C**H_3 & C[k]-O**C**H_3), 48.8 (**C**[b]), 48.3 (**C**[c]), 47.8 (**C**[f]), 46.3 (**C**[g]) ppm.

endo:exo: >100:1

HRMS: (ESI+) (m/z): calc. for $C_{15}H_{18}N_1O_4$ $[M+H]^+$: 276.1236; found: 276.1235.

Chiral HPLC: (YMC CHIRAL ART Amylose-SA S-5 µm, 220 nm, 25 °C, 3 % *i*PrOH in heptane, 1.0 ml/min): t_{R1} (minor) = 12.3 min, t_{R2} (major) = 14.4 min, e.r. = 97.5:2.5, 95 % e.e.

4.9.2 Isolation at 100 µmol scale and 50 mM concentration with 5 mol% catalyst loading:

Used were 20.8 mg *trans*-3,4-dimethoxy-β-nitrostyrene (**1h**, 99.4 µmol, 1.0 eq.), 16.5 mg **4**-(*S*)-**8** (5.04 µmol, 5.1 mol%)**,** 85 µl cyclopentadiene (**2**, 1.0 mmol, 10 eq.) in 2.0 ml dry toluene (50 mM). This reaction was stirred for 67 h instead of 23 h. Eluent (pTLC): 20 % EtOAc in *n*-pentane. Due to separation issues several pTLC steps were performed.

Yield: 18.1 mg (65.7 µmol, 66 %) of a yellowish solid.

R_f (20 % EtOAc in *n*-pentane): 0.3

NMR spectral data as above

endo:exo: >100:1

Chiral HPLC: (YMC CHIRAL ART Amylose-SA S-5 µm, 220 nm, 25 °C, 3 % *i*PrOH in heptane, 1.0 ml/min): t_{R1} (minor) = 12.2 min, t_{R2} (major) = 14.2 min, e.r. = 97.5:2.5, 95 % e.e.

$[\alpha]_D^{20}$ = -115° (c = 0.99, $CHCl_3$)

4.10 (-)-*Endo*-5-cyclohexyl-6-nitrobicyclo[2.2.1]hept-2-ene (*endo*-3i)

4.10.1 Isolation at 100 µmol scale and 50 mM concentration with 10 mol% catalyst loading:

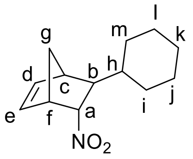

endo-**3i**

Used were 15.5 mg cyclohexyl-β-nitrostyrene (**1i**, 100 µmol, 1.0 eq), 32.0 mg **4**-(*S*)-**8** (9.7 µmol, 9.8 mol%), 85 µl cyclopentadiene (**2**, 1.0 mmol, 10 eq.) in 2.0 ml dry toluene (50 mM). This reaction was stirred for 65 h instead of 23 h. Eluent (pTLC): 5 % EtOAc in *n*-pentane.

Yield: 1.8 mg (8.3 µmol, 8 %) of a clear viscous liquid.

R_f (5 % EtOAc in *n*-pentane): 0.85

^{1}H NMR (501 MHz, Chloroform-*d1*): δ = 6.47 (dd, *J* = 5.8, 3.3 Hz, 1H, C[d]-**H**), 6.32 – 6.28 (m, 0.1H, C[d]-**H**-*exo*), 6.17 – 6.13 (m, 0.1H, C[e]-**H**-*exo*), 5.98 (dd, *J* = 5.7, 2.8 Hz, 1H, C[e]-**H**), 4.70 (t, *J* = 3.9 Hz, 1H, C[a]-**H**), 4.60 – 4.56 (m, 0.14H, C[a]-**H**-*exo*), 3.51 – 3.45 (m, 1H, C[f]-**H**), 3.31 – 3.28 (m, 0.12H, C[f]-**H**-*exo*), 3.05 – 3.01 (m, 0.1H, C[b]-**H**-*exo*), 2.97 – 2.91 (m, 1H, C[b]-**H**), 1.97 – 1.84 (m, 2H, C[c]-**H** + C[g]-**H**), 1.84 – 1.49 (m, 5.6H, C[g]-**H** + C[i]-**H** + C[m]-**H** + *exo*), 1.35 – 1.16 (m, 6.6H, C[j]-**H** + C[l]-**H** + C[k]-**H** + *exo*), 1.14 – 0.97 (m, 1.5H, C[h]-**H** + *exo*) ppm.

^{13}C{^{1}H} NMR (126 MHz, Chloroform-*d1*): δ = 140.6 (**C**[d]), 131.8 (**C**[e]), 89.9 (**C**[a]), 51.2 (**C**[f]), 48.0 (**C**[b]), 45.7 (**C**[c]), 44.3 (**C**[g]), 41.3 (**C**[h]), 32.2 (**C**[m] or **C**[i]), 32.0 (**C**[m] or **C**[i]), 29.7 (**C**[j] or **C**[l]), 26.3 (**C**[j] or **C**[l]), 26.1 (**C**[k]) ppm.

endo:exo: 9:1

HRMS: (ESI+) (m/z): calc. for $C_{13}H_{19}N_1O_2$ $[M+Na]^+$: 244.1308; found: 244.1309.

Chiral GC: (BGB-176 30m x 0.25mm, injection temperature: 220 °C, 80 °C, 0.5 bar He):

t_{R1}-*exo* (minor) = 36.1 min, t_{R2}-*exo* (minor) = 37.1 min, t_{R1}-*endo* (major) = 39.5 min, t_{R1}-*endo* (major) = 41.1 min; e.r. *exo* (minor) = 5:5, 0 % e.e., e.r. *endo* (major) = 79:21, 58 % e.e.

$[\alpha]_D^{20}$ = -22° (c = 0.56, $CHCl_3$)

4.11 Limitations

Investigations on limitations of the scope were performed in analytical scale (12.5 µmol) following the "Screening Procedure for Diels-Alder Reaction" (Section 3.2) under the optimized conditions (50 mM, r.t., 10 mol% catalyst **4-(*S*)-8**). The reaction did not take place when applying dienes 1,3-cyclohexadiene (**SI-18**) and 2,3-dimethyl-1,3-butadiene (**SI-19**) instead of cyclopentadiene (**2**) (Table S6, entries 1 & 2). Similarly, no product formation was observed when applying α-methyl substituted nitrostyrene **SI-20**, electron-rich nitrostyrene **SI-21** or methyl cinnamate (**SI-22**) as dienophiles (Table S6, entries 3 – 5). With aromatic nitroolefins **SI-23** & **SI-24** the reaction worked, but with e.e. < 85 % and therefore these were not further pursued for the isolation experiments (Table S6, entries 6 & 7). For chlorinated nitrostyrene **SI-22**, a high thermal reactivity was noticed which may be the reason for the low enantioselectivity.

Table S6: Additional results on the scope of this Diels-Alder reaction. Changes to procedure from section 3.2 are given or described above. (n.d. = no product detectable)

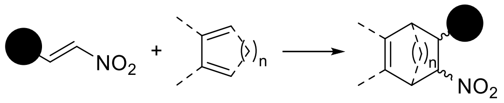

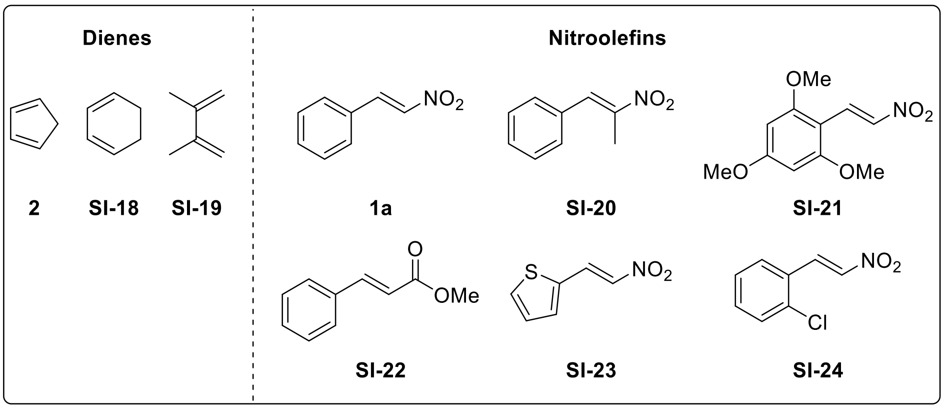

Entry	Catalyst (mol%)	Diene	Nitroolefin	Yield (%)	e.r.[c]
1	**4**-(*S*)-**8** (10)	**SI-18**	**1a**	n.d.	-
2	**4**-(*S*)-**8** (10)	**SI-19**	**1a**	n.d.	-
3	**4**-(*S*)-**8** (10)	**2**	**SI-20**	n.d.	-
4	**4**-(*S*)-**8** (10)	**2**	**SI-21**	n.d.	-
5	**4**-(*S*)-**8** (10)	**2**	**SI-22**	n.d.	-
6	**4**-(*S*)-**8** (10)	**2**	**SI-23**	88 %	91:9
7	**4**-(*S*)-**8** (10)	**2**	**SI-24**	> 95 %	85:15

4.11.1 HPLC conditions

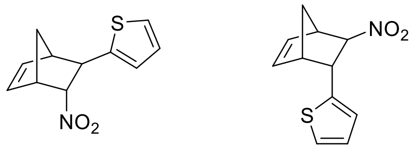

endo-**SI-25** *exo*-**SI-25**

Chiral HPLC: (Dr. Maisch ReproSil Chiral-AM 5 µm, 220 nm, 25 °C, 3 % *i*PrOH in heptane, 1.0 ml/min): (overlap of endo and exo, but only in racemic reference sample due to lower diastereoselectivity)

endo-**SI-25 (major)**: t_{RA1} = 5.8 min, t_{RA2} = 9.0 min.

exo-**SI-25 (minor)**: t_{RB1} = 5.6 min, t_{RB2} = 6.2 min.

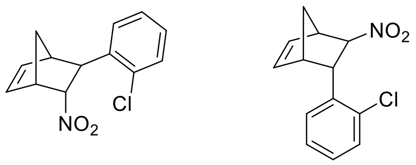

endo-**SI-26** *exo*-**SI-26**

Chiral HPLC: (YMC CHIRAL ART Amylose-SA S-5 µm, 220 nm, 25 °C, 1 % *i*PrOH in heptane, 1.0 ml/min):

endo-**SI-26 (major)**: t_{RA1} = 8.0 min, t_{RA2} = 10.0 min.

exo-**SI-26 (minor)**: t_{RB1} = 5.9 min, t_{RB2} = 6.8 min.

5. Synthesis of (-)-fencamfamine (*endo*-12)

The synthesis of (-)-fencamfamine was performed adopting the procedures of Novakov *et al.*[20] and the patent of *Praxis Biosciences*.[21] The racemic reference compound was synthesized as described further starting with corresponding thermal Diels-Alder reaction product.

5.1 *Endo*-phenylbicyclo[2.2.1]heptan-2-amine (*endo*-10)

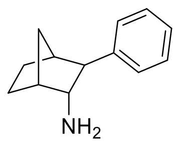

endo-**10**

In a flame-dried 50 ml Schlenk flask equipped with magnetic stirrer under argon atmosphere the enantioenriched (94.5:5.5 e.r.) *endo*-**3a** (150 mg, 0.70 mmol, 1 eq.) was dissolved in 3 ml of THF. Then a solution of NaOH (162 mg, 4.05 mmol, 5.8 eq.) in 5 ml of water was added to the flask. Powdered nickel-aluminum alloy (1025 mg, 17 eq.) was added in small portions to the resulting mixture with intense mixing. The rate of addition of the alloy was regulated to keep the mixture boiling moderately.

After addition of the alloy, the reaction mixture was refluxed in an oil bath (75 °C) for 2 h, filtered hot, and the residue was washed with THF (3x5 ml). Concentrated H_2SO_4 was added to the cooled filtrate (until pH 3) and THF was evaporated in a water bath. The obtained solution was extracted with Et_2O (3x5 ml), and the obtained aqueous phase was adjusted to pH 12 by addition of a 2M NaOH solution. The resulting mixture was extracted with toluene (3x5 ml) to afford the crude amine solution, which was dried over solid NaOH, filtered, and evaporated *in vacuo*. The obtained crude amine product *endo*-**10** (140 mg) was used in the next step without further purification.

5.2 *Endo*-phenylbicyclo[2.2.1]heptan-2-amine (*endo*-11)

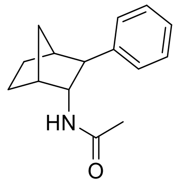

endo-**11**

In a flame-dried 25 ml flask the crude amine *endo*-**10** obtained in the previous step (140 mg) was dissolved in 2 ml DCM and to the resulting solution triethylamine (0.210 ml, 1.51 mmol, 2.2 eq.) was added. The reaction mixture was stirred at room temperature for 10 min, cooled down using an ice bath and acetyl chloride (0.064 ml, 0.90 mmol, 1.3 eq.) was added dropwise. After stirring the reaction mixture for 2 h, it was quenched with water (2 ml) and extracted with DCM (3x3 ml). The combined organic extracts were washed 1N HCl (5 ml), sat. $NaHCO_3$ (5 ml) and brine (10 ml), dried over anhydrous Na_2SO_4 and concentrated *in vacuo* to afford yellowish wax-like product. The obtained product was triturated with minimal amount Et_2O resulting in formation of an off-white solid which was dried in high vacuum (133 mg) and used in the next step without further purification.

5.3 (-)-*Endo*-N-ethyl-3-phenylbicyclo[2.2.1]heptan-2-amine (12)

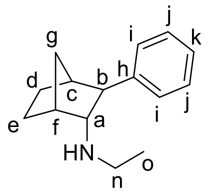

endo-**12**

In a flame-dried 25 ml flask lithium aluminum hydride (44 mg, 1.2 mmol, 1.7 eq.) was suspended in dry THF (1 ml). The crude amide *endo*-**11** obtained in the previous step (133 mg) was dissolved in dry THF (1 ml) and added dropwise at 0 °C to the suspension. After the addition was complete, the reaction mixture was refluxed for 16 h under Argon atmosphere. Then the reaction mixture was cooled down and added dropwise to an ice-cold solution of 4N NaOH (2 ml) with stirring. After stirring for 15 min, it was then filtered through Celite. The filtrate was extracted with EtOAc (3x2 ml) and washed with brine (2x2 ml). The organic layer was dried over anhydrous sodium sulfate and concentrated under reduced pressure to obtain crude product, as a pale-yellow liquid.

To a solution of crude product in diethyl ether (1 ml) was added 2.0 M ethereal HCl (2 ml) at 0 °C. After 1 h, the off-white precipitate was formed and collected, washed with excess of diethyl ether and dried in high vacuum. The obtained solid was dissolved in EtOAc (2 ml) and basified with 2N NaOH solution. The separated organic layer was treated with brine (2x2 ml), dried over anhydrous sodium sulfate and concentrated under reduced pressure to obtain the amine.

Yield: 89 mg (0.45 mmol, 64 % over 3 steps) of a pale-yellow liquid.

R_f (1 % TEA in DCM:MeOH 10:1): 0.2

^{1}H NMR (501 MHz, Chloroform-*d1*): δ = 7.29 – 7.22 (m, 4H, C[i]-**H**, C[j]-**H**), 7.17 – 7.12 (m, 1H, C[k]-**H**), 3.11 – 3.04 (m, 1H, C[a]-**H**), 2.63 – 2.44 (m, 2H, C[n]-**H**), 2.41 – 2.37 (m, 1H, C[f]-**H**), 2.23 – 2.19 (m, 1H, C[c]-**H**), 2.11 (dd, *J* = 5.5, 2.1 Hz, 1H, C[b]-**H**), 1.77 – 1.69 (m, 2H, C[g]-**H**), 1.66 – 1.55 (m, 2H, C[d]-**H** + C[e]-**H**), 1.46 (brs, 1H, NH) 1.40 – 1.30 (m, 2H, C[d]-**H** + C[e]-**H**), 1.03 (t, *J* = 7.2 Hz, 3H, C[o]-**H**) ppm.

^{13}C{^{1}H} NMR (126 MHz, Chloroform-*d1*): δ = 140.0 (**C**[h]), 128.3 (**C**[j]), 127.1 (**C**[i]), 126.0 (**C**[k]), 67.5 (**C**[a]), 56.4 (**C**[b]), 44.7 (**C**[n]), 42.9 (**C**[f]), 39.8 (**C**[g]), 36.3 (**C**[d]), 31.1 (**C**[c]), 20.7 (**C**[e]), 15.3 (**C**[o]).

endo:exo: >100:1

HRMS: (ESI+) (m/z): calc. for $C_{15}H_{22}N_1$ $[M+H]^+$: 216.1747; found: 216.1748

Chiral HPLC: (Daicel Chiralpak AD, 10 µm, 220 nm, 25 °C, 10% NH_4HCO_3 (20 mM aq.) in methanol, 1.0 ml/min): t_{R1} (minor) = 14.9 min, t_{R2} (major) = 16.4 min; e.r. = 94.5:5.5, 89 % e.e.

$[\alpha]_D^{20}$ = -59° (c = 0.61, $CHCl_3$)

6. Absolute Configuration Determination by VCD spectroscopy

6.1 Experimental details

The IR and VCD spectrum were recorded on a Bruker Vertex 70/PMA 50 VCD spectrometer at 4 cm^{-1} spectral resolution by accumulating 32 scans for the IR and ~60000 scans (14 h accumulation time) for VCD. The samples were dissolved in $CDCl_3$ at the concentration given in the respective captions and measured using a BaF_2 IR cell with 100 µm optical path length. Baseline correction of the VCD spectra was done by subtraction of the spectra of the solvent recorded under identical conditions.

6.2 Computational details

Deriving the absolute configuration from the experimental spectra requires the computation of IR and VCD spectra. Therefore, a conformational sampling was carried out based on a systematic search algorithm on force-field level (MMFF).[22,23] All so-obtained conformers were subjected to further geometry optimizations at B3LYP/6-311+G(2d,p)/IEFPCM($CHCl_3$) level of theory using Gaussian 16.[24] For the final comparison with the experiment, the IR and VCD spectra were simulated from the single-conformer spectra using the ΔE_{ZPC}-based Boltzmann weights and by assigning a Lorentzian band shape with half-width at half-height of 6 cm^{-1} to the computed dipole and rotational strength. To account for effects not captured in the harmonic approximation accounted for in the spectra calculations, the frequency axis was uniformly scaled by 0.98.

6.3 Analysis of the spectra

As the relative configuration of *endo*-**3a** was determined as *endo* from NMR spectroscopy, VCD spectra were recorded to determine the absolute configuration. Spectra calculations were carried out on the (1*R*,4*S*,5*R*,6*S*)-enantiomer based on two conformers, the lowest energy conformer shown in Figure S6 and a 0.2 kcal/mol less stable structure with a slightly rotated phenyl ring. Comparison of the experimental and computed spectra revealed a good match, that allows for the unambiguous assignment of the proposed configuration to *endo*-**3a**. It shall be noted that the two strong bands in the IR spectrum, of which one gives a very strong VCD feature (1379 cm^{-1}), arise from the N-O stretching vibration. Like in the present case, calculations often tend to predict this mode to be more intense than experimentally observed. Yet, despite the intensity difference in VCD, the negative sign matches with the experimental signature.

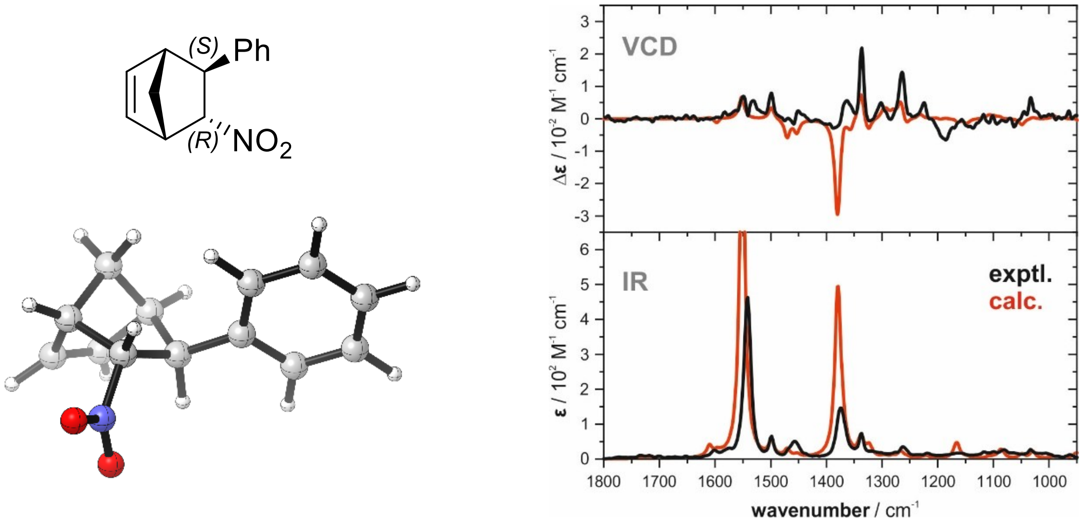

Figure S6: Lowest energy conformation of (1*R*,4*S*,5*R*,6*S*)-*endo*-**3a** (left) and comparison of the experimental (0.15 M, CDCl3, 100 µm) and computed IR and VCD spectra of (1*R*,4*S*,5*R*,6*S*)-*endo*-**3a** (right). The graphic was produced using CYLview20.[25]

6.4 Cartesian coordinates of c1 and c2

C1 (0.0 kcal/mol)

C	0.35752900	1.96426000	-1.17390600
C	2.31986100	2.37110200	-0.13350300
C	1.84291100	1.57225000	-1.36864500
H	2.27112400	1.90926400	-2.31642300
H	1.99642200	0.49616000	-1.26238600
C	0.53571900	3.52593400	-1.30465400
H	0.61334100	3.79543200	-2.35554500
C	1.81449100	3.84116700	-0.48636600
H	1.52327300	4.33378400	0.44110200
H	-0.37461800	1.56215200	-1.87273900
H	3.37779100	2.33110000	0.12201500
C	2.84322200	4.72393400	-1.16797200
C	3.52059900	5.67557500	-0.39111300
C	3.18418800	4.62326500	-2.52462400
C	4.50745200	6.49291600	-0.94201400
H	3.26741400	5.77930300	0.66038200
C	4.16940700	5.44046700	-3.08150900
H	2.68234300	3.90986300	-3.16962000
C	4.83715500	6.37789000	-2.29300200
H	5.01216600	7.22261500	-0.31660000
H	4.41098800	5.34399800	-4.13540800
H	5.60109100	7.01440800	-2.72765400
N	-0.70406000	4.25474300	-0.85373100
O	-1.58805800	4.38892700	-1.69601400
O	-0.79763100	4.63509600	0.30479300
H	-0.76926600	1.35562000	0.75387300
H	1.54577400	1.81346900	1.97466000
C	0.17399600	1.62790200	0.29733300
C	1.34450500	1.85631500	0.91084900

C2 (0.2 kcal/mol)

C	0.19388900	2.15403800	-1.20161300
C	2.30504500	2.16007500	-0.39716900
C	1.57893000	1.64508200	-1.66658400
H	1.91685800	2.10184800	-2.60107600
H	1.60459900	0.55699500	-1.75758200
C	0.56119900	3.68338300	-1.10826300
H	0.57042600	4.11738300	-2.10640600
C	1.96337500	3.70375000	-0.42451800
H	1.83869100	4.05798200	0.59963200
H	-0.66312600	1.96453900	-1.84654500
H	3.36911800	1.94931100	-0.29832300
C	2.96013100	4.62382300	-1.11005900
C	2.98877800	5.97794500	-0.74234200
C	3.86098100	4.18835000	-2.09019800
C	3.87428000	6.87148700	-1.34375300
H	2.31287300	6.33548400	0.02964900
C	4.75361900	5.07893400	-2.69128600
H	3.88387400	3.14650400	-2.38951500
C	4.76176900	6.42437000	-2.32439600
H	3.87544500	7.91373100	-1.04028700
H	5.44491700	4.71578900	-3.44546400
H	5.45592000	7.11544700	-2.79164100
N	-0.51492000	4.45738700	-0.39458900
O	-0.43795600	4.62985800	0.81419900
O	-1.45274600	4.84498100	-1.08683700
H	-0.76117400	1.33660200	0.74157600
H	1.72487400	1.32319900	1.68062300
C	0.14688600	1.58528800	0.20699200
C	1.40257500	1.57529400	0.67736400

7. DFT Calculation of the Transition State

7.1 Computational details

Orientating density functional (DFT) calculations were performed with the Gaussian16 (Revision B.01).[24] The M06-2X density functional was used, which is recommended for weak non-covalent interactions.[26,27] The basis set (def2-TZVP(D))[28,29] was of triple-zeta quality, and the corresponding pseudopotential for iodine was employed.[30] The nature of the obtained transition state structure was confirmed by the expected number of imaginary frequencies (N_{imag}=1). Grimme's low-frequency entropy corrections[31] have been applied to the Gibbs free energy. The counteranions of catalyst **4** were omitted in this calculation. The graphic was produced using CYLview20.[25]

7.2 Transition state of the Diels-Alder reaction between *trans*-β-nitrostyrene (1a) and cyclopentadiene (2) involving catalyst 4 (without counteranions)

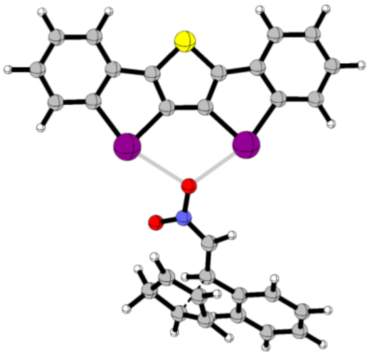

```
E (hartree) = -2315.486146
G (hartree) = -2315.123514
C      4.067623    -0.996251    -0.540161
H      3.835426    -1.675428    -1.353929
C      2.923918    -0.576569     0.177610
H      2.907329     0.221741     0.899094
N      1.716023    -1.110601    -0.088829
O      1.541648    -2.057736    -0.859403
O      0.677834    -0.586326     0.478394
C      5.167622    -0.055679    -0.824143
C      5.902978    -0.229967    -1.995210
C      5.487661     1.001734     0.031260
C      6.933882     0.642431    -2.315061
H      5.662540    -1.048852    -2.663593
C      6.517247     1.868227    -0.286078
H      4.947661     1.137977     0.961704
C      7.241764     1.690876    -1.462242
H      7.496015     0.500008    -3.227999
H      6.764476     2.681612     0.382952
H      8.048565     2.368885    -1.706283
C     -4.329993    -1.643170    -0.267246
C     -3.470023    -2.743314    -0.315533
C     -3.905931    -4.043929    -0.460536
C     -5.276663    -4.247337    -0.562157
C     -6.161871    -3.173681    -0.518074
C     -5.700259    -1.877280    -0.372115
C     -3.676006    -0.360633    -0.115024
H     -3.222082    -4.881153    -0.496543
H     -5.653808    -5.254459    -0.677247
H     -7.224891    -3.354756    -0.599072
H     -6.397176    -1.048740    -0.338985
C     -2.311475    -0.289339    -0.006441
C     -1.202228     3.696944     0.216062
C     -2.559431     3.376719     0.135020
C     -3.475470     4.427435     0.114121
C     -3.019907     5.733043     0.173316
C     -1.658822     6.013606     0.253640
C     -0.721765     4.987756     0.278074
C     -1.847526     1.036279     0.100324
C     -2.857867     1.961199     0.069448
H     -4.536800     4.220358     0.051373
H     -3.732062     6.546656     0.156369
H     -1.319558     7.039617     0.297636
H      0.334941     5.209387     0.340804
S     -4.390570     1.201281    -0.080065
I     -0.017368     1.965587     0.266645
I     -1.462914    -2.158666    -0.139759
C      3.472699    -2.704105     2.293418
H      2.603084    -2.926340     2.896546
C      4.499325    -1.882410     2.651435
H      4.596536    -1.352370     3.586960
C      5.425440    -1.829711     1.578841
H      6.347091    -1.263672     1.580687
C      4.939847    -2.546164     0.490957
H      5.571754    -2.875509    -0.322368
C      3.750596    -3.329216     0.977678
H      4.075928    -4.365068     1.150513
H      2.898891    -3.385448     0.300209
```

8. References

(1) Org. Synth. **1952**, *32*, 41.
(2) Fulmer, G. R.; Miller, A. J. M.; Sherden, N. H.; Gottlieb, H. E.; Nudelman, A.; Stoltz, B. M.; Bercaw, J. E.; Goldberg, K. I. Organometallics **2010**, *29*, 2176.
(3) He, H.; Chen, L.-Y.; Wong, W.-Y.; Chan, W.-H.; Lee, A. W. M. Eur. J. Org. Chem. **2010**, *2010*, 4181.
(4) He, G.; List, B.; Christmann, M. Angew. Chem. Int. Ed. **2021**, *60*, 13591.
(5) Do, H.-Q.; Khan, R. M. K.; Daugulis, O. J. Am. Chem. Soc. **2008**, *130*, 15185.
(6) Schnitte, M.; Lipinski, S.; Schiebel, E.; Mecking, S. Organometallics **2020**, *39*, 13.
(7) Liu, L.; Kim, H.; Xie, Y.; Farès, C.; Kaib, P. S. J.; Goddard, R.; List, B. J. Am. Chem. Soc. **2017**, *139*, 13656.
(8) Reinhard, D. L.; Kutzinski, D.; Hatta, M.; Engelage, E.; Huber, S. M. Synlett **2024**, *35*, 209.
(9) Heinen, F.; Engelage, E.; Dreger, A.; Weiss, R.; Huber, S. M. Angew. Chem. Int. Ed. **2018**, *57*, 3830.
(10) Reinhard, D. L.; Schmidt, A.; Sons, M.; Wolf, J.; Engelage, E.; Huber, S. M. Beilstein J. Org. Chem. **2024**, *20*, 2401.
(11) Kaib, P. S. J.; Schreyer, L.; Lee, S.; Properzi, R.; List, B. Angew. Chem. Int. Ed. **2016**, *55*, 13200.
(12) Lee, S.; Kaib, P. S. J.; List, B. J. Am. Chem. Soc. **2017**, *139*, 2156.
(13) Mahlau, M.; García-García, P.; List, B. Chem. Eur. J. **2012**, *18*, 16283.
(14) Guin, J.; Rabalakos, C.; List, B. Angew. Chem. Int. Ed. **2012**, *51*, 8859.
(15) Wang, Q.; Leutzsch, M.; Van Gemmeren, M.; List, B. J. Am. Chem. Soc. **2013**, *135*, 15334.
(16) Tap, A.; Blond, A.; Wakchaure, V. N.; List, B. Angew. Chem. Int. Ed. **2016**, *55*, 8962.
(17) Wakchaure, V. N.; Obradors, C.; List, B. Synlett **2020**, *31*, 1707.
(18) Murphy, J. M.; Tzschucke, C. C.; Hartwig, J. F. Org. Lett. **2007**, *9*, 757.
(19) Reinhard, D. L.; Heinen, F.; Stoesser, J.; Engelage, E.; Huber, S. M. Helv. Chim. Acta **2021**, *104*, e2000221.
(20) Novakov, I. A.; Orlinson, B. S.; Brunilin, R. V.; Navrotskii, M. B.; Eremiichuk, A. S.; Dumler, S. A.; Gordeeva, E. A. Pharm. Chem. J. **2011**, *45*, 419.
(21) Praxis Biosciences, WO2017048720A1 (2017) .
(22) Halgren, T. A. Journal of Computational Chemistry **1996**, *17*, 490.
(23) Spartan 14, Wavefunction Inc., Irvine, CA, USA .
(24) **Gaussian 16 Rev. B.01**; Frisch, M. J.; Trucks, G. W.; Schlegel, H. B.; Scuseria, G. E.; Robb, M. A.; Cheeseman, J. R.; Scalmani, G.; Barone, V.; Petersson, G. A.; Nakatsuji, H.; Li, X.; Caricato, M.; Marenich, A. V.; Bloino, J.; Janesko, B. G.; Gomperts, R.; Mennucci, B.; Hratchian, H. P.; Ortiz, J. V.; Izmaylov, A. F.; Sonnenberg, J. L.; Williams-Young, D.; Ding, F.; Lipparini, F.; Egidi, F.; Goings, J.; Peng, B.; Petrone, A.; Henderson, T.; Ranasinghe, D.; Zakrzewski, V. G.; Gao, J.; Rega, N.; Zheng, G.; Liang, W.; Hada, M.; Ehara, M.; Toyota, K.; Fukuda, R.; Hasegawa, J.; Ishida, M.; Nakajima, T.; Honda, Y.; Kitao, O.; Nakai, H.; Vreven, T.; Throssell, K.; Montgomery Jr., J. A.; Peralta, J. E.; Ogliaro, F.; Bearpark, M. J.; Heyd, J. J.; Brothers, E. N.; Kudin, K. N.; Staroverov, V. N.; Keith, T. A.; Kobayashi, R.; Normand, J.; Raghavachari, K.; Rendell, A. P.; Burant, J. C.; Iyengar, S. S.; Tomasi, J.; Cossi, M.; Millam, J. M.; Klene, M.; Adamo, C.; Cammi, R.; Ochterski, J. W.; Martin, R. L.; Morokuma, K.; Farkas, O.; Foresman, J. B.; Fox, D. J.; Wallingford, CT **2016**.
(25) **CYLview20**; Legault, C. Y. **2020**.
(26) Zhao, Y.; Truhlar, D. G. Theor Chem Account **2008**, *119*, 525.
(27) Grimme, S.; Antony, J.; Ehrlich, S.; Krieg, H. J. Chem. Phys. **2010**, *132*, 154104.
(28) Weigend, F.; Ahlrichs, R. Phys. Chem. Chem. Phys. **2005**, *7*, 3297.
(29) Rappoport, D.; Furche, F. The Journal of Chemical Physics **2010**, *133*, 134105.
(30) Peterson, K. A.; Figgen, D.; Goll, E.; Stoll, H.; Dolg, M. The Journal of Chemical Physics **2003**, *119*, 11113.
(31) Grimme, S. Chem. Eur. J. **2012**, *18*, 9955.

9. NMR spectra

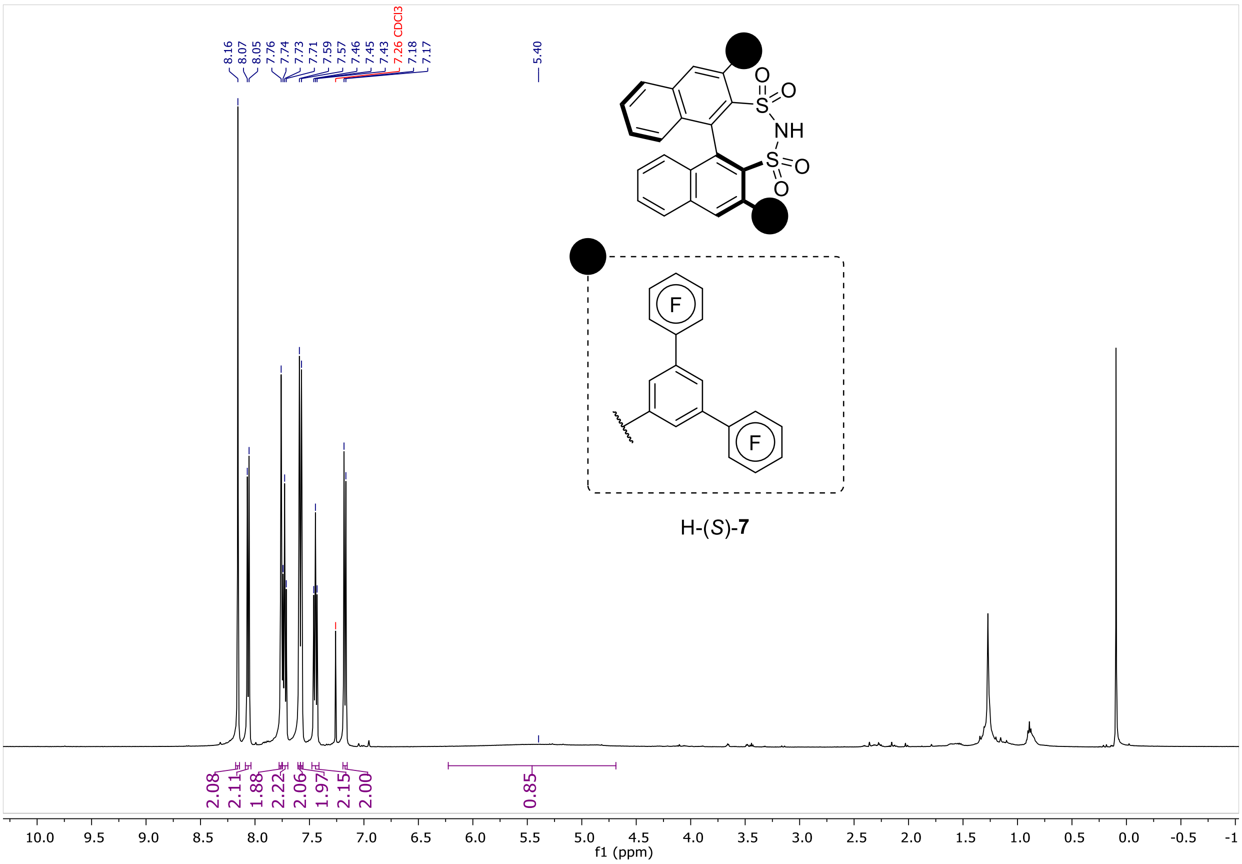

Figure S7: ^{1}H NMR of H-(*S*)-**7** in $CDCl_3$. The signals at ~0.85 and ~1.25 ppm correspond to grease.[2]

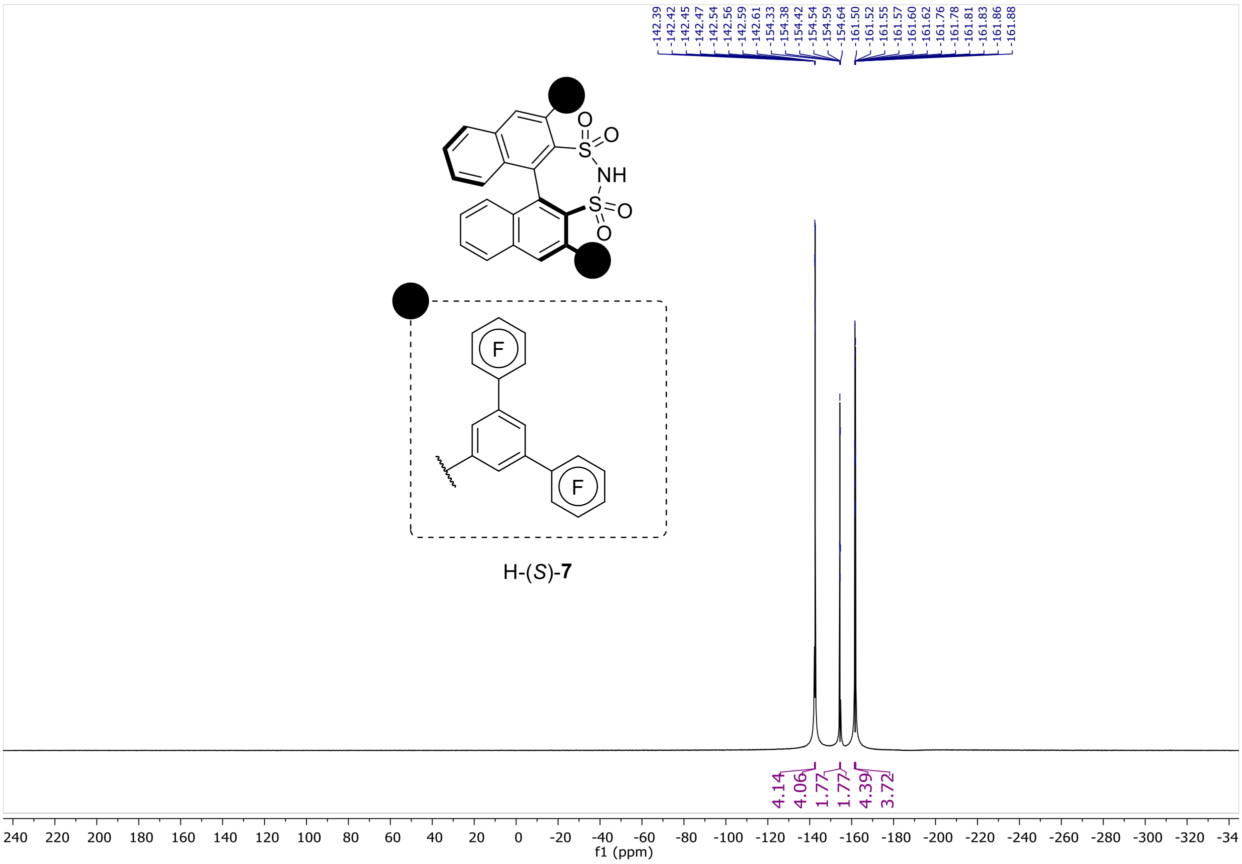

Figure S8: ^{19}F NMR of H-(*S*)-**7** in $CDCl_3$.

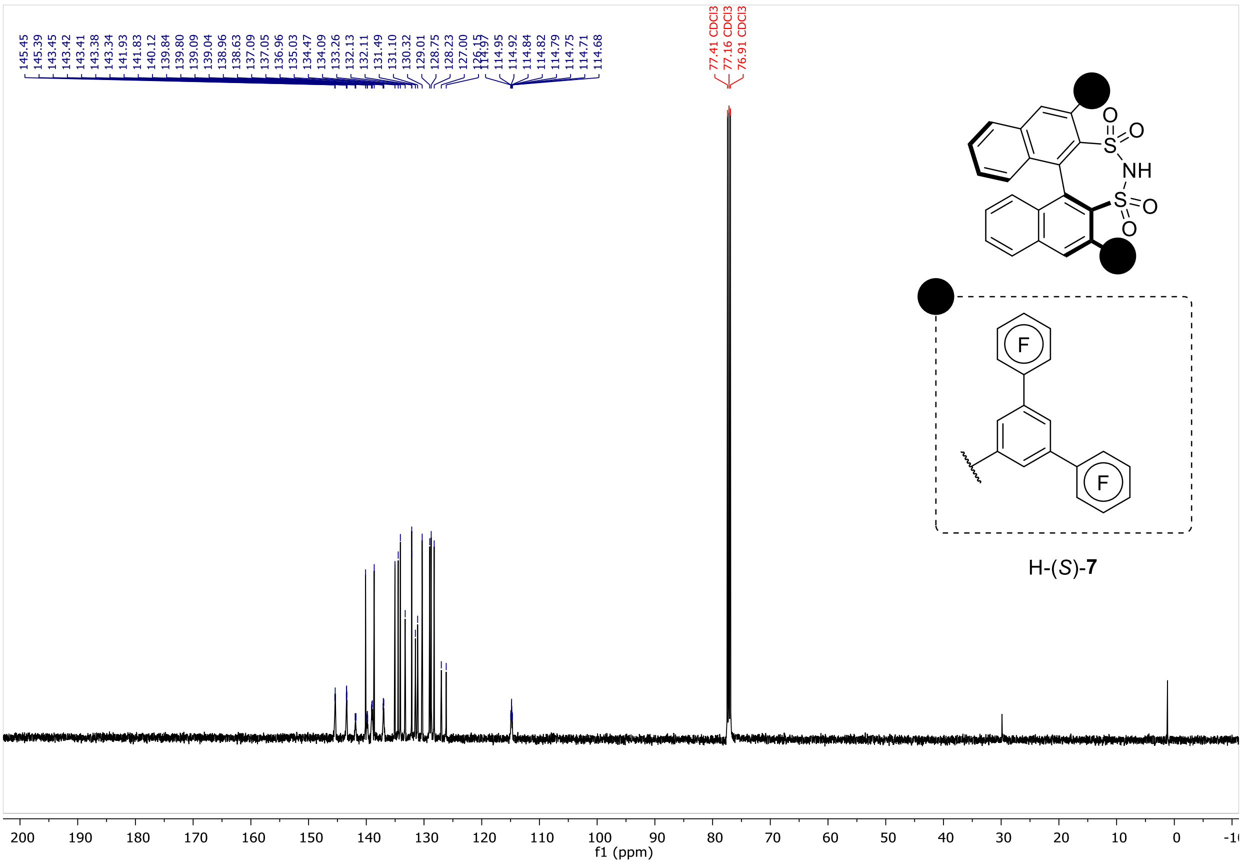

Figure S9: $^{13}C\{^{1}H\}$ NMR of H-(*S*)-**7** in $CDCl_3$. The signal at ~30 ppm corresponds to grease.[2]

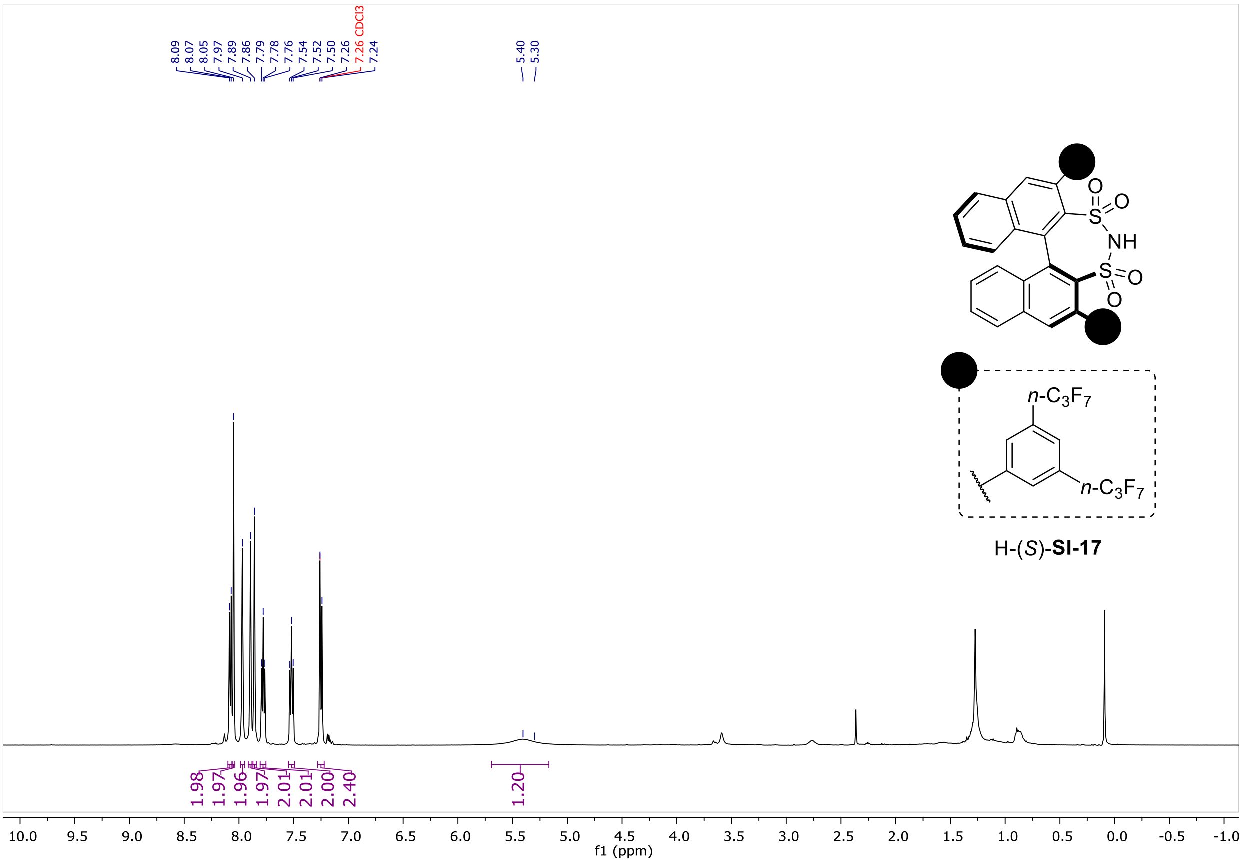

Figure S10: ^{1}H NMR of H-(*S*)-**SI-17** in $CDCl_3$. The signals at ~0.85 and ~1.25 ppm correspond to grease.[2]

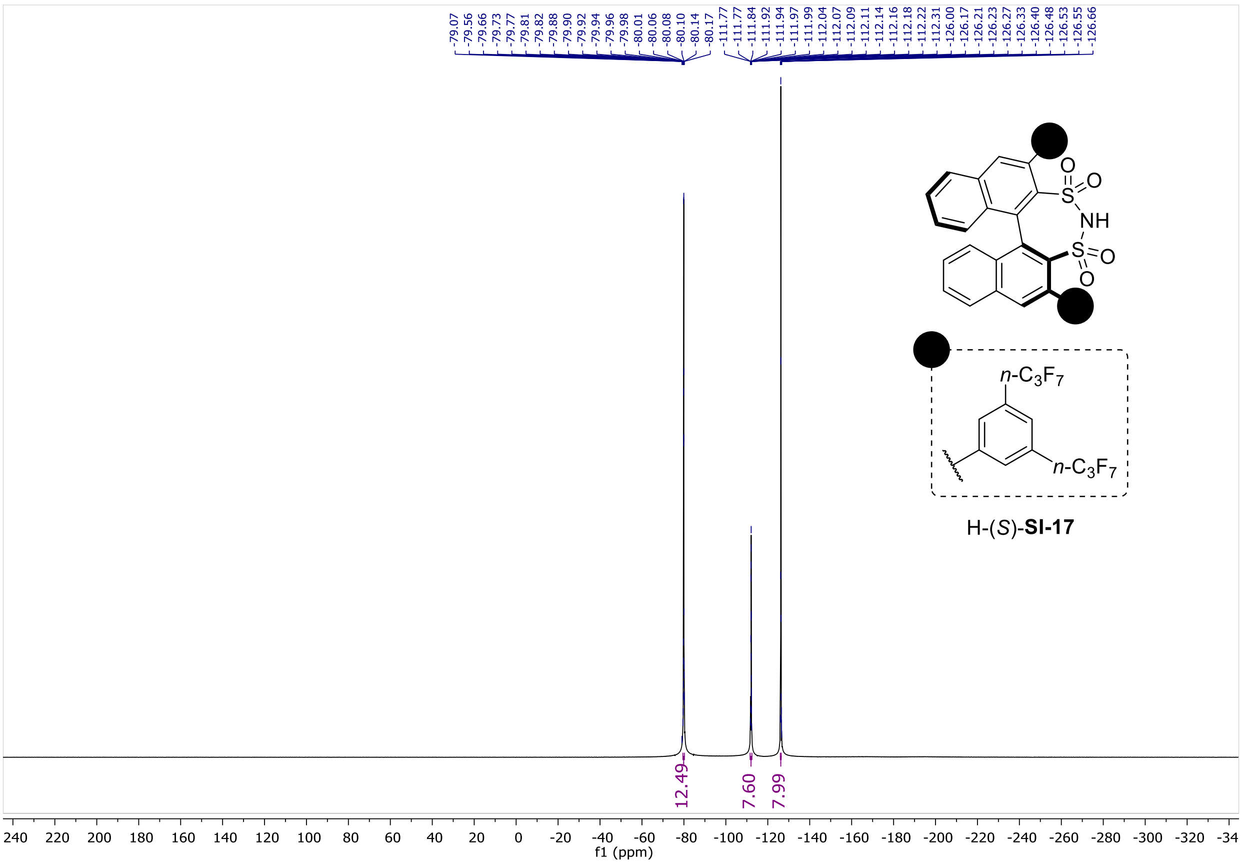

Figure S11: ^{19}F NMR of H-(*S*)-**SI-17** in $CDCl_3$.

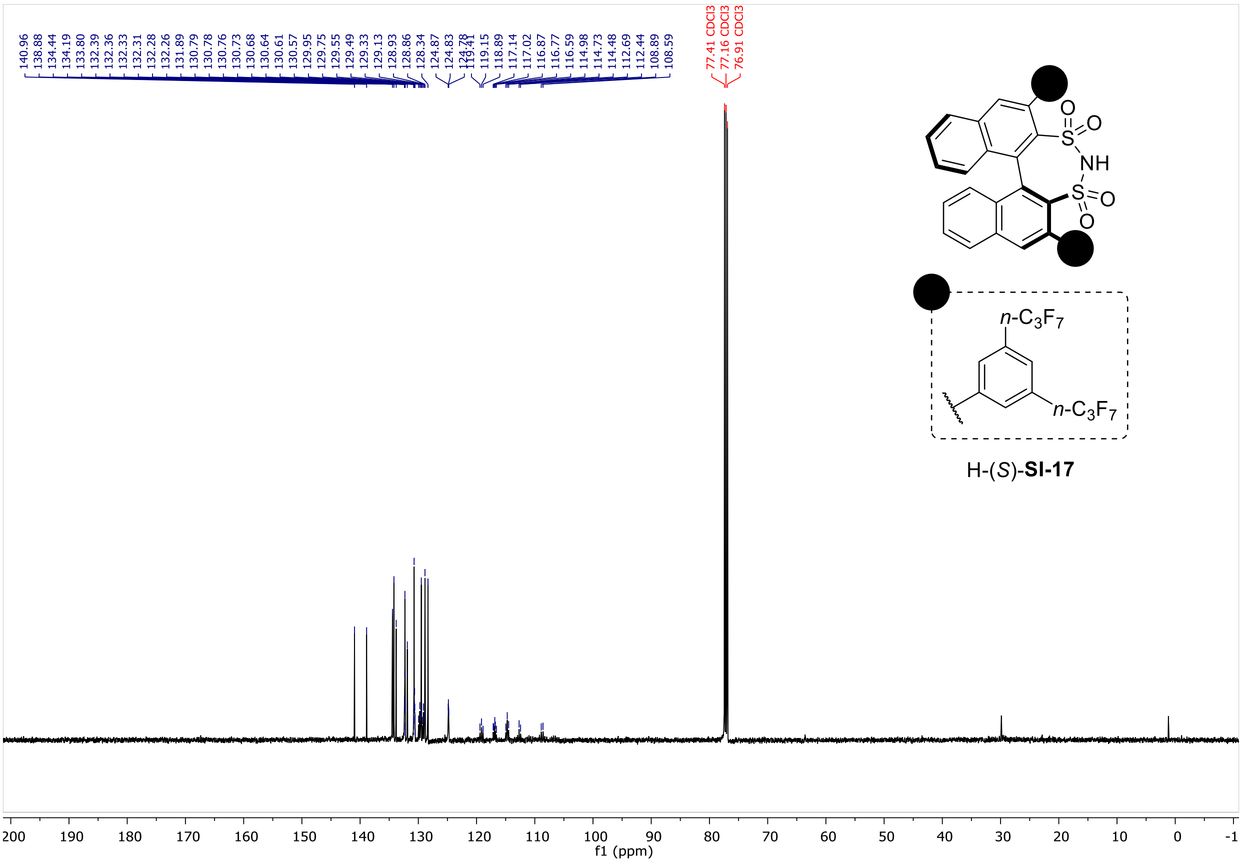

Figure S12: $^{13}C\{^1H\}$ NMR of H-(*S*)-**7** in $CDCl_3$. The signal at ~30 ppm corresponds to grease.[2]

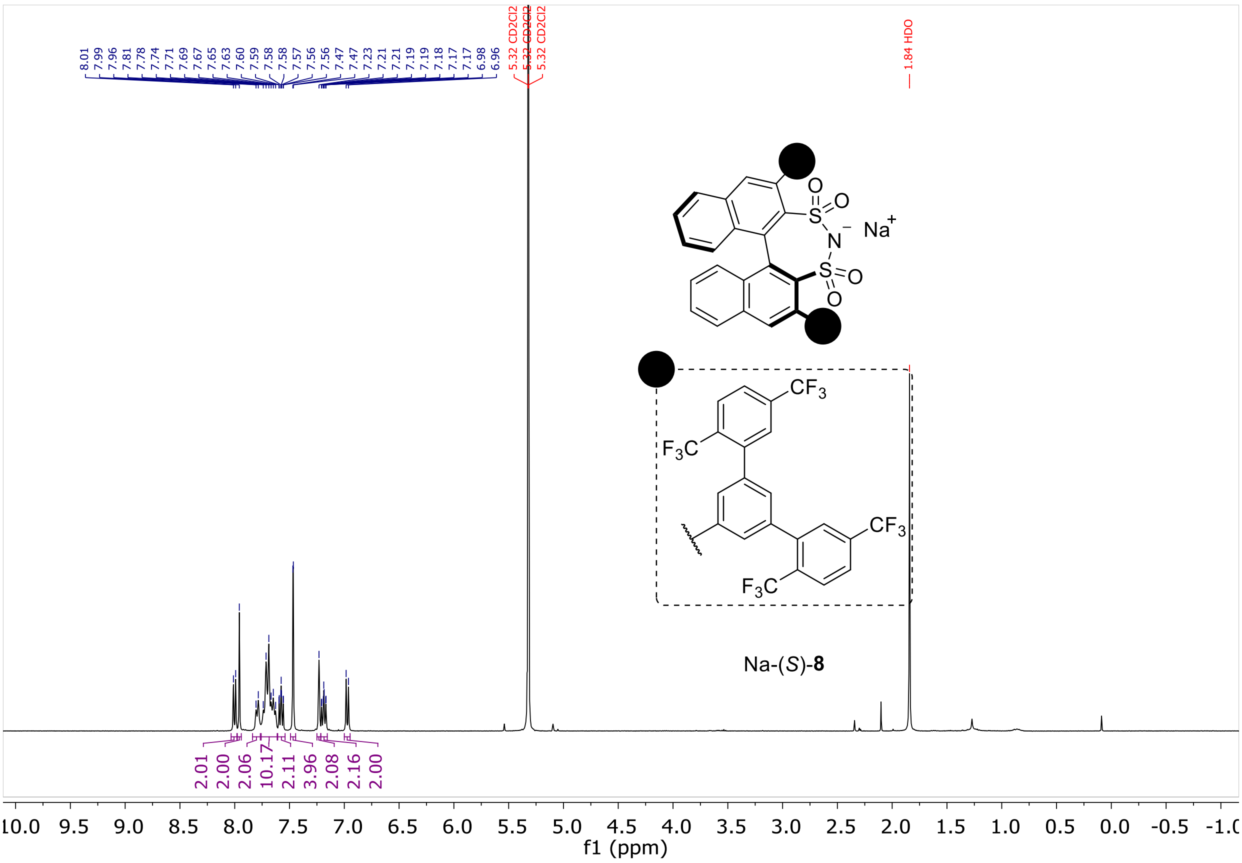

Figure S13: ^{1}H NMR of Na-(*S*)-**8** in CD_2Cl_2. The signals at ~0.85 and ~1.25 ppm correspond to grease.[2]

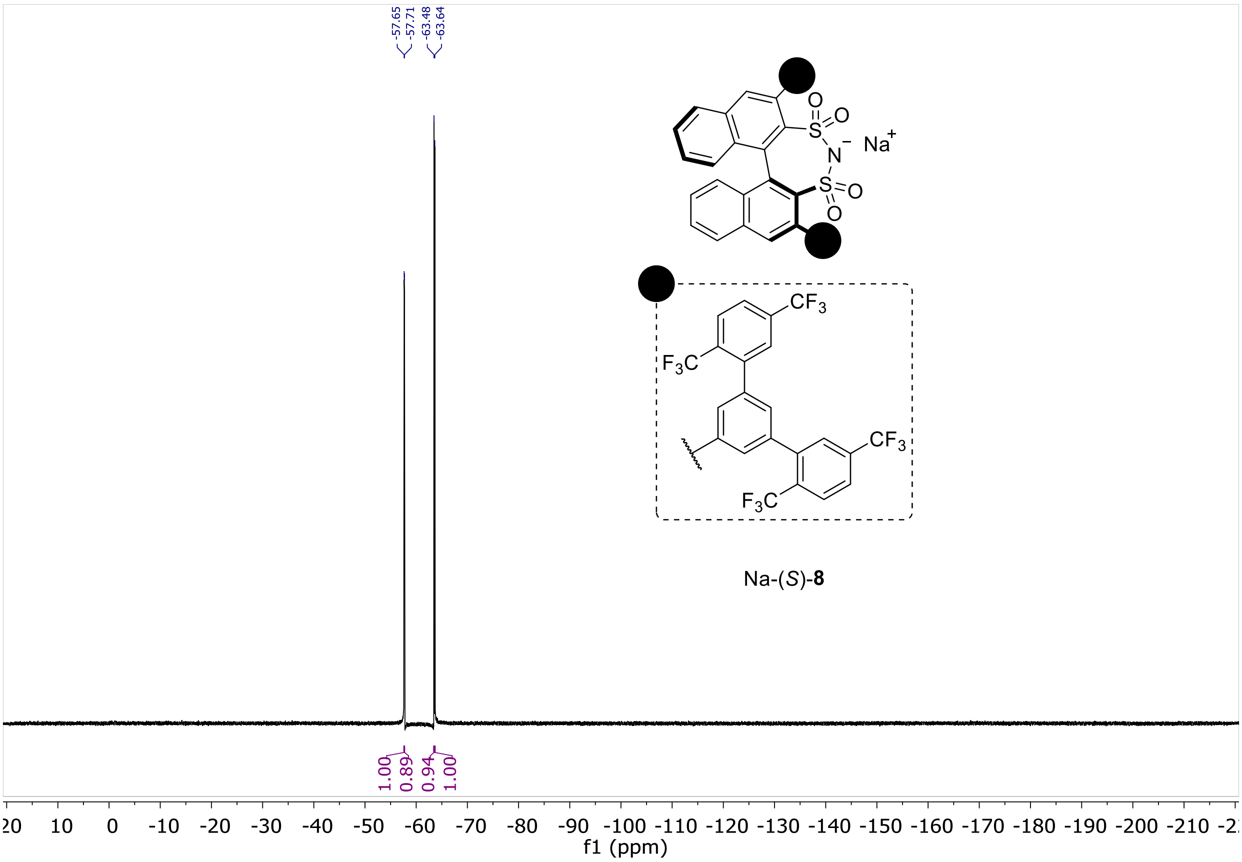

Figure S14: ^{19}F NMR of Na-(*S*)-**8** in CD_2Cl_2.

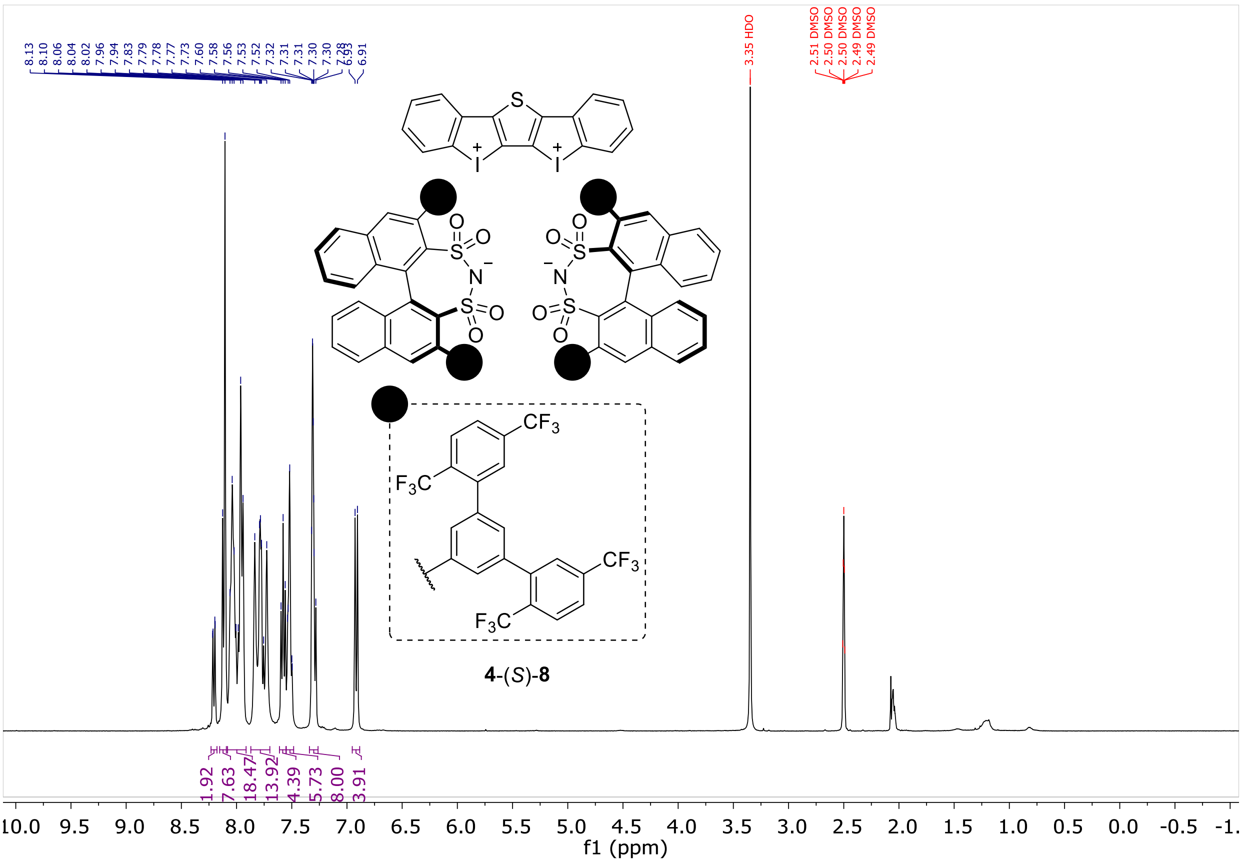

Figure S15: ^{1}H NMR of **4**-(*S*)-**8** in DMSO-*d6*. The signals at ~0.85 and ~1.25 ppm correspond to grease.[2]

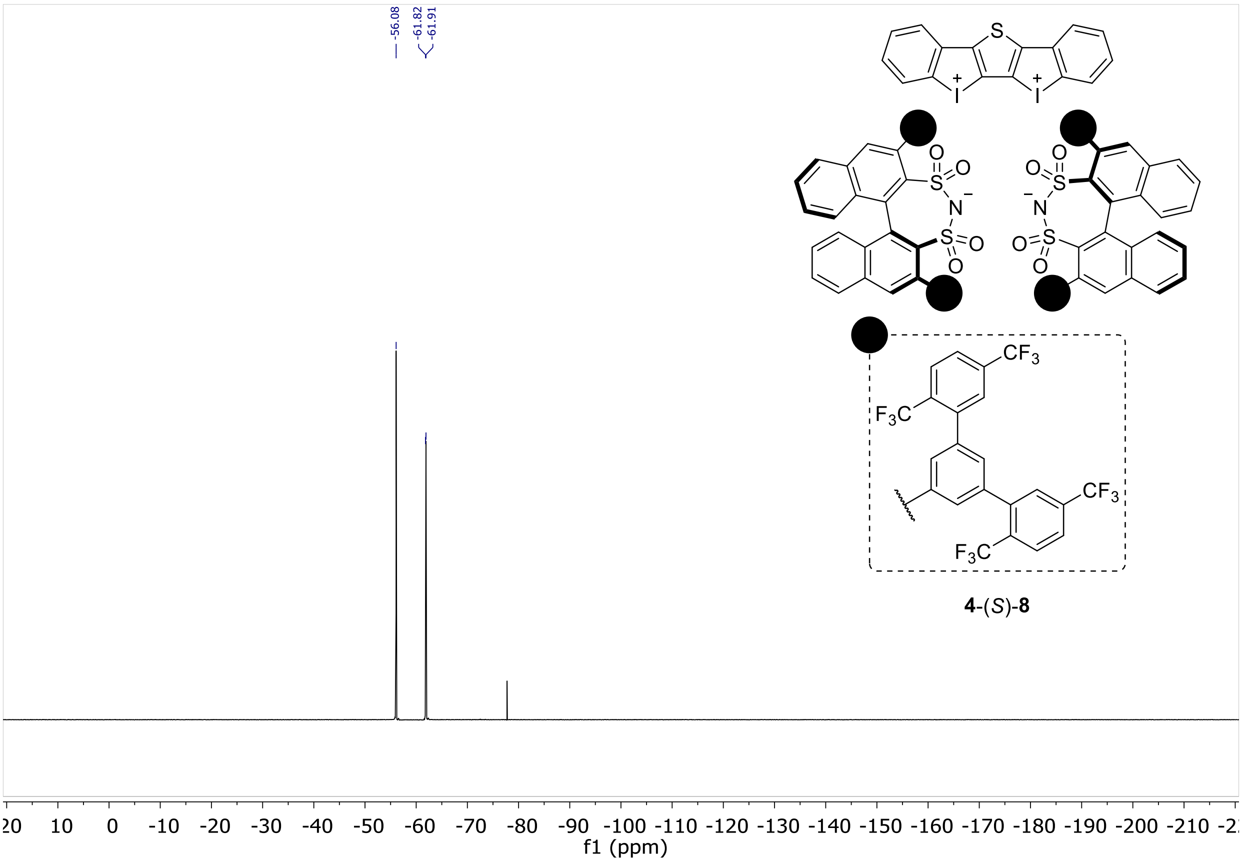

Figure S16: ^{19}F NMR of **4**-(*S*)-**8** in DMSO-*d6*.

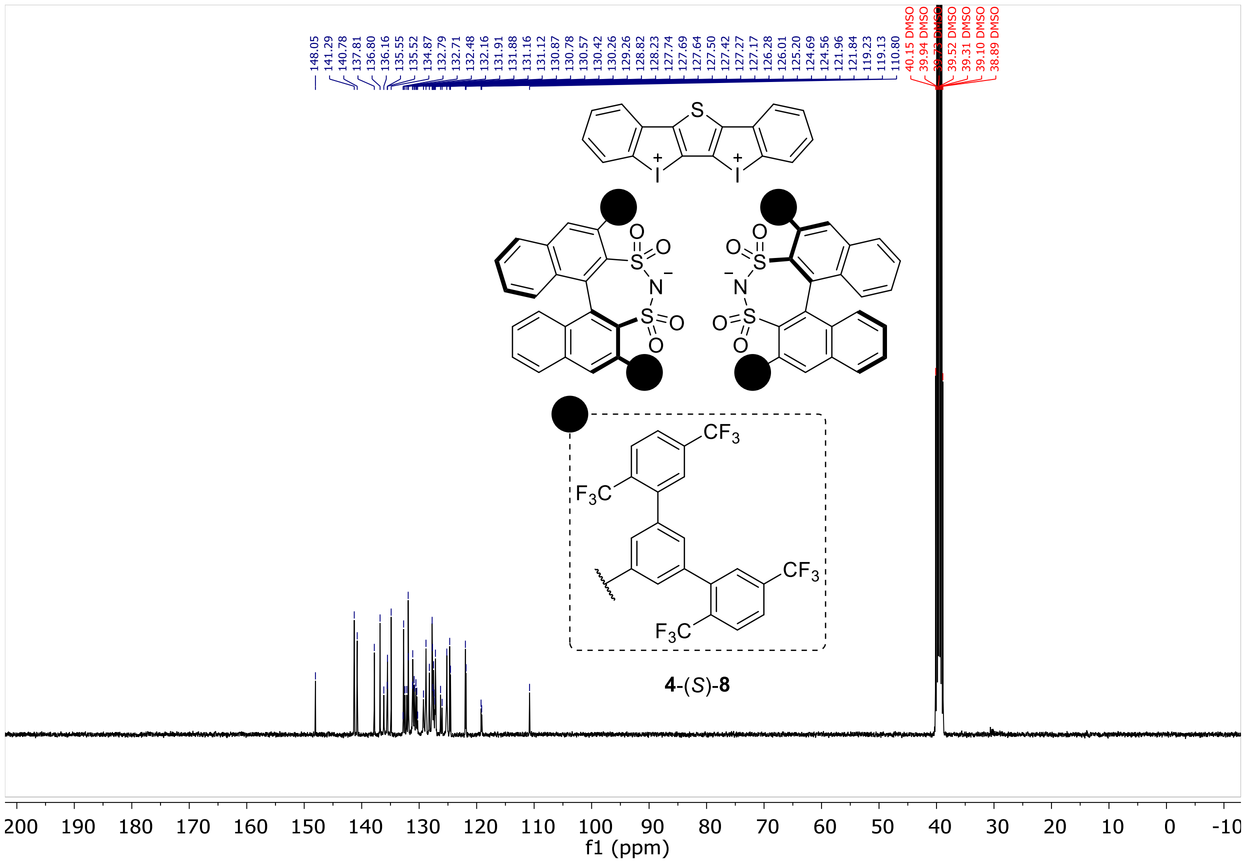

Figure S17: $^{13}C\{^{1}H\}$ NMR of **4**-(*S*)-**8** in DMSO-*d6*.

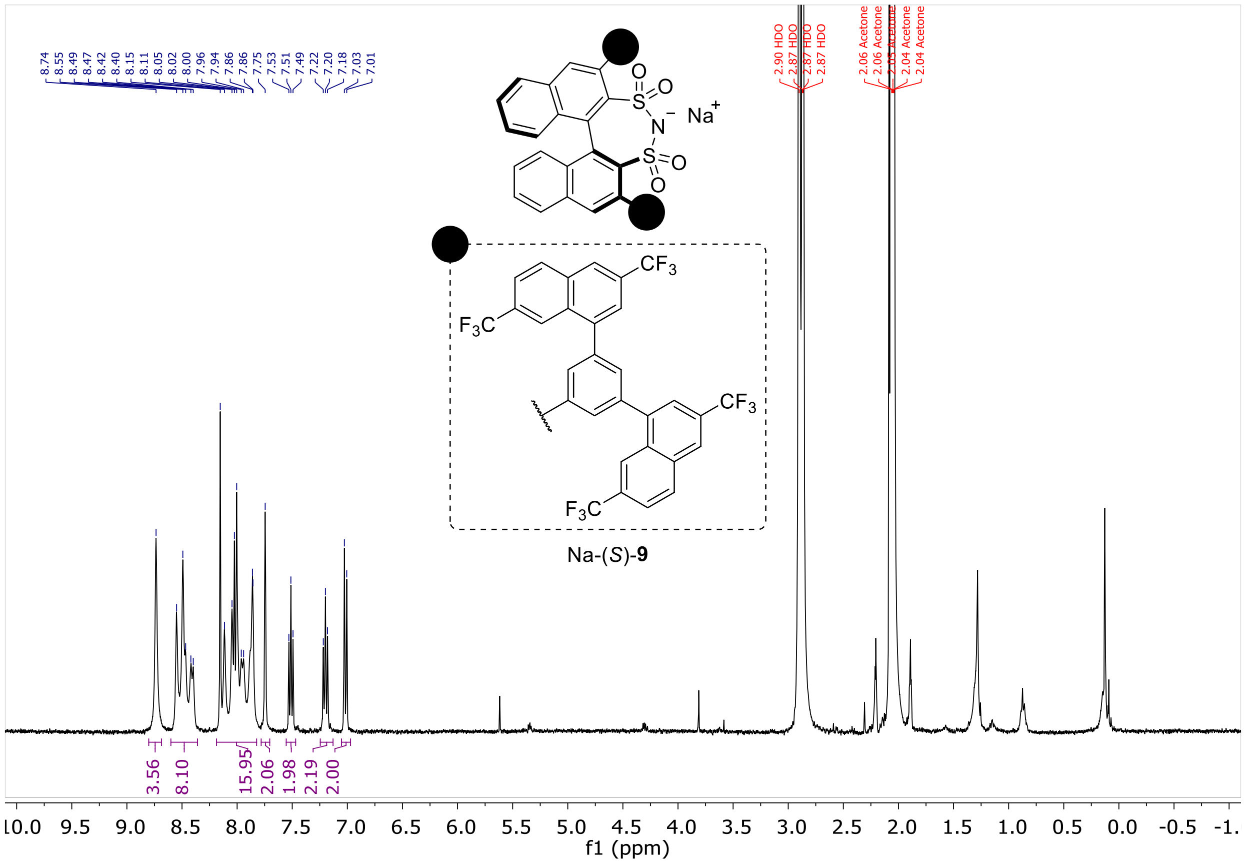

Figure S18: ^{1}H NMR of Na-(*S*)-**9** in acetone-*d6*. The signals at ~0.85 and ~1.25 ppm correspond to grease.[2]

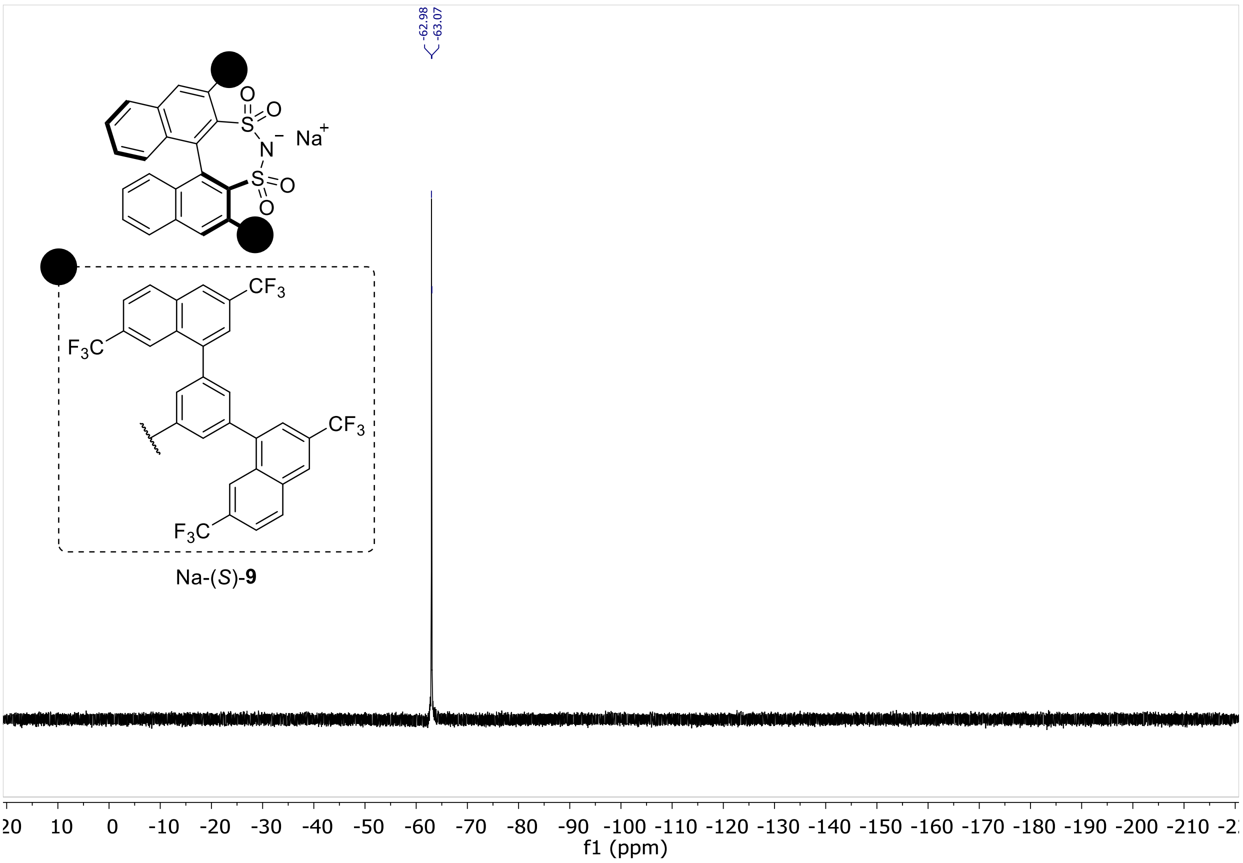

Figure S19: ^{19}F NMR of Na-(*S*)-**9** in acetone-*d6*.

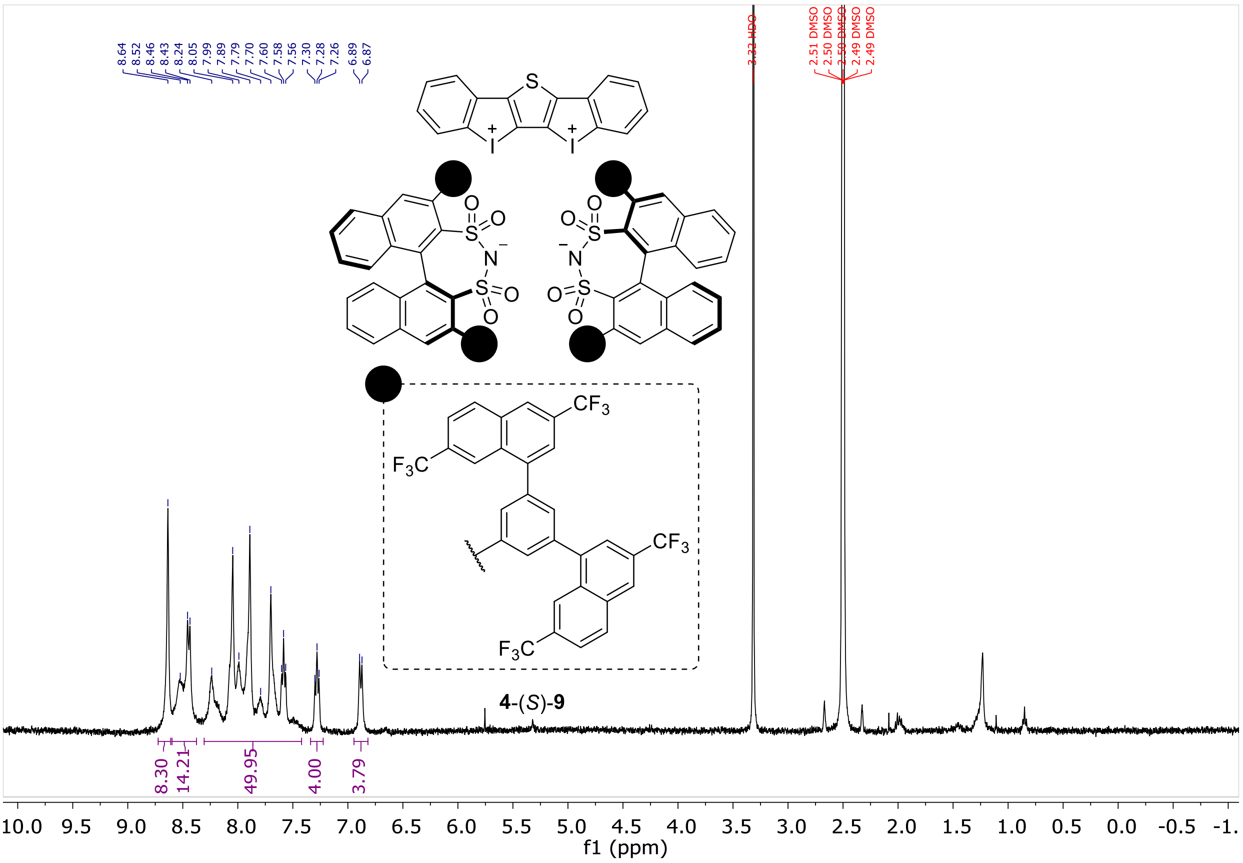

Figure S20: ^{1}H NMR of **4**-(*S*)-**9** in DMSO-*d6*. The signals at ~0.85 and ~1.25 ppm correspond to grease.[2]

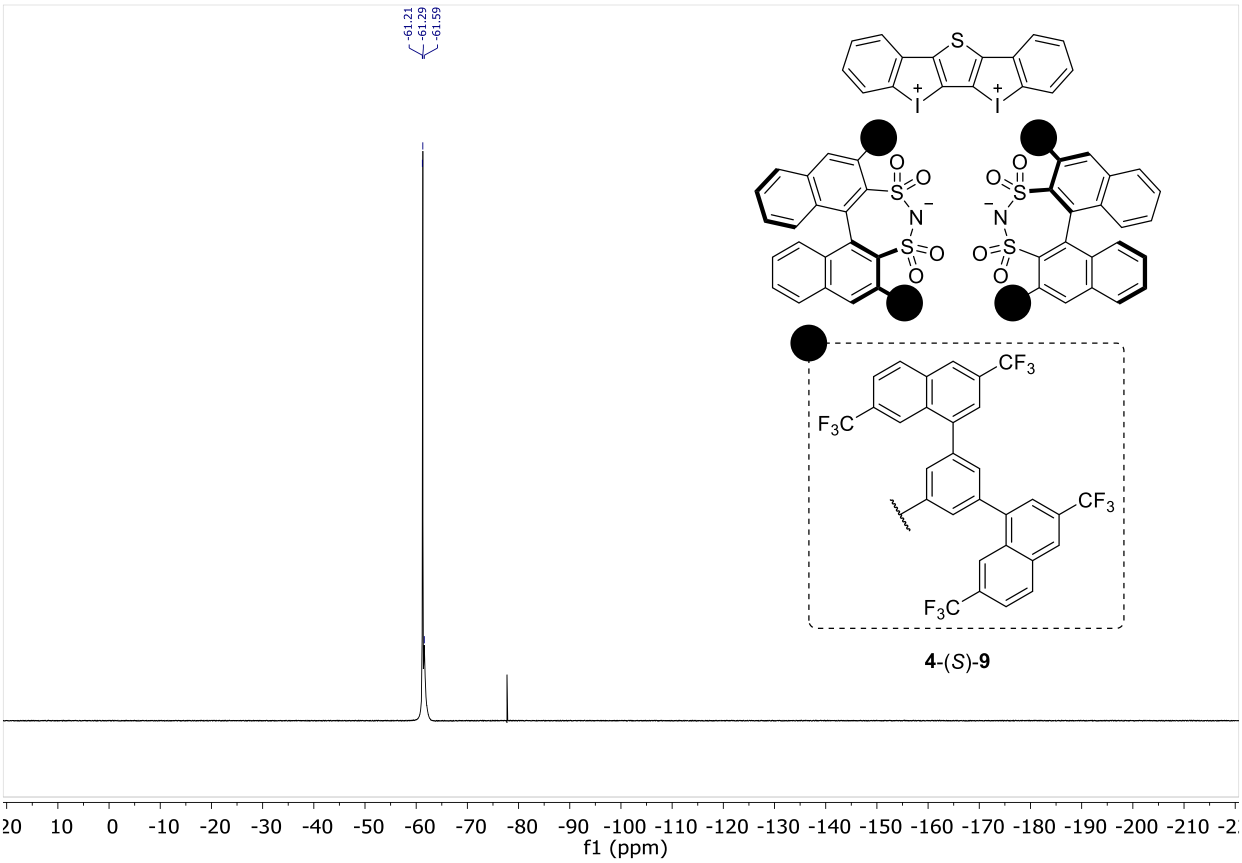

Figure S21: ^{19}F NMR of **4**-(*S*)-**9** in DMSO-*d6*.

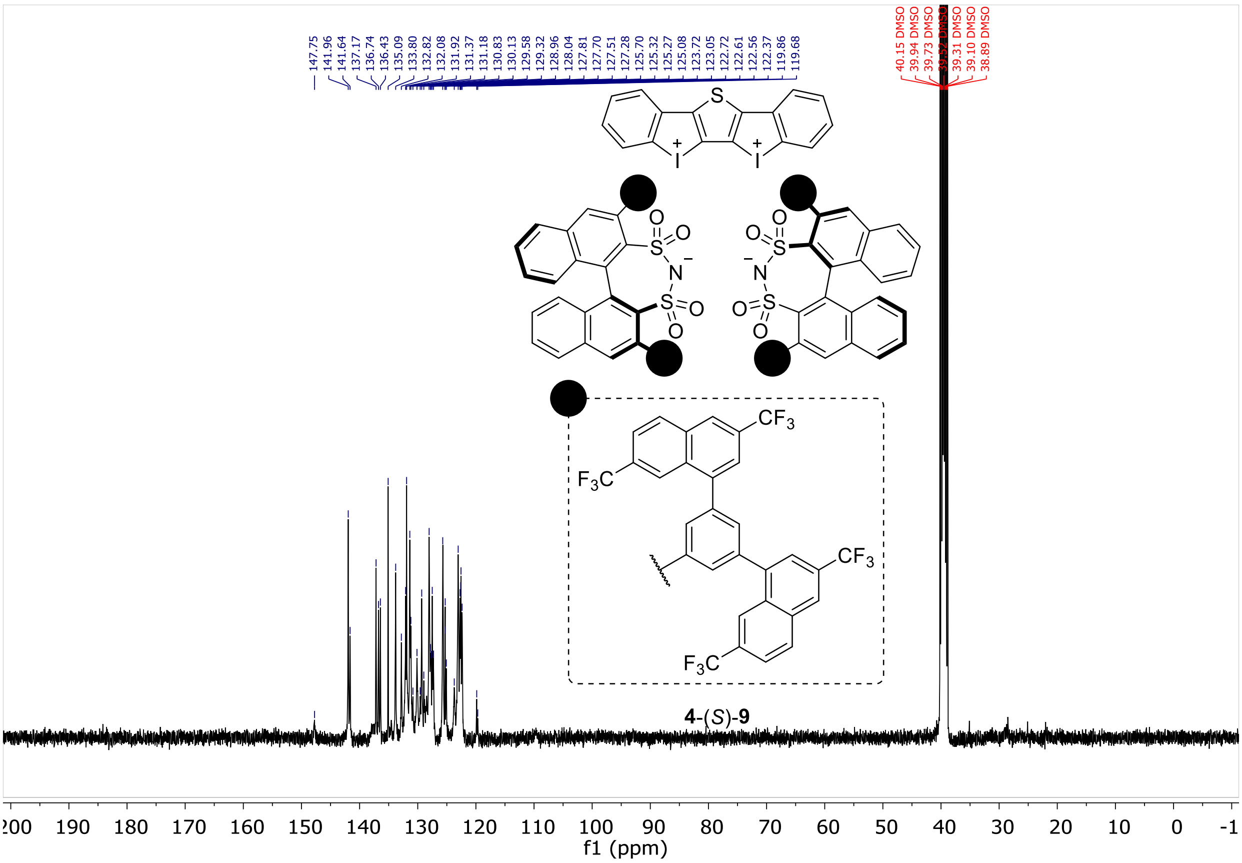

Figure S22: ^{13}C{^{1}H} NMR of **4**-(*S*)-**9** in DMSO-*d6*.

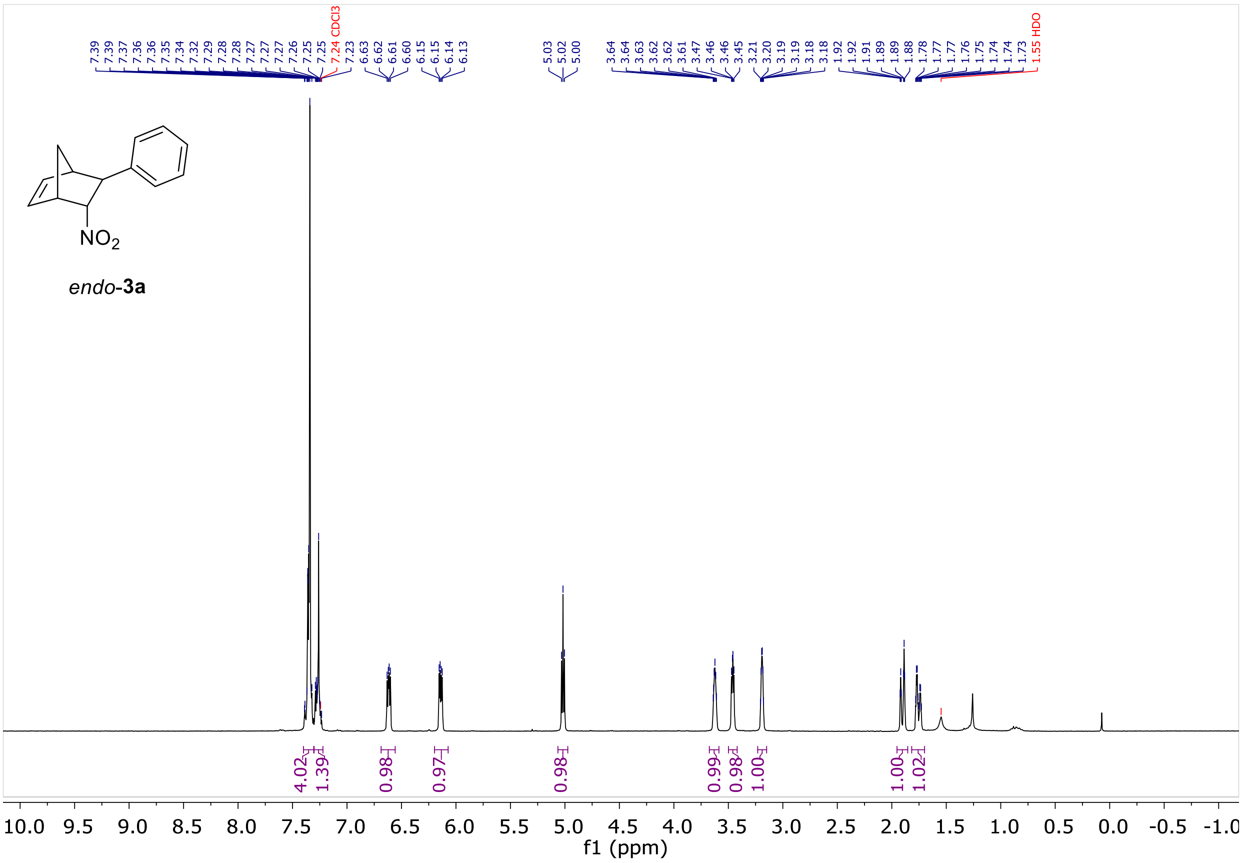

Figure S23: ^{1}H NMR of *endo*-**3a** in $CDCl_3$. The signals at ~0.85 and ~1.25 ppm correspond to grease.[2]

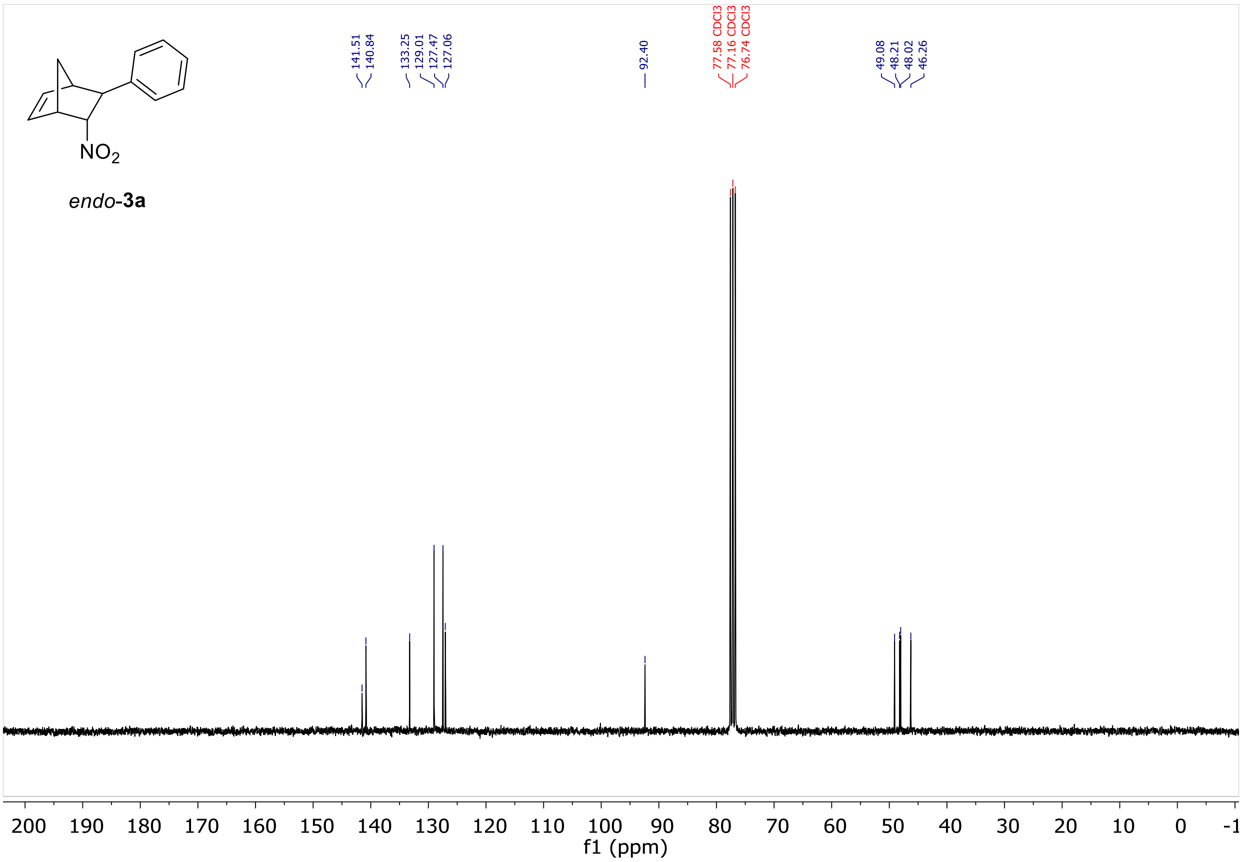

Figure S24: $^{13}C\{^{1}H\}$ NMR of *endo*-**3a** in $CDCl_3$.

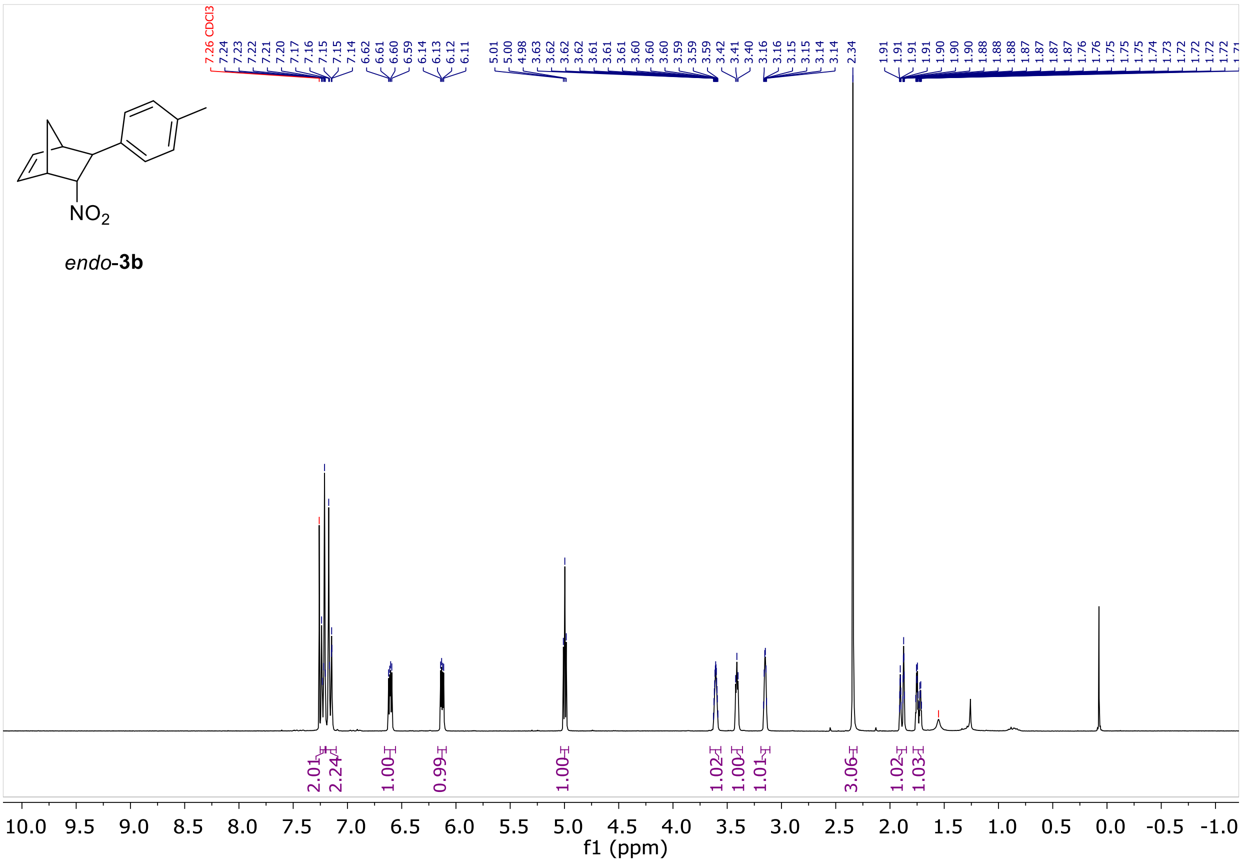

Figure S25: ^{1}H NMR of *endo*-**3b** in $CDCl_3$. The signals at ~0.85 and ~1.25 ppm correspond to grease.[2]

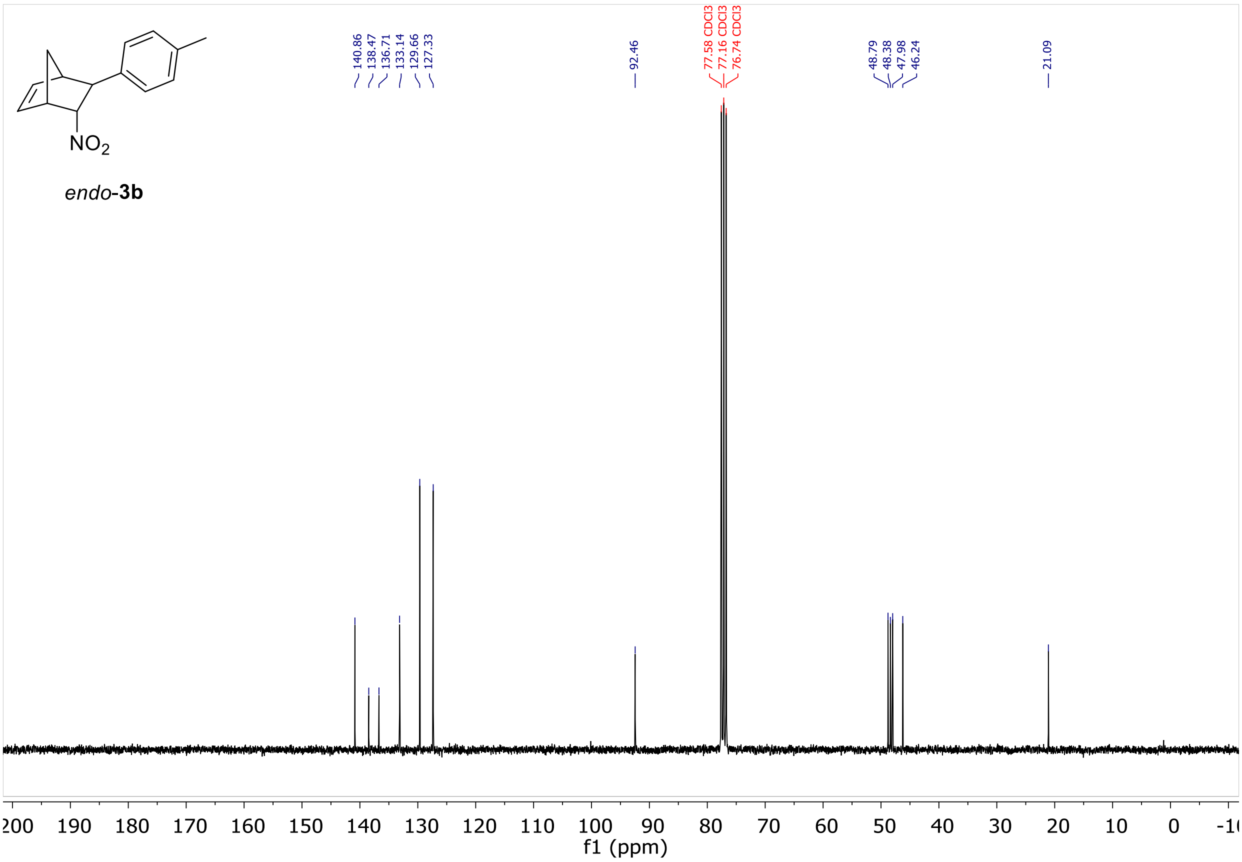

Figure S26: $^{13}C\{^1H\}$ NMR of *endo*-**3b** in $CDCl_3$.

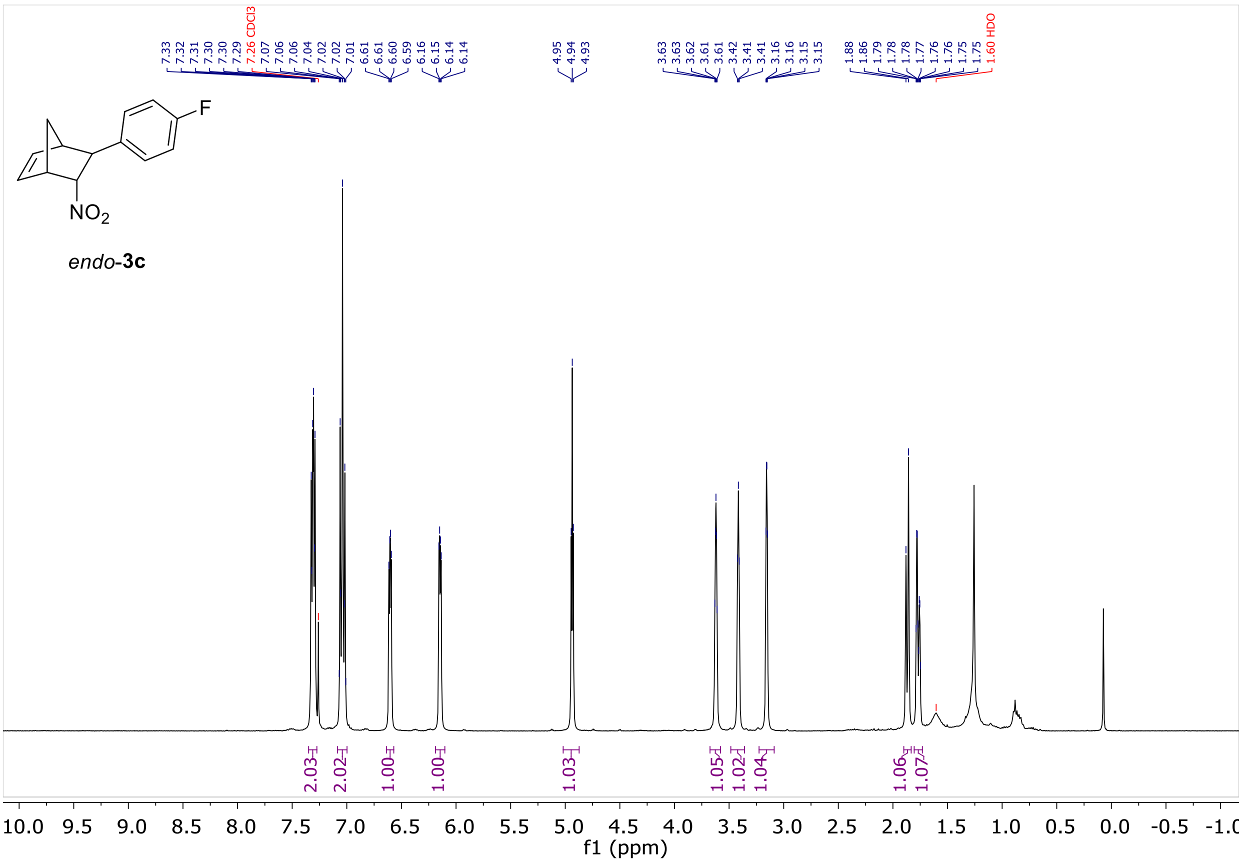

Figure S27: ^{1}H NMR of *endo*-**3c** in $CDCl_3$. The signals at ~0.85 and ~1.25 ppm correspond to grease.[2]

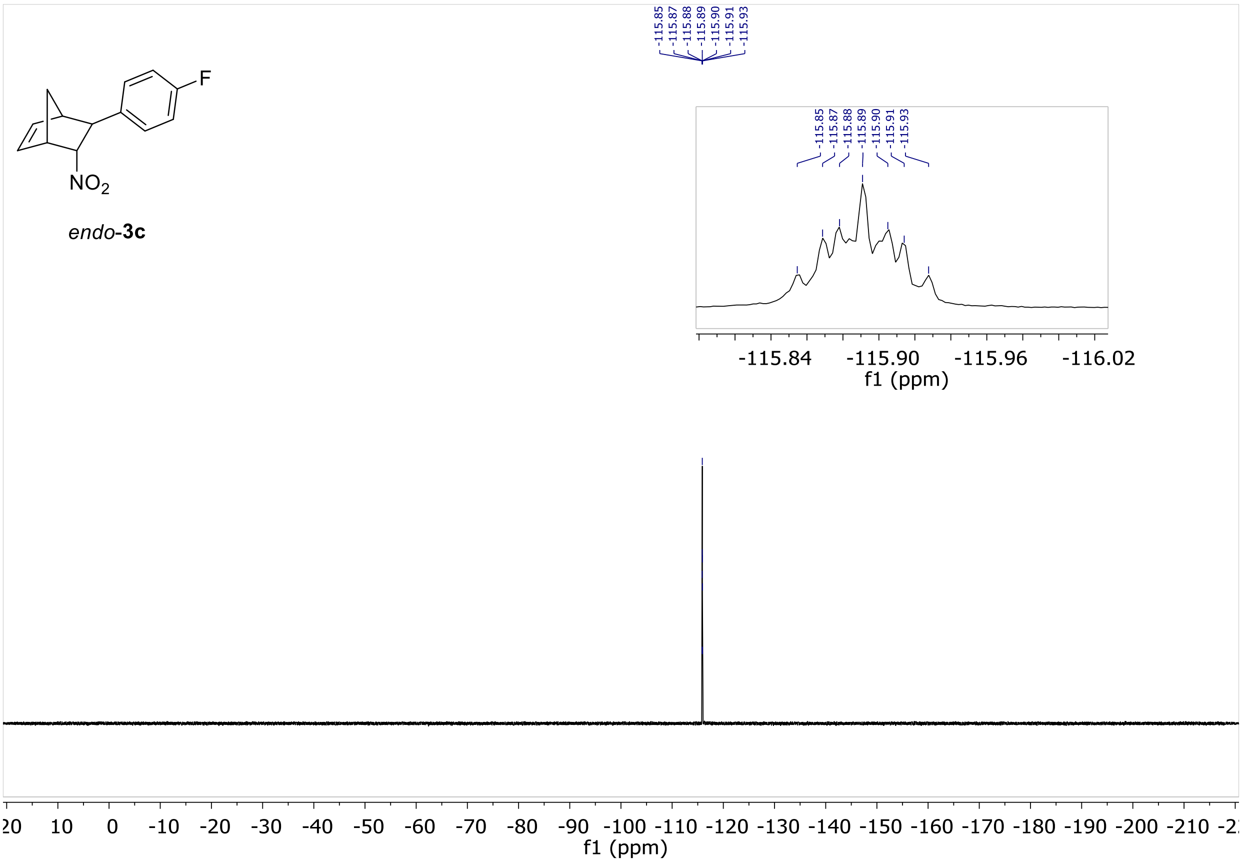

Figure S28: ^{19}F NMR of *endo*-**3c** in $CDCl_3$.

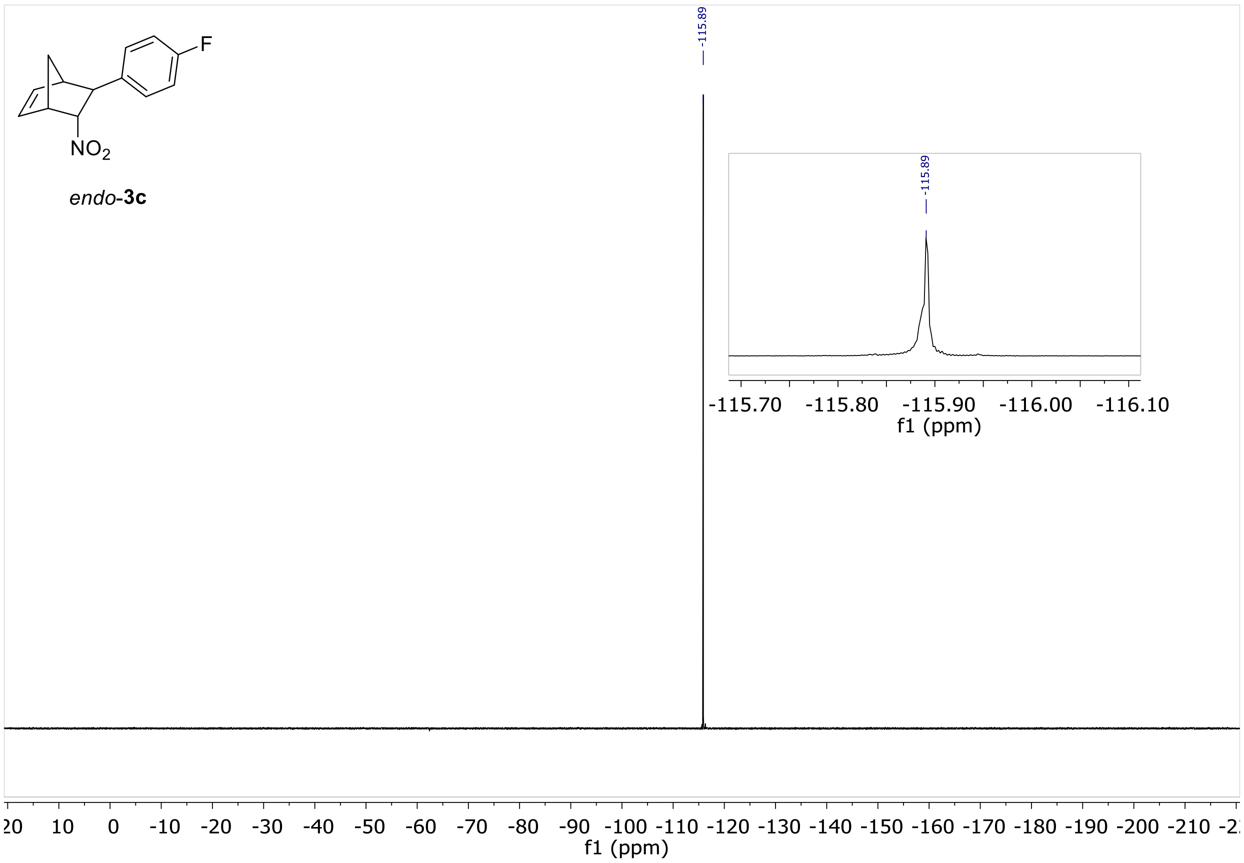

Figure S29: $^{19}F\{^{1}H\}$ NMR of *endo*-**3c** in $CDCl_3$.

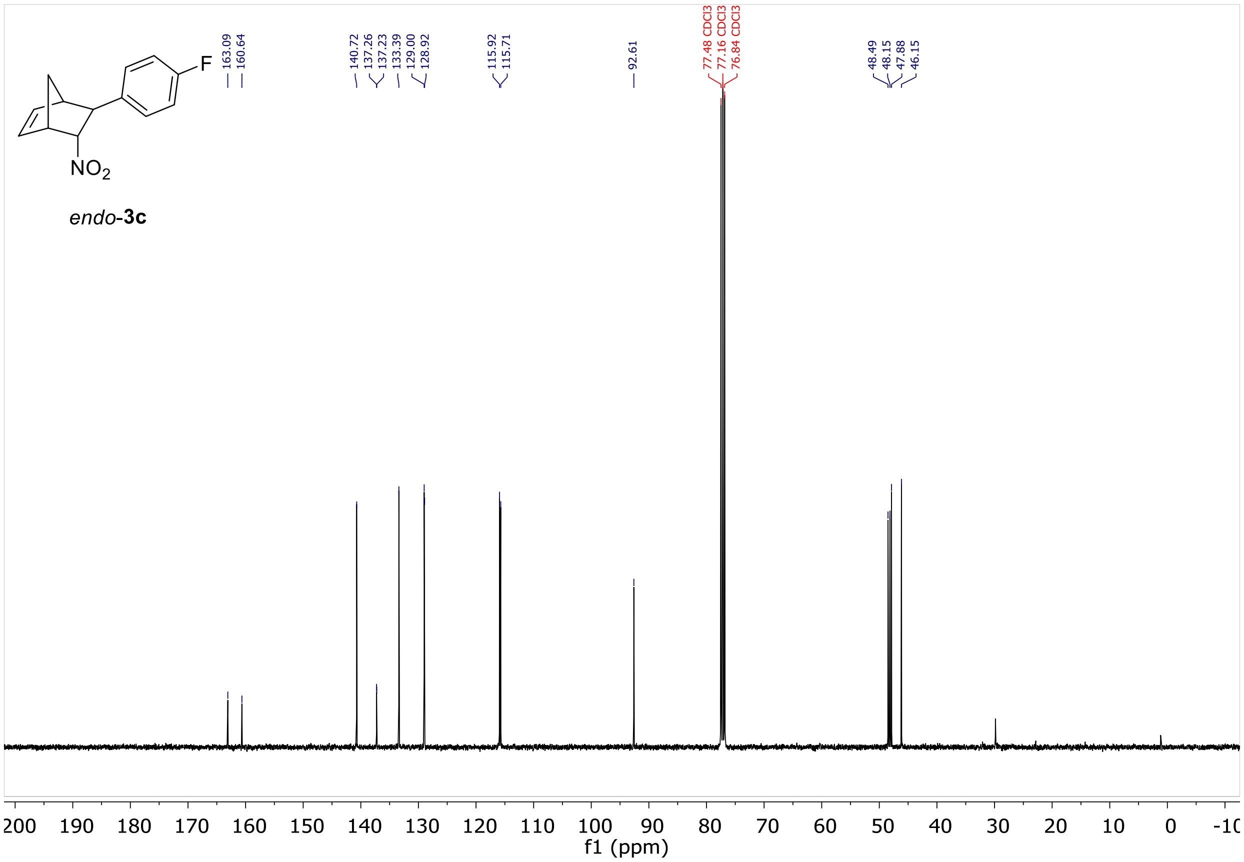

Figure S30: $^{13}C\{^{1}H\}$ NMR of *endo*-**3c** in $CDCl_3$. The signal at ~30 ppm corresponds to grease.[2]

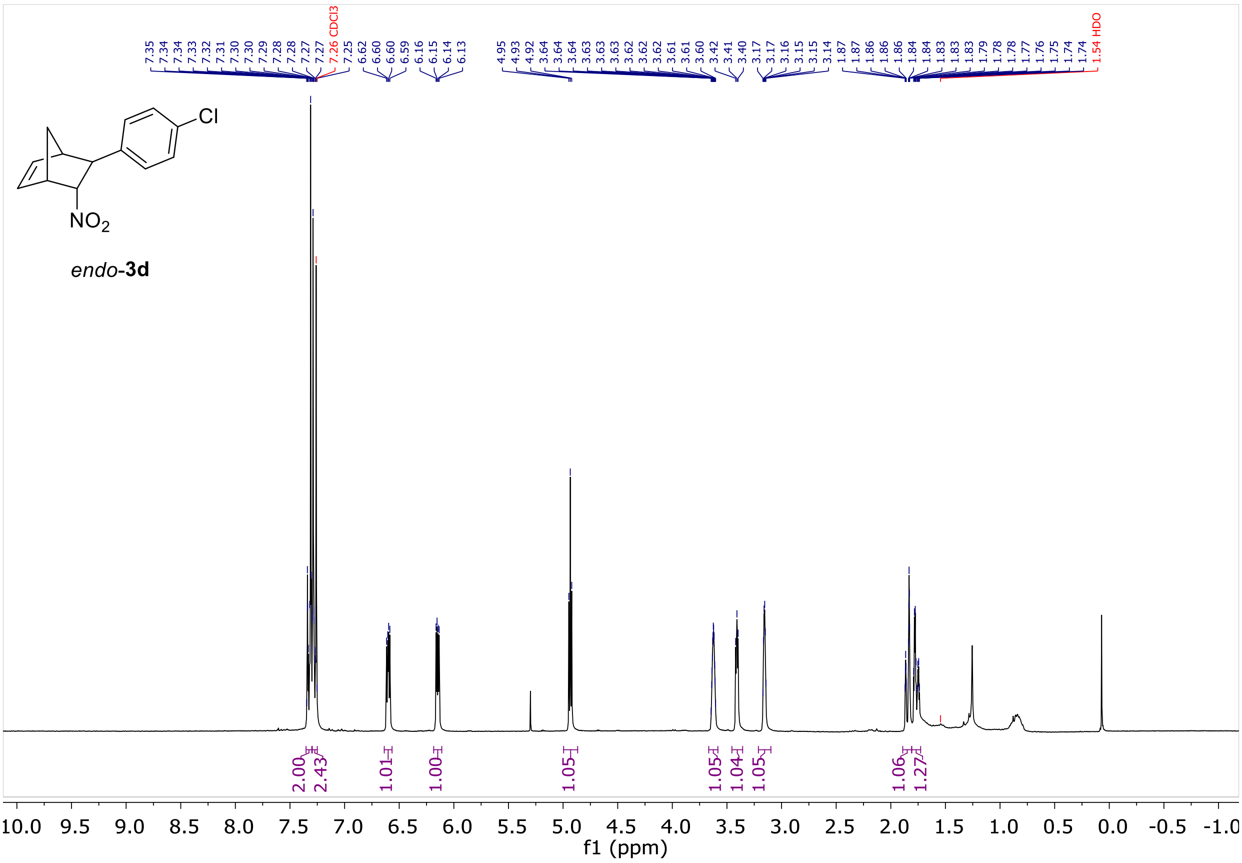

Figure S31: ^{1}H NMR of *endo*-**3d** in $CDCl_3$. The signals at ~0.85 and ~1.25 ppm correspond to grease.[2]

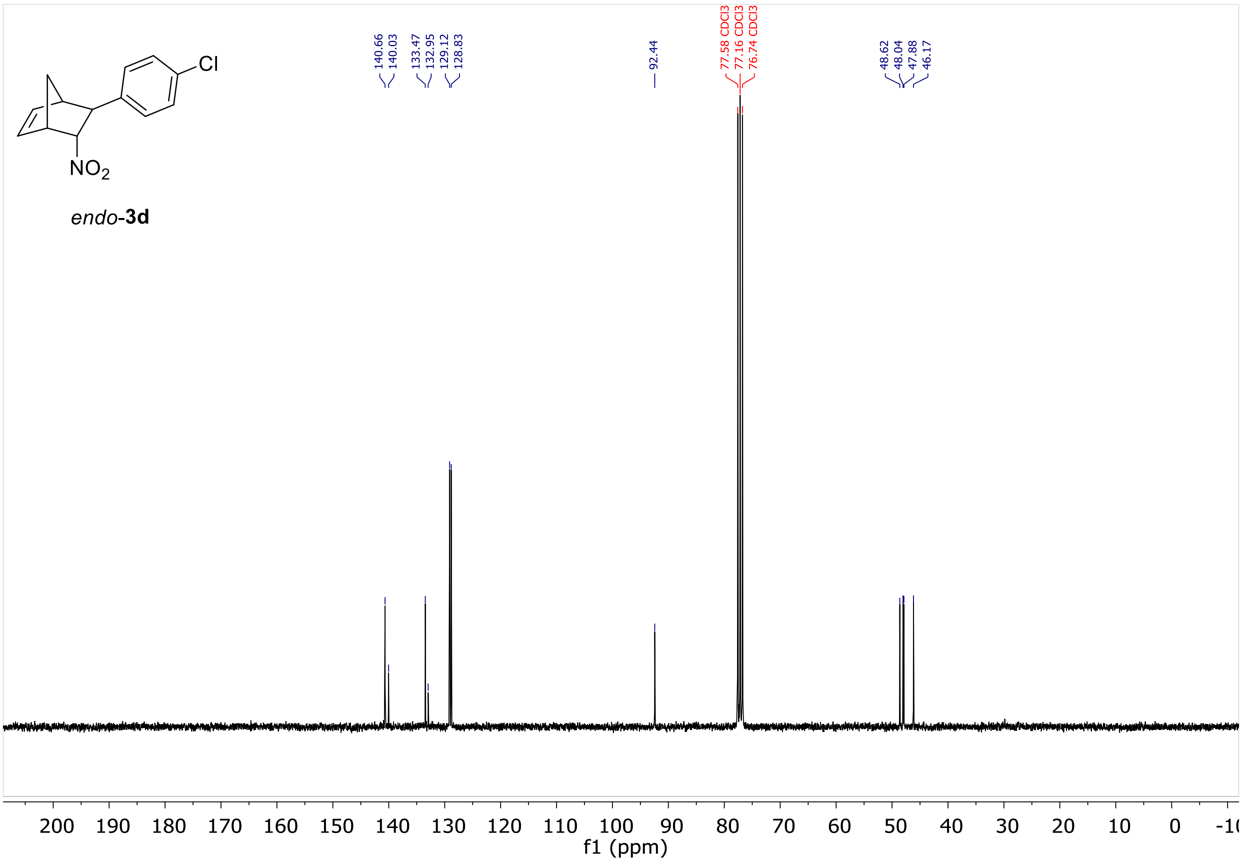

Figure S32: $^{13}C\{^{1}H\}$ NMR of *endo*-**3d** in $CDCl_3$. The signal at ~30 ppm corresponds to grease.[2]

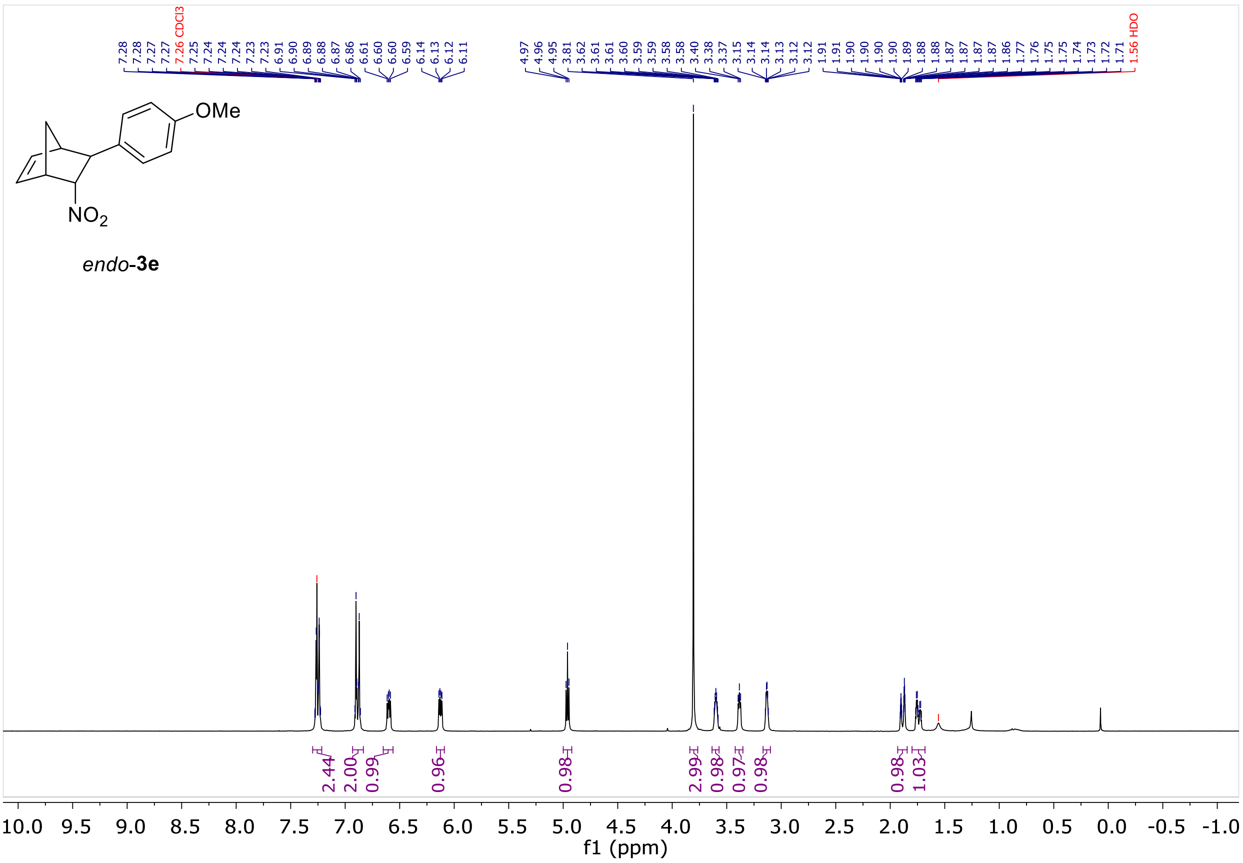

Figure S33: ^{1}H NMR of *endo*-**3e** in $CDCl_3$. The signals at ~0.85 and ~1.25 ppm correspond to grease.[2]

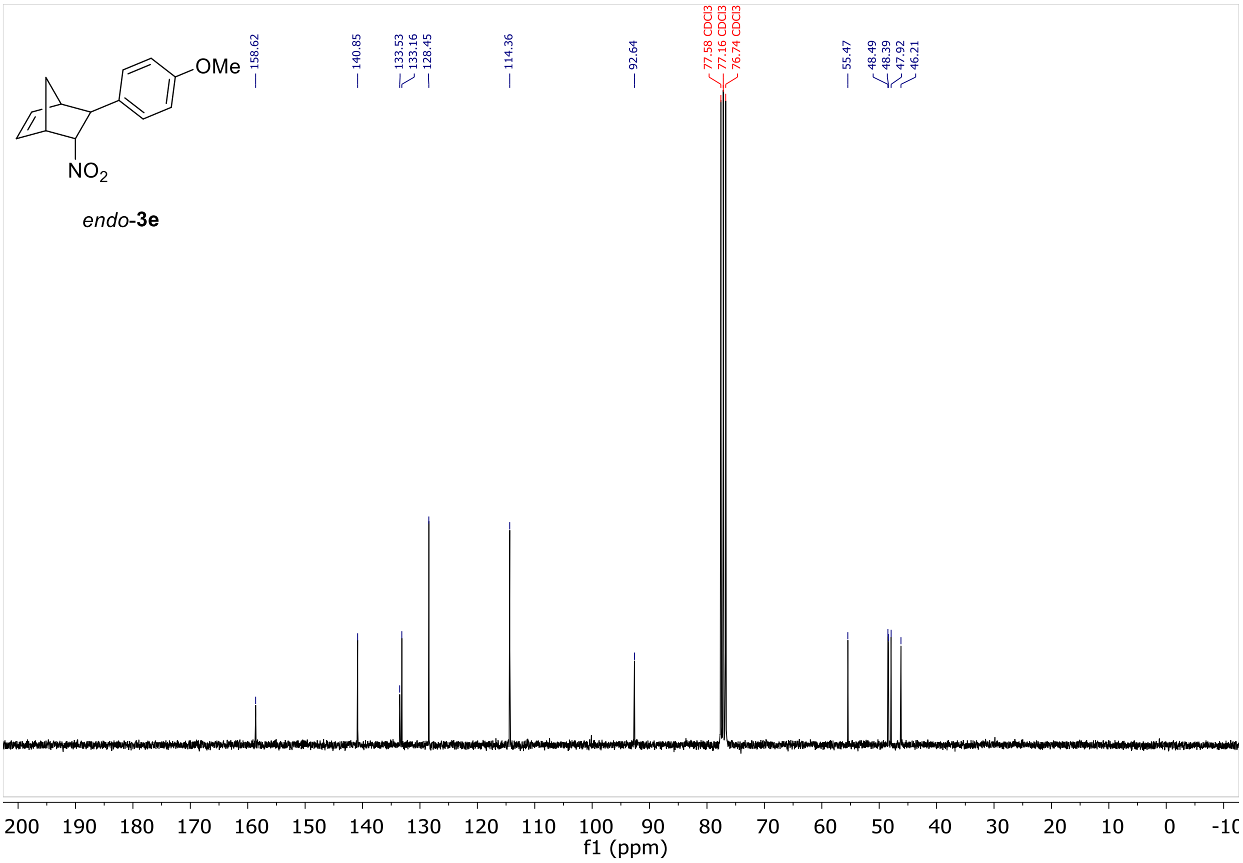

Figure S34: $^{13}C\{^1H\}$ NMR of *endo*-**3e** in $CDCl_3$.

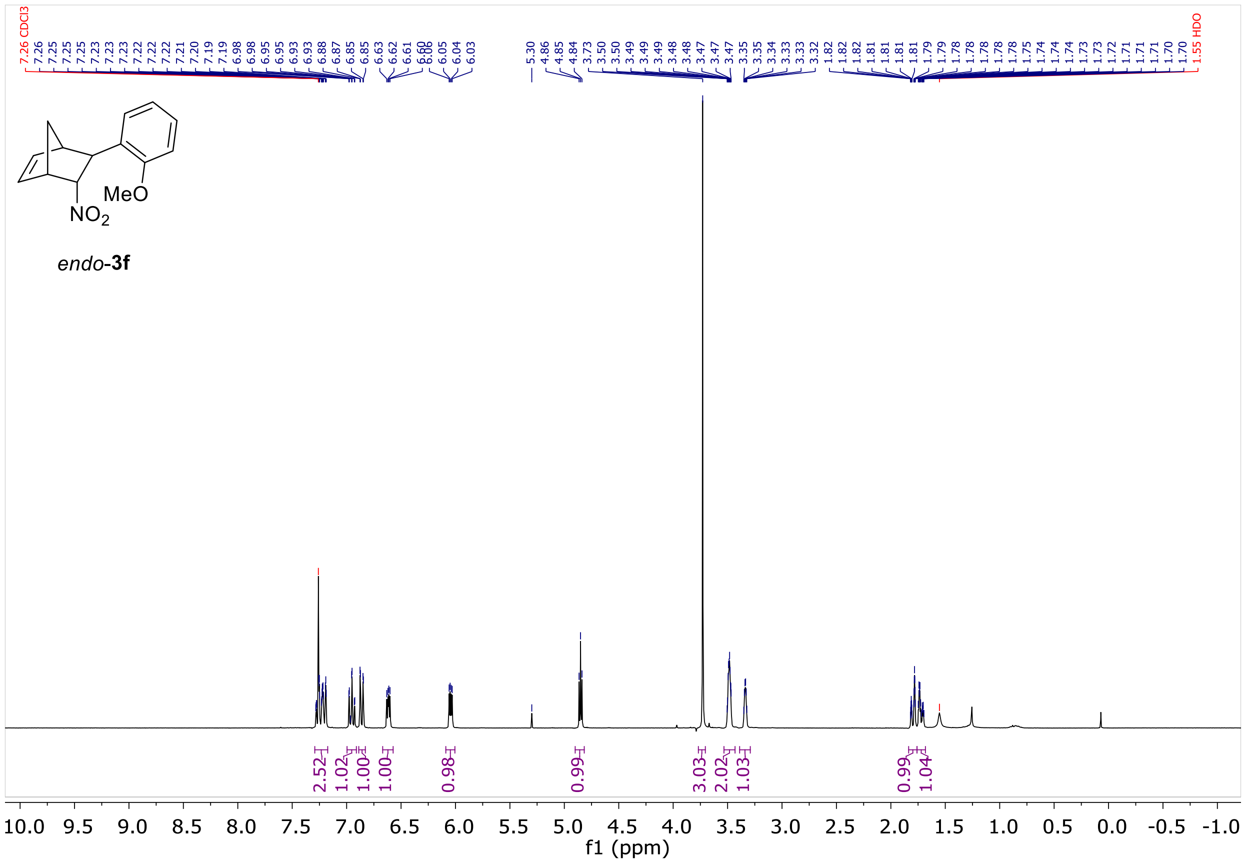

Figure S35: ^{1}H NMR of *endo*-**3f** in $CDCl_3$. The signals at ~0.85 and ~1.25 ppm correspond to grease.[2]

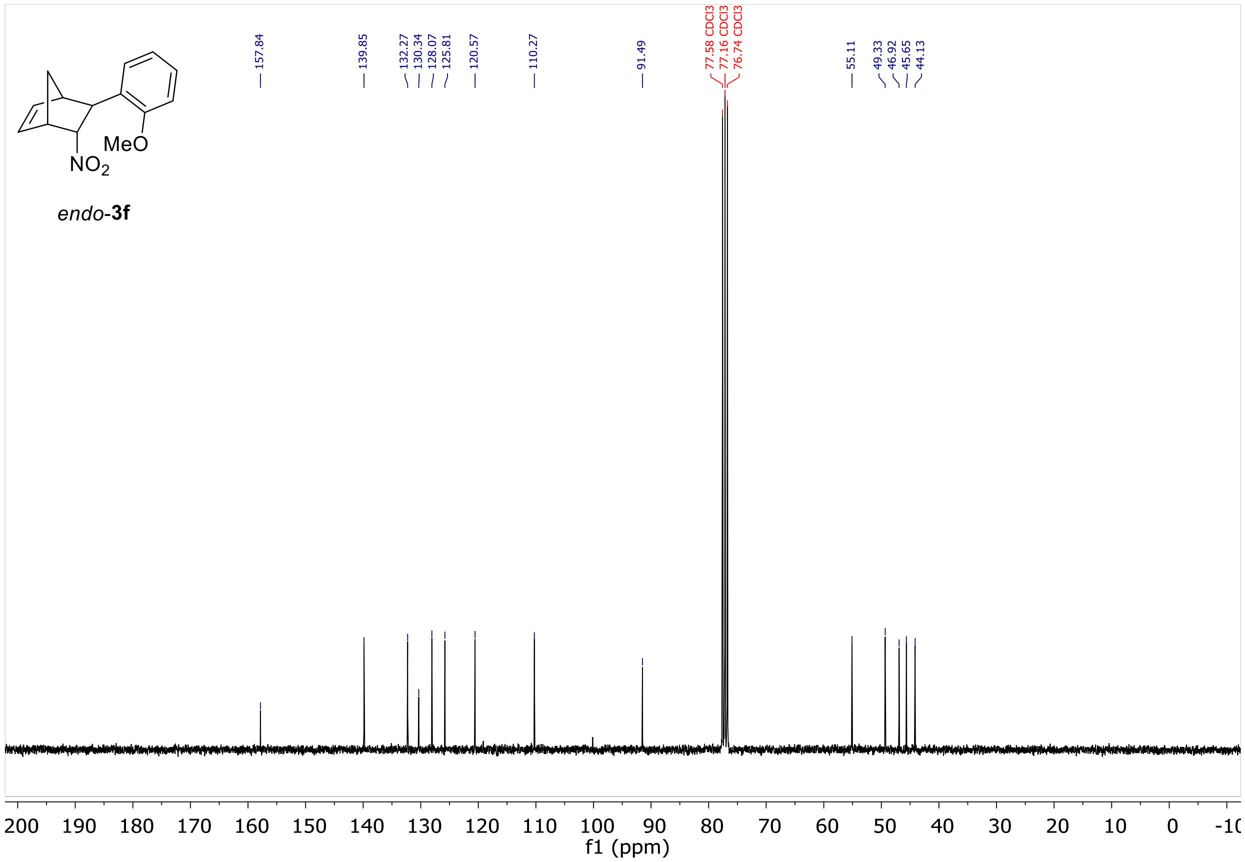

Figure S36: $^{13}C\{^{1}H\}$ NMR of *endo*-**3f** in $CDCl_3$.

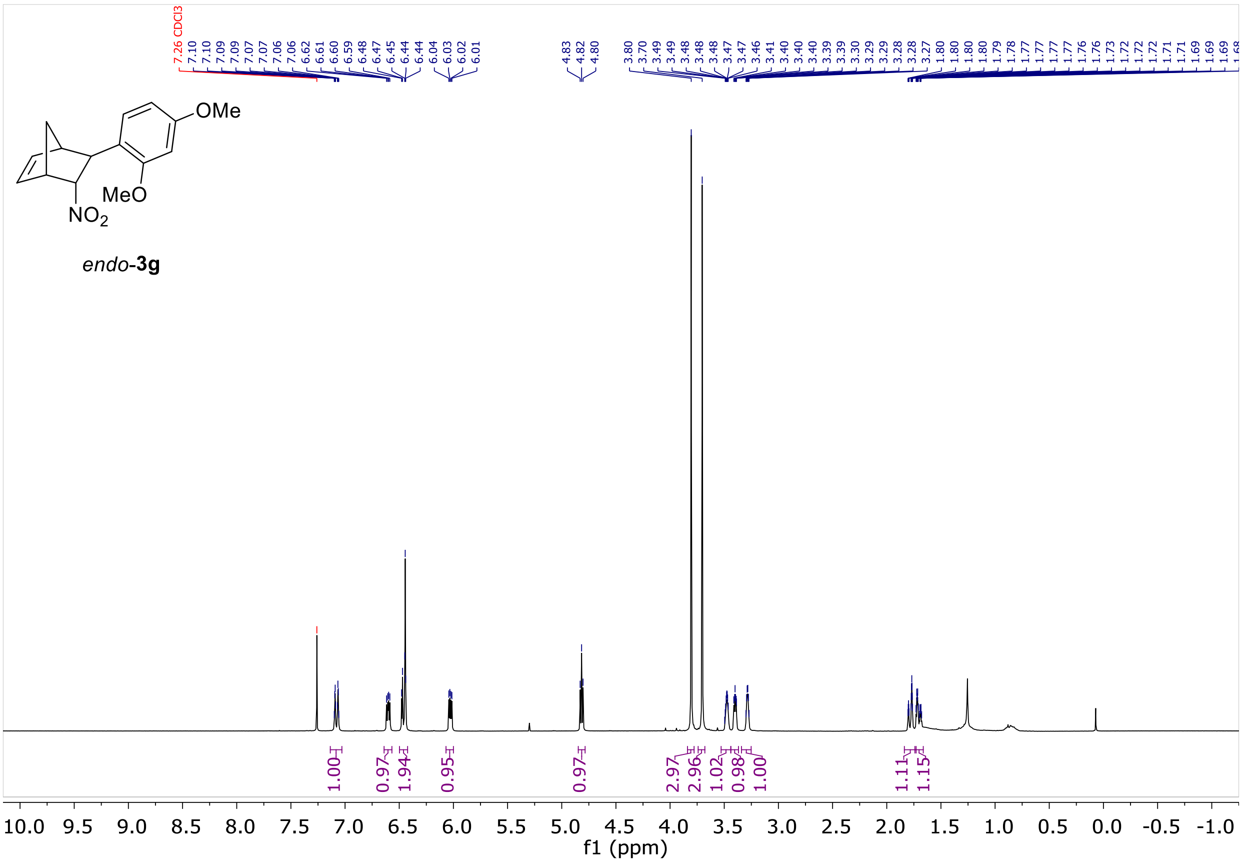

Figure S37: ^{1}H NMR of *endo*-**3g** in $CDCl_3$. The signals at ~0.85 and ~1.25 ppm correspond to grease.[2]

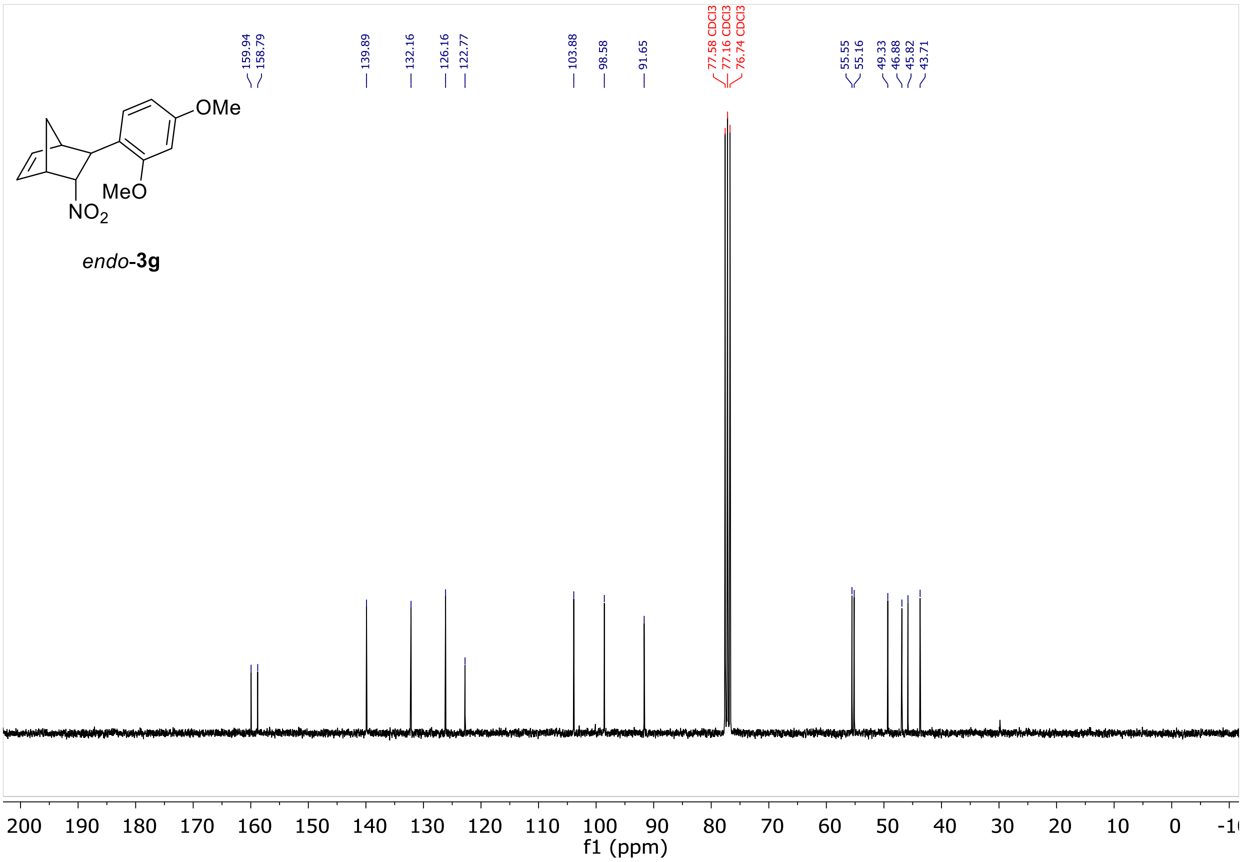

Figure S38: $^{13}C\{^{1}H\}$ NMR of *endo*-**3g** in $CDCl_3$. The signal at ~30 ppm corresponds to grease.[2]

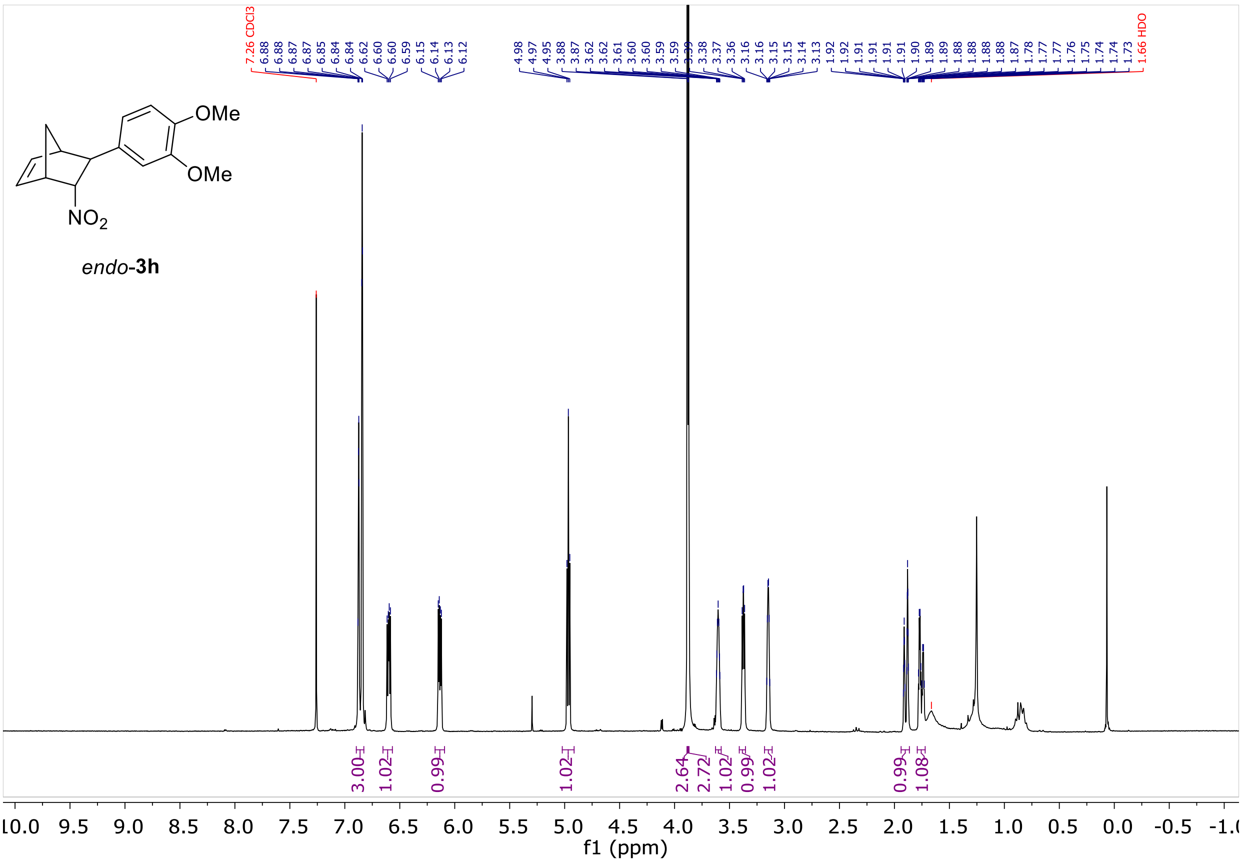

Figure S39: ^{1}H NMR of *endo*-**3h** in $CDCl_3$. The signals at ~0.85 and ~1.25 ppm correspond to grease.[2]

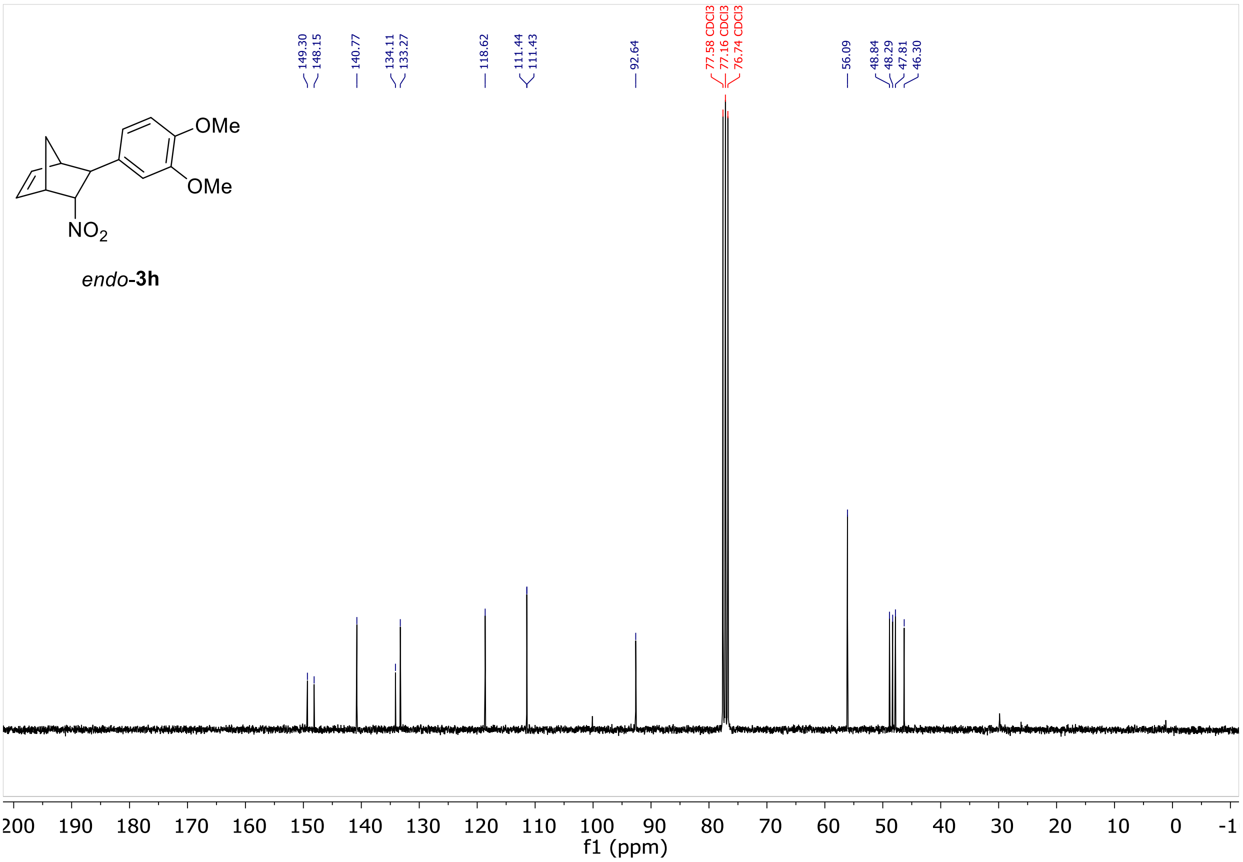

Figure S40: $^{13}C\{^1H\}$ NMR of *endo*-**3h** in $CDCl_3$. The signal at ~30 ppm corresponds to grease.[2]

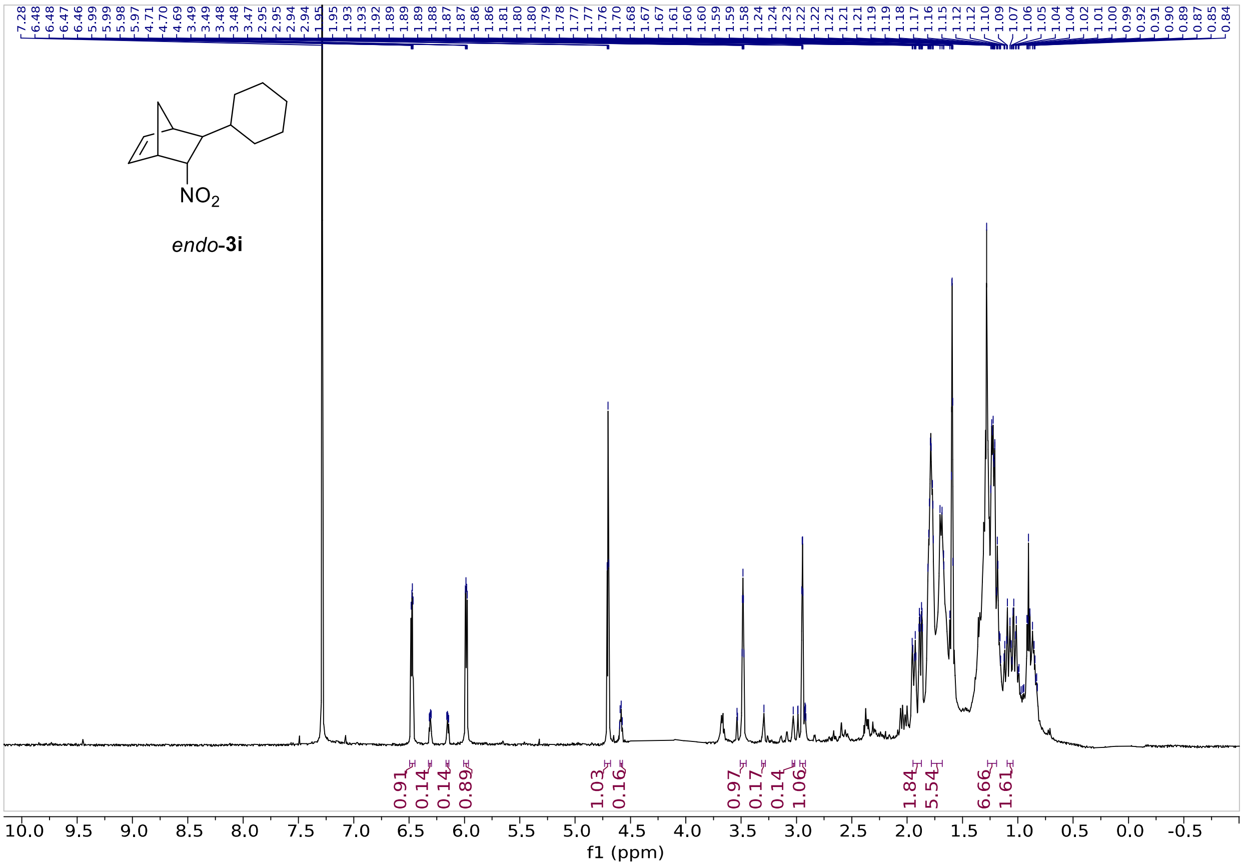

Figure S41: ^{1}H NMR of *endo*-**3i** in $CDCl_3$.

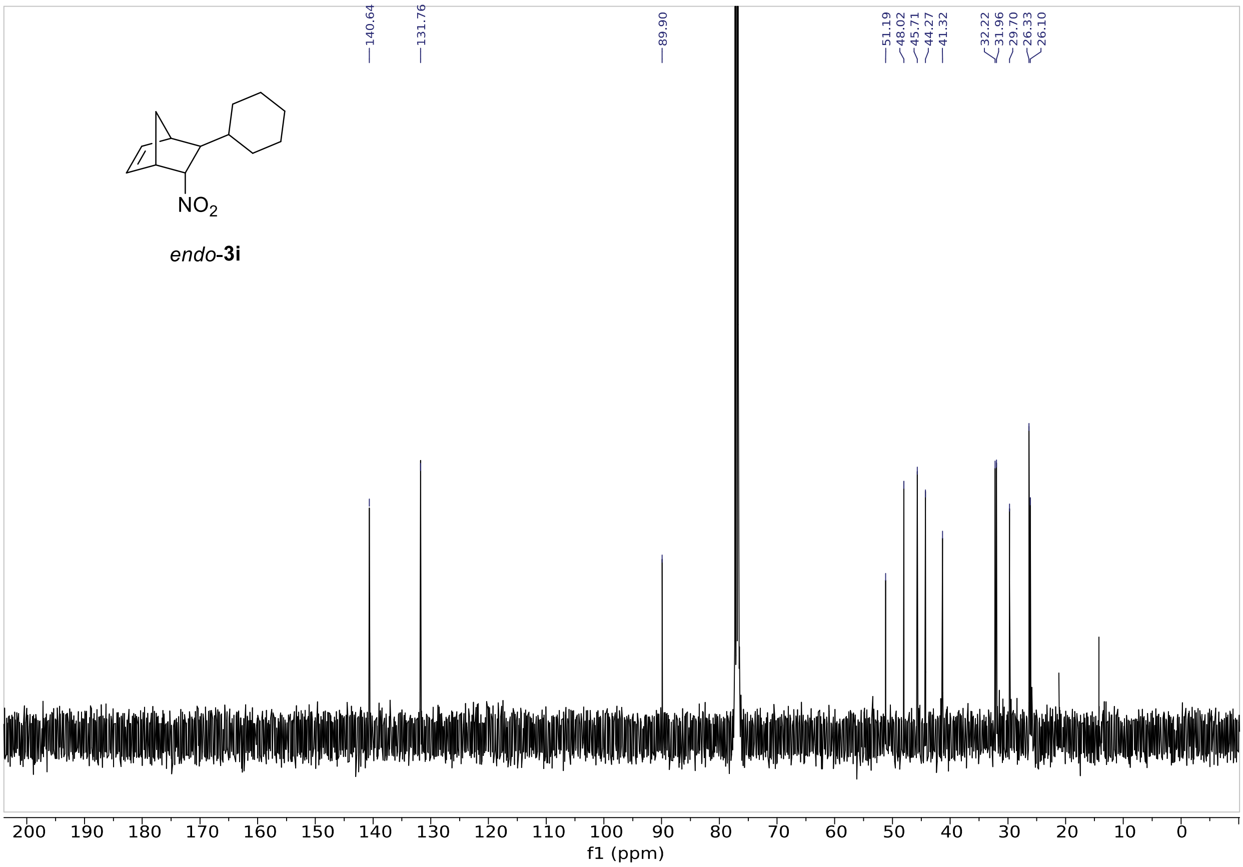

Figure S42: $^{13}C\{^{1}H\}$ NMR of *endo*-**3i** in $CDCl_3$.

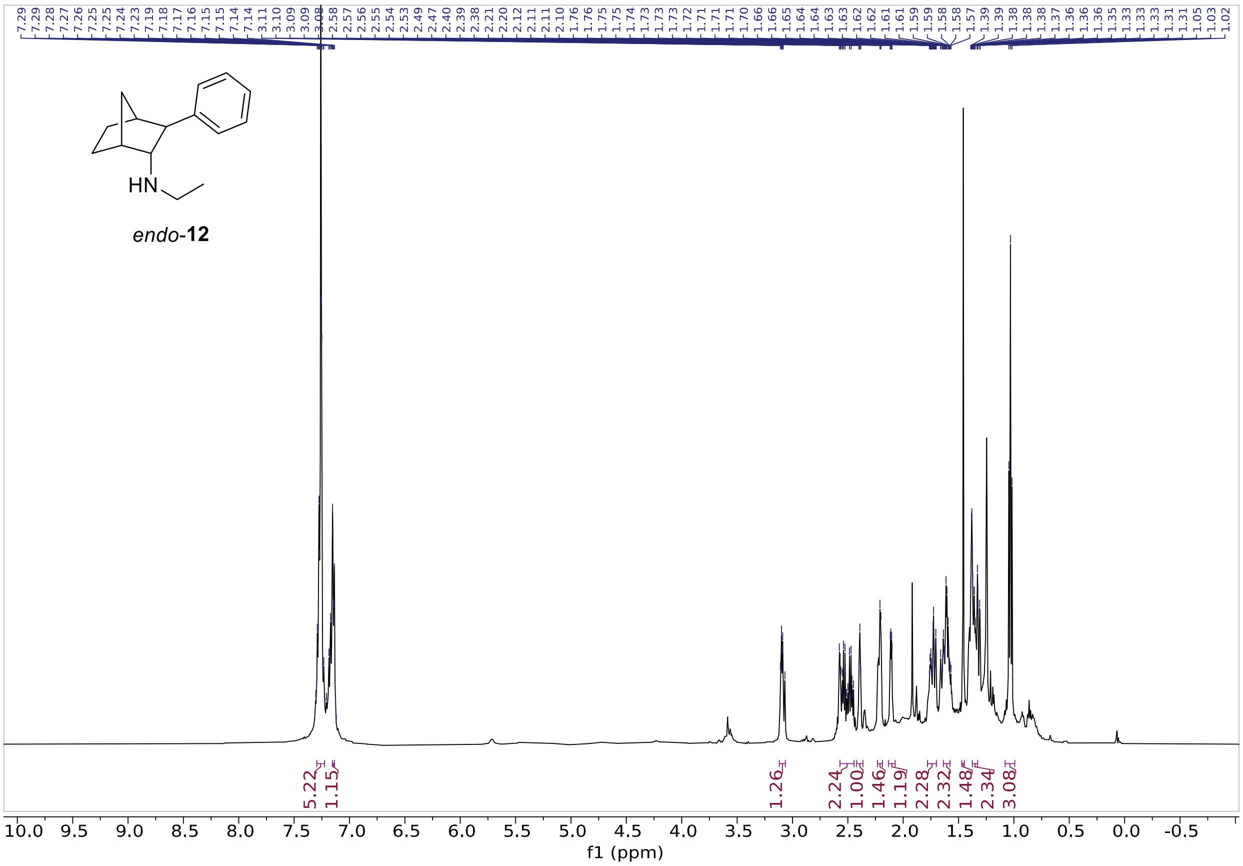

Figure S43: ^{1}H NMR of *endo*-**12** in $CDCl_3$.

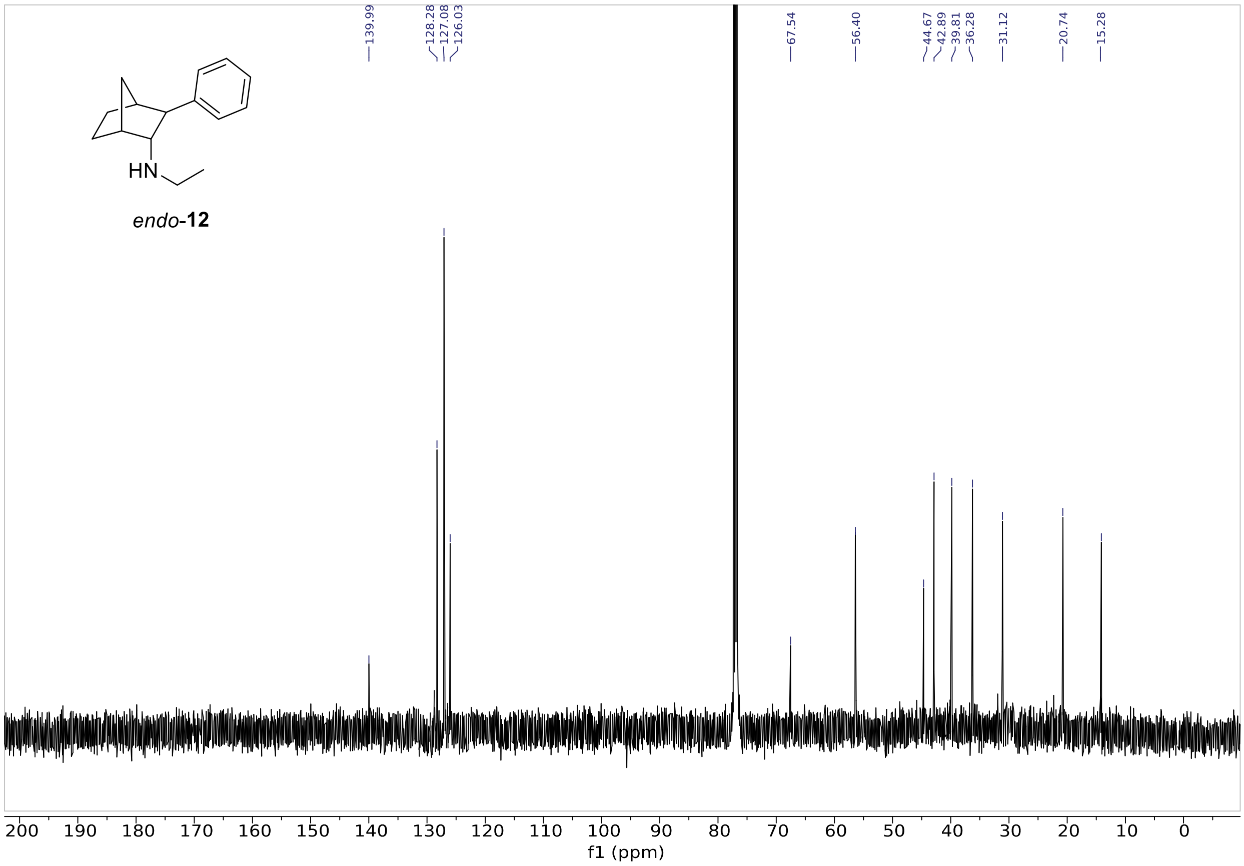

Figure S44: $^{13}C\{^{1}H\}$ NMR of *endo*-**12** in $CDCl_3$.

10. HPLC traces

10.1 5-Nitro-6-phenylbicyclo[2.2.1]hept-2-ene (3a)

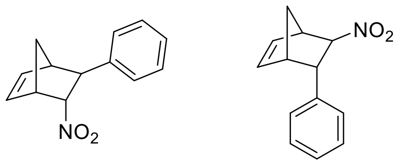

endo-**3a** *exo*-**3a**

For clarity, only one enantiomer is shown for both diastereomers. The shown configuration for *endo*-**3a** was determined to be the major enantiomer formed (section 6).

10.1.1 Racemate

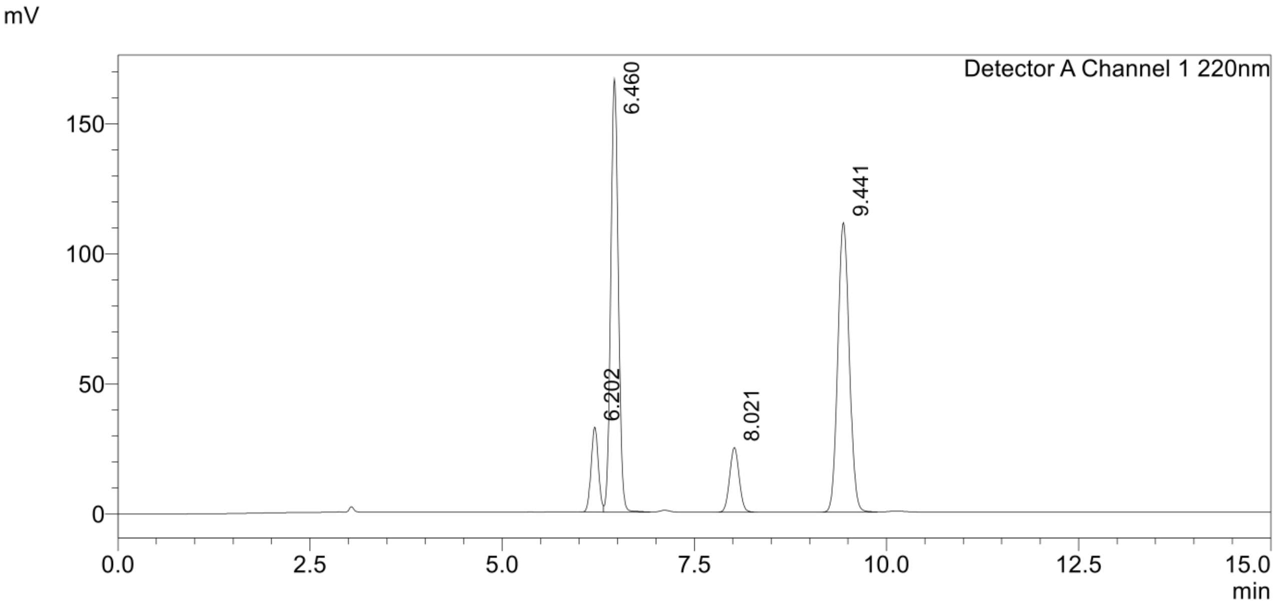

YMC CHIRAL ART Amylose-SA S-5 µm, 220 nm, 25 °C, 1 % *i*PrOH in heptane, 1.0 ml/min					
Peak #	t_R / min	Area	Height	Area / %	Compound
1	6.202	213389	32709	7.812	*exo*-**3a**
2	6.460	1149593	166411	42.083	*endo*-**3a**
3	8.021	214122	24802	7.838	*exo*-**3a**
4	9.441	1154601	111367	42.267	*endo*-**3a**
Total		2731705	335289	100.000	

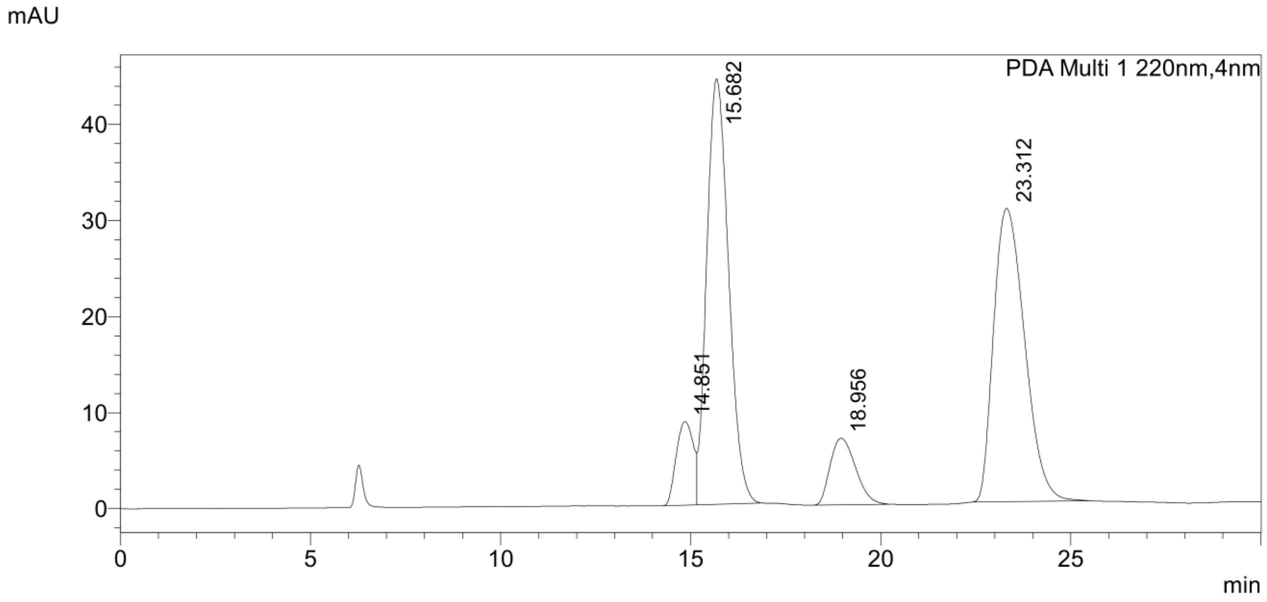

Daicel Chiralpak IA-3 (3 µm), 220 nm, 25 °C, 1 % *i*PrOH in heptane, 0.5 ml/min					
Peak #	t_R / min	Area	Height	Area / %	Compound
1	14.851	273277	8723	6.654	*exo*-**3a**
2	15.682	1776090	44312	43.245	*endo*-**3a**
3	18.956	321481	6920	7.828	*exo*-**3a**
4	23.312	1736158	30542	42.273	*endo*-**3a**
Total		4107005	90496	100.000	

10.1.2 Isolation at 50 µmol scale and 50 mM concentration with 10 mol% catalyst loading (Section 4.2.1)

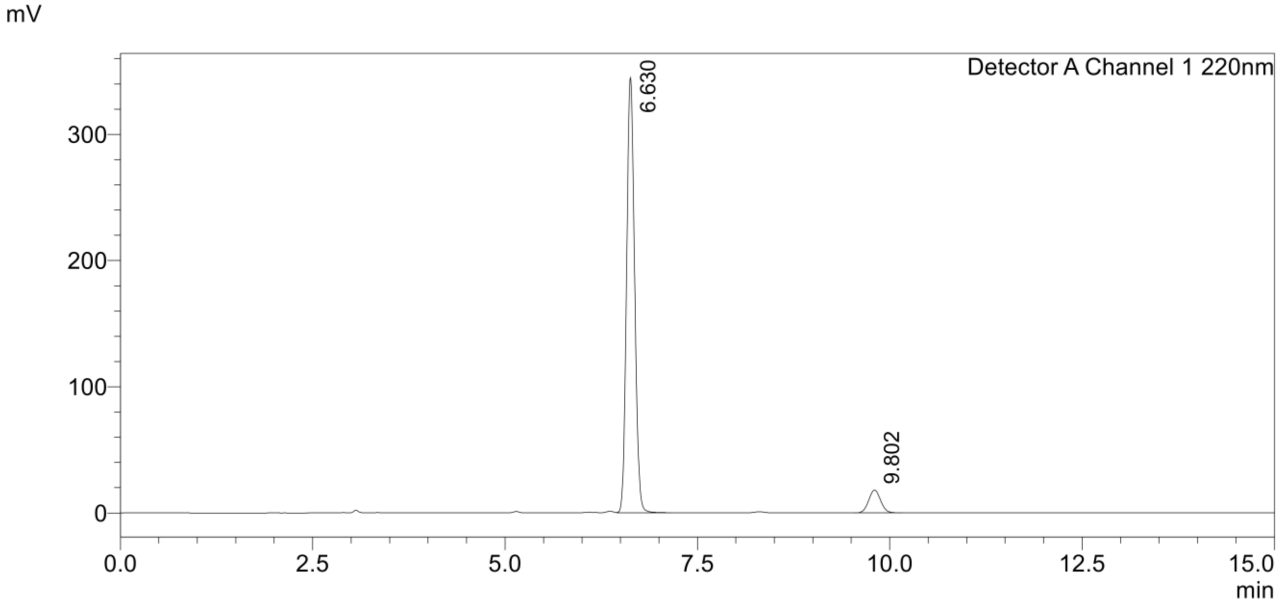

YMC CHIRAL ART Amylose-SA S-5 µm, 220 nm, 20 °C, 1 % *i*PrOH in heptane, 1.0 ml/min					
Peak #	t_R / min	Area	Height	Area / %	Compound
1	6.630	2506950	344716	92.787	*endo*-**3a**
2	9.802	194869	18003	7.213	*endo*-**3a**
Total		2701819	362720	100.000	

10.1.3 Isolation at 50 µmol scale and 12.5 mM concentration with 10 mol% catalyst loading (Section 4.2.2)

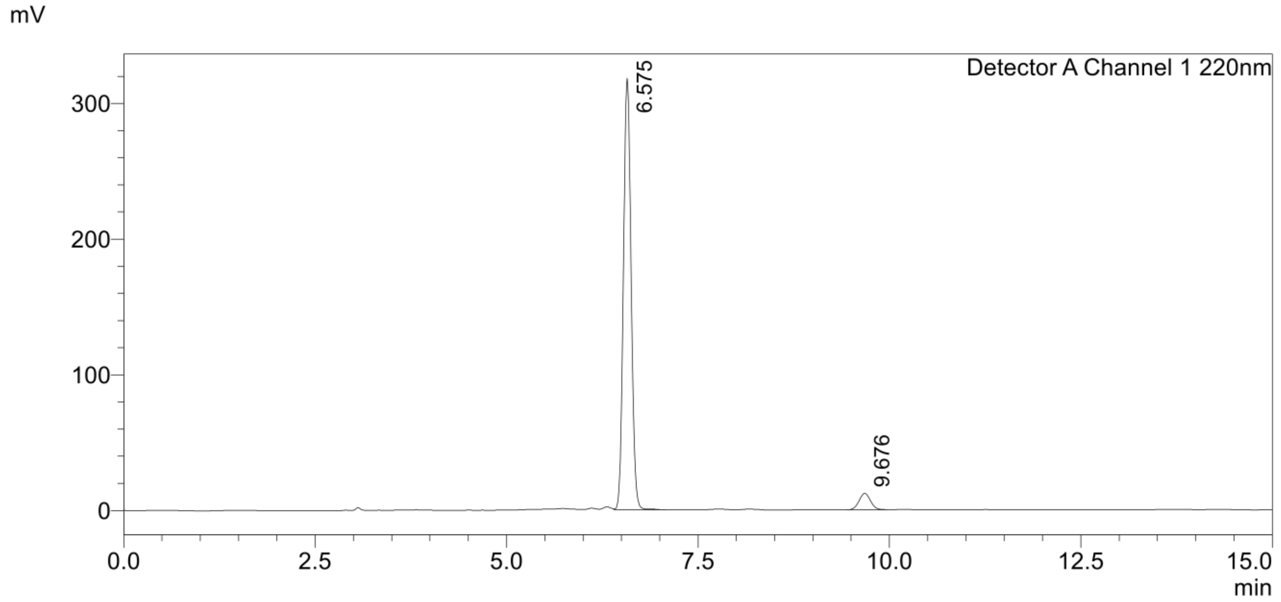

YMC CHIRAL ART Amylose-SA S-5 µm, 220 nm, 25 °C, 1 % *i*PrOH in heptane, 1.0 ml/min					
Peak #	Peak #	Area	Height	Area / %	Compound
1	1	2506950	344716	92.787	*endo*-**3a**
2	2	194869	18003	7.213	*endo*-**3a**
Total	Total	2701819	362720	100.000	

10.1.4 Isolation at 100 µmol scale and 12.5 mM concentration with 10 mol% catalyst loading (Section 4.2.3)

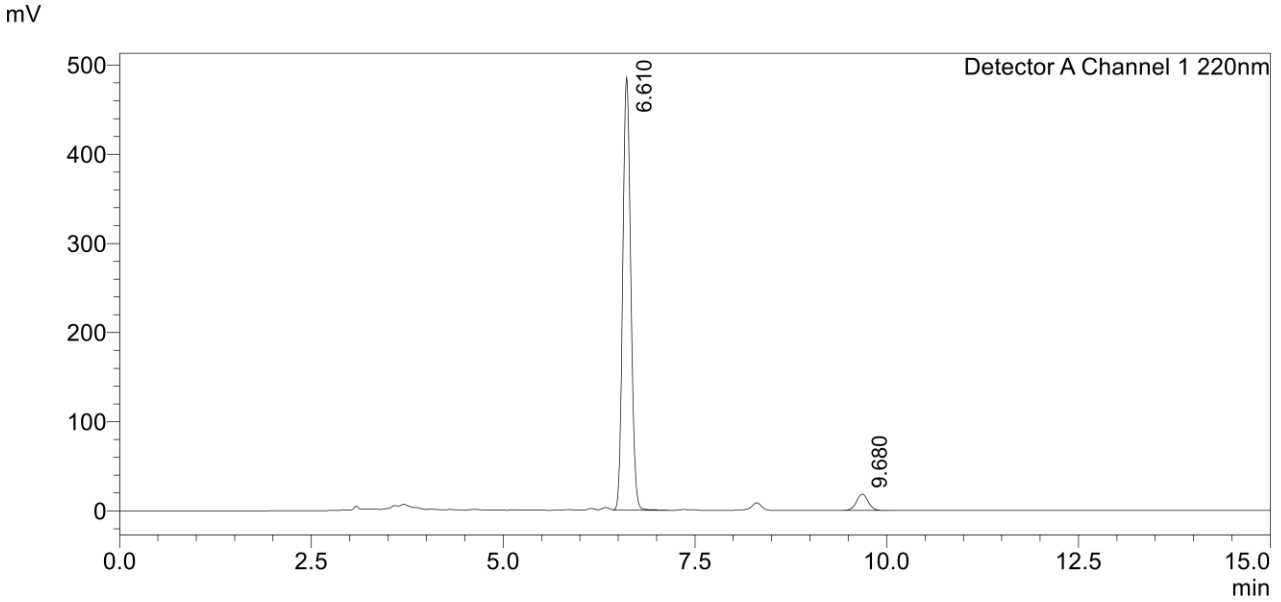

YMC CHIRAL ART Amylose-SA S-5 µm, 220 nm, 25 °C, 1 % *i*PrOH in heptane, 1.0 ml/min					
Peak #	t_R / min	Area	Height	Area / %	Compound
1	6.610	3556390	485327	94.828	*endo*-**3a**
2	9.680	193981	18293	5.172	*endo*-**3a**
Total		3750370	503620	100.000	

10.1.5 Isolation at 0.76 mmol scale and 12.5 mM concentration with 10 mol% catalyst loading (Section 4.2.4)

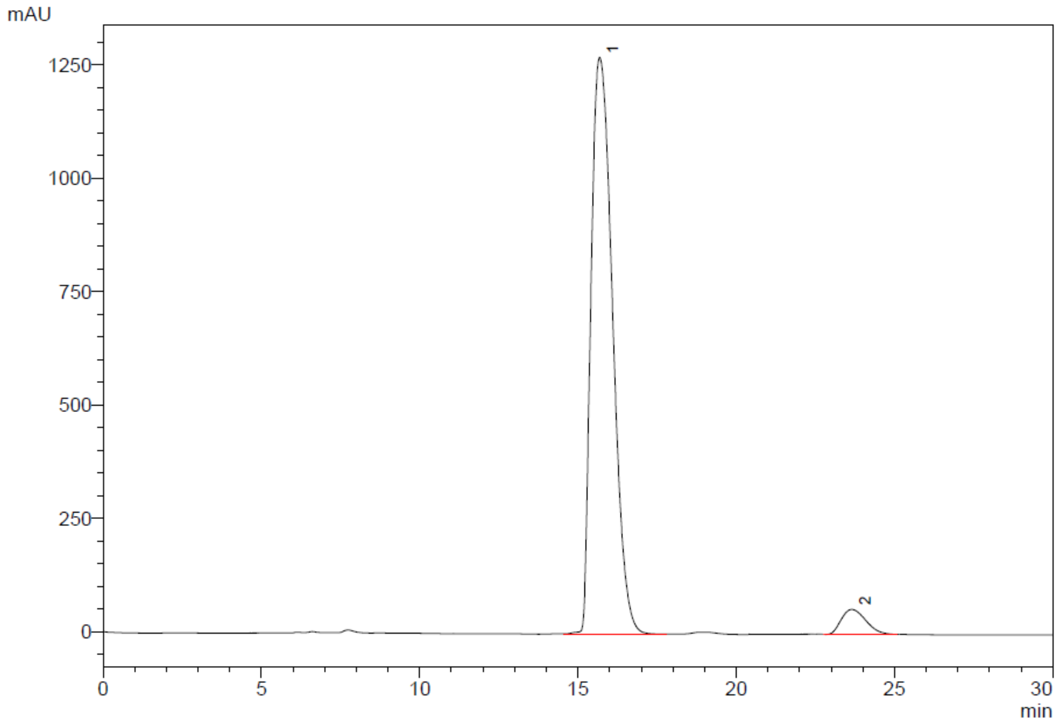

Daicel Chiralpak IA-3 (3 µm), 220 nm, 25 °C, 1 % *i*PrOH in heptane, 0.5 ml/min				
Peak #	t_R / min	Area	Area / %	Compound
1	15.791	48715516	94.542	*endo*-**3a**
2	23.895	2812393	5.458	*endo*-**3a**
Total		51527909	100.000	

10.2 5-Nitro-6-(*p*-tolyl)bicyclo[2.2.1]hept-2-ene (3b)

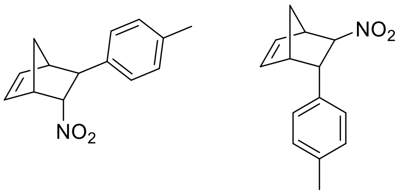

endo-**3b** *exo*-**3b**

For clarity, only one enantiomer is shown for both diastereomers.

10.2.1 Racemate

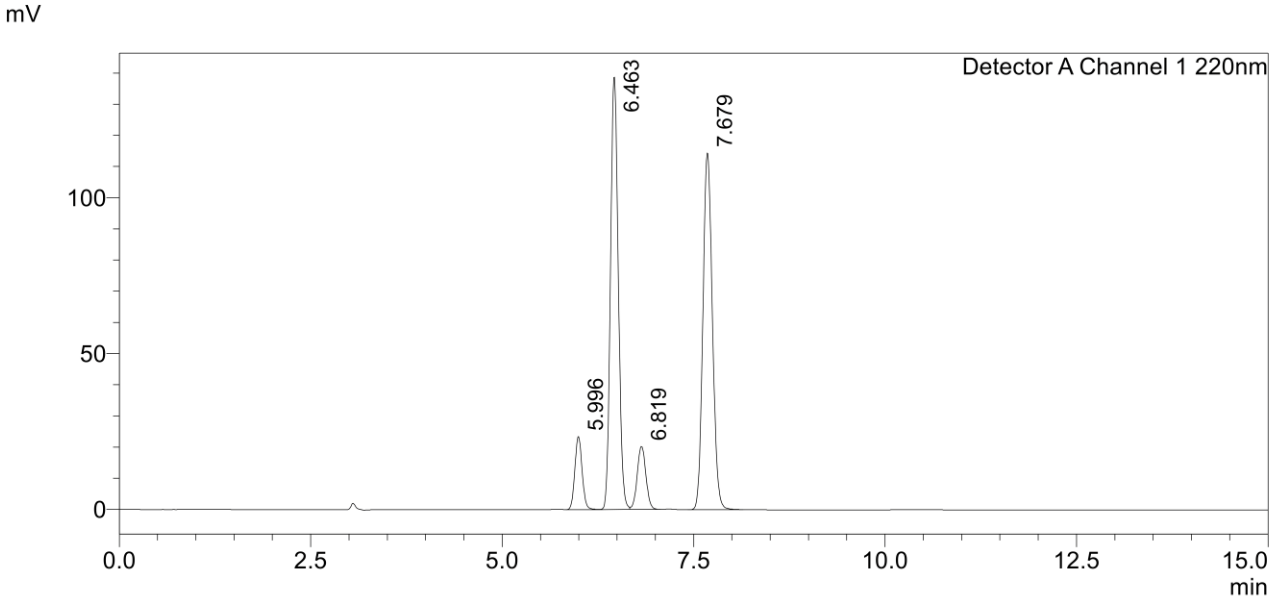

YMC CHIRAL ART Amylose-SA S-5 µm, 220 nm, 25 °C, 1 % *i*PrOH in heptane, 1.0 ml/min					
Peak #	t_R / min	Area	Height	Area / %	Compound
1	5.996	155462	23381	6.784	*exo*-**3b**
2	6.463	988801	138621	43.150	*endo*-**3b**
3	6.819	155776	20175	6.798	*exo*-**3b**
4	7.679	991509	114397	43.268	*endo*-**3b**
Total		2291548	296574	100.000	

10.2.2 Isolation at 50 µmol scale and 50 mM concentration with 10 mol% catalyst loading (Section 4.3.1)

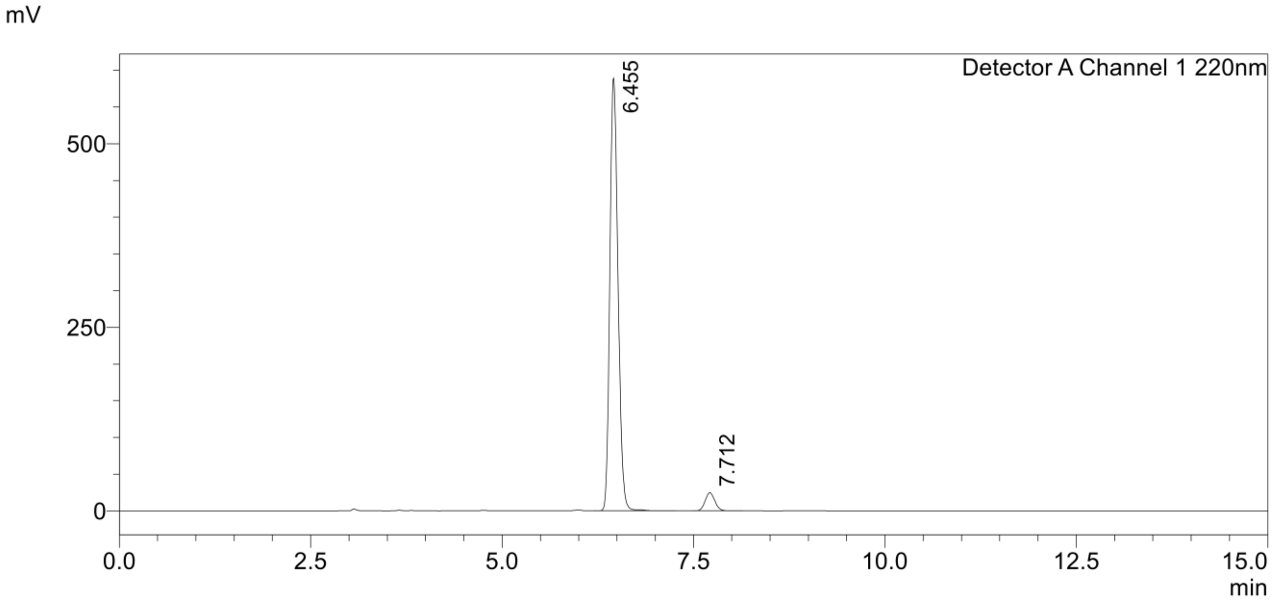

YMC CHIRAL ART Amylose-SA S-5 µm, 220 nm, 20 °C, 1 % *i*PrOH in heptane, 1.0 ml/min					
Peak #	t_R / min	Area	Height	Area / %	Compound
1	6.455	4394901	589105	95.218	*endo*-**3b**
2	7.712	220714	24629	4.782	*endo*-**3b**
Total		4615614	613734	100.000	

10.2.3 Isolation at 100 µmol scale and 50 mM concentration with 5 mol% catalyst loading (Section 4.3.2)

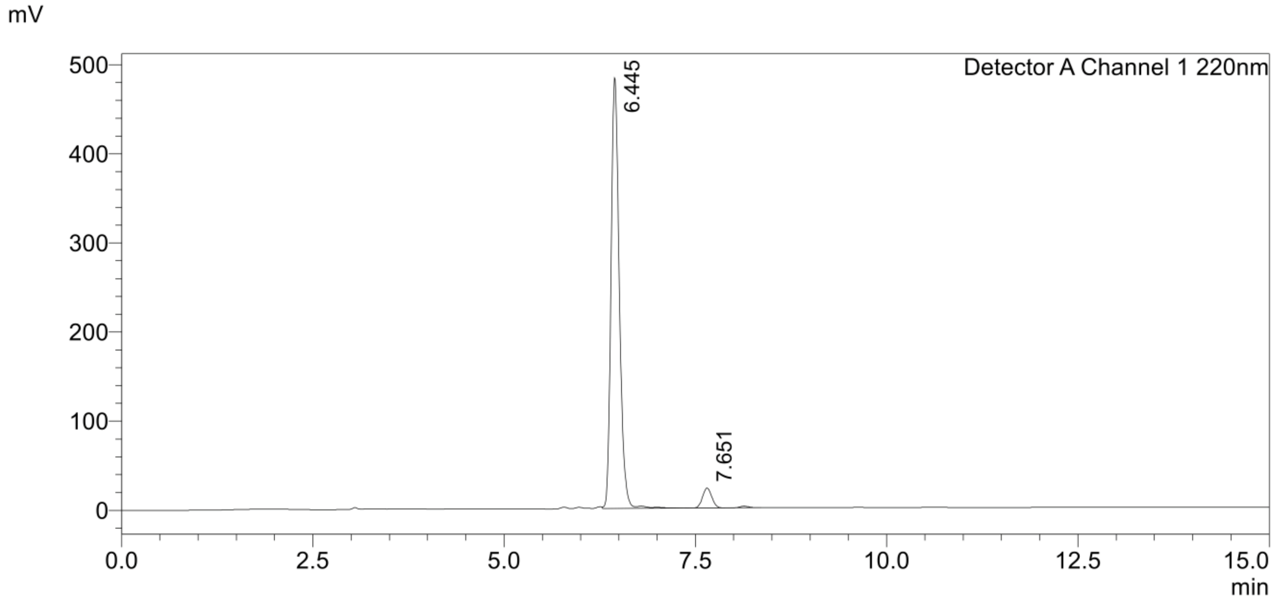

YMC CHIRAL ART Amylose-SA S-5 µm, 220 nm, 25 °C, 1 % *i*PrOH in heptane, 1.0 ml/min					
Peak #	t_R / min	Area	Height	Area / %	Compound
1	6.445	3668559	483154	95.064	*endo*-**3b**
2	7.651	190469	22378	4.936	*endo*-**3b**
Total		3859028	505531	100.000	

10.3 5-Nitro-6-(4-fluorophenyl)bicyclo[2.2.1]hept-2-ene (3c)

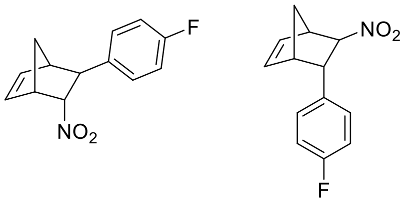

endo-**3c** *exo*-**3c**

For clarity, only one enantiomer is shown for both diastereomers.

10.3.1 Racemate

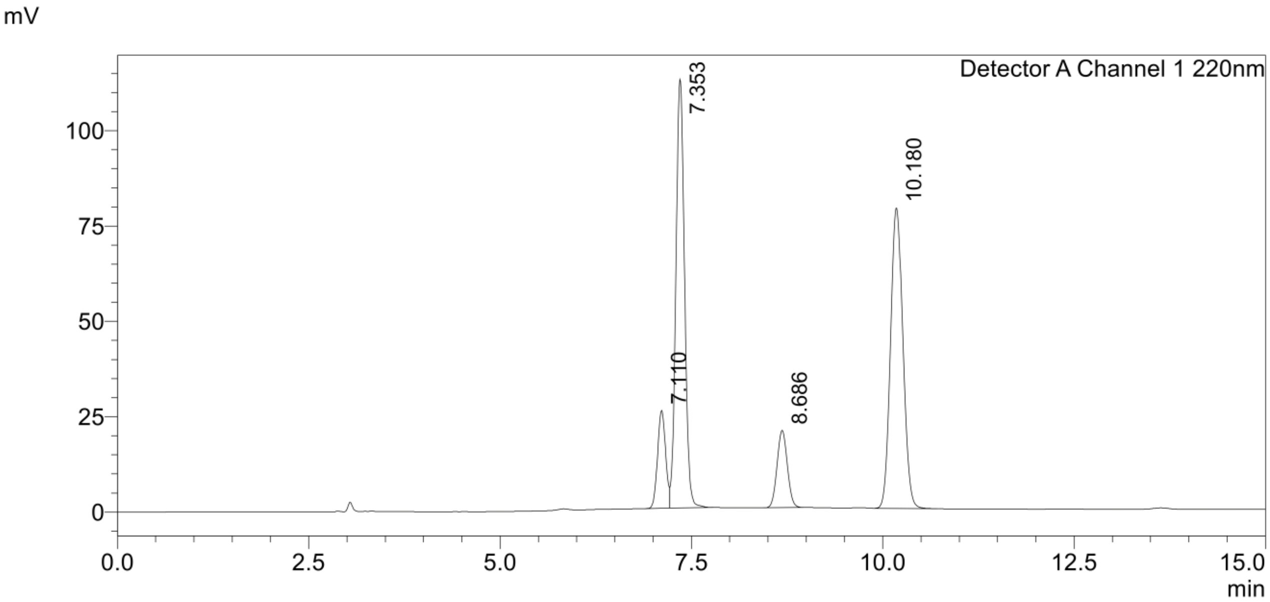

YMC CHIRAL ART Amylose-SA S-5 µm, 220 nm, 25 °C, 1 % *i*PrOH in heptane, 1.0 ml/min					
Peak #	t_R / min	Area	Height	Area / %	Compound
1	7.110	191261	25664	8.911	*exo*-**3c**
2	7.353	883107	112383	41.144	*endo*-**3c**
3	8.686	189797	20257	8.843	*exo*-**3c**
4	10.180	882195	78761	41.102	*endo*-**3c**
Total		2146360	237064	100.000	

10.3.2 Isolation at 50 µmol scale and 50 mM concentration with 10 mol% catalyst loading (Section 4.4.1)

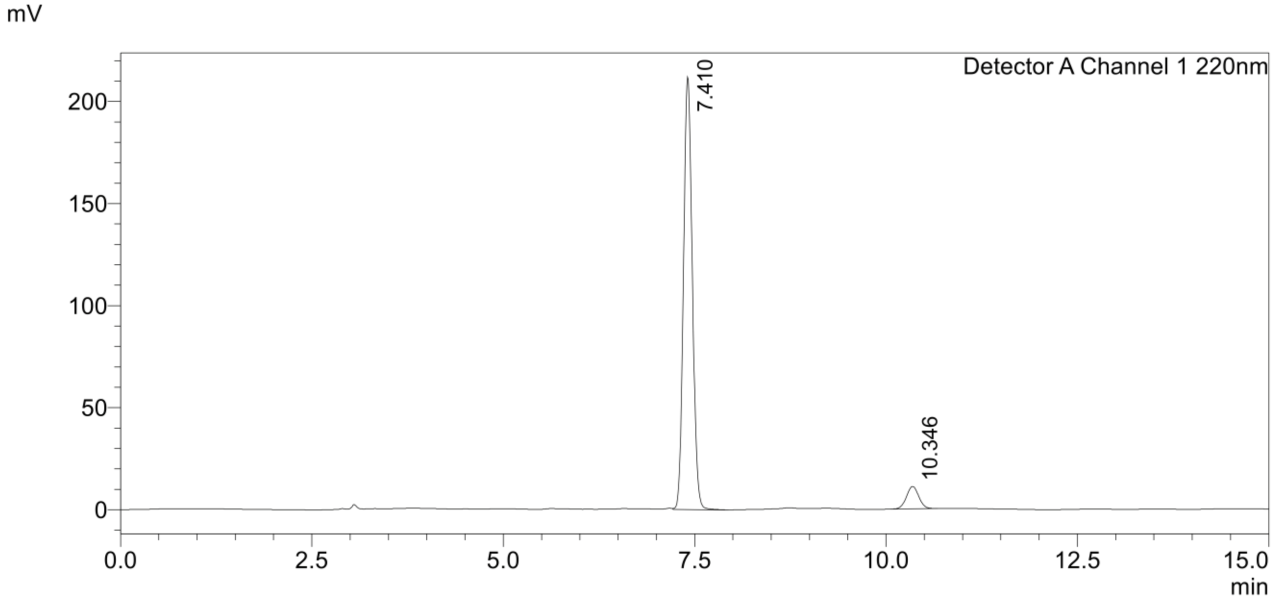

YMC CHIRAL ART Amylose-SA S-5 µm, 220 nm, 25 °C, 1 % *i*PrOH in heptane, 1.0 ml/min					
Peak #	t_R / min	Area	Height	Area / %	Compound
1	7.410	1694669	211899	93.240	*endo*-**3c**
2	10.346	122869	10949	6.760	*endo*-**3c**
Total		1817537	222848	100.000	

10.3.3 Isolation at 100 µmol scale and 50 mM concentration with 10 mol% catalyst loading (Section 4.4.2)

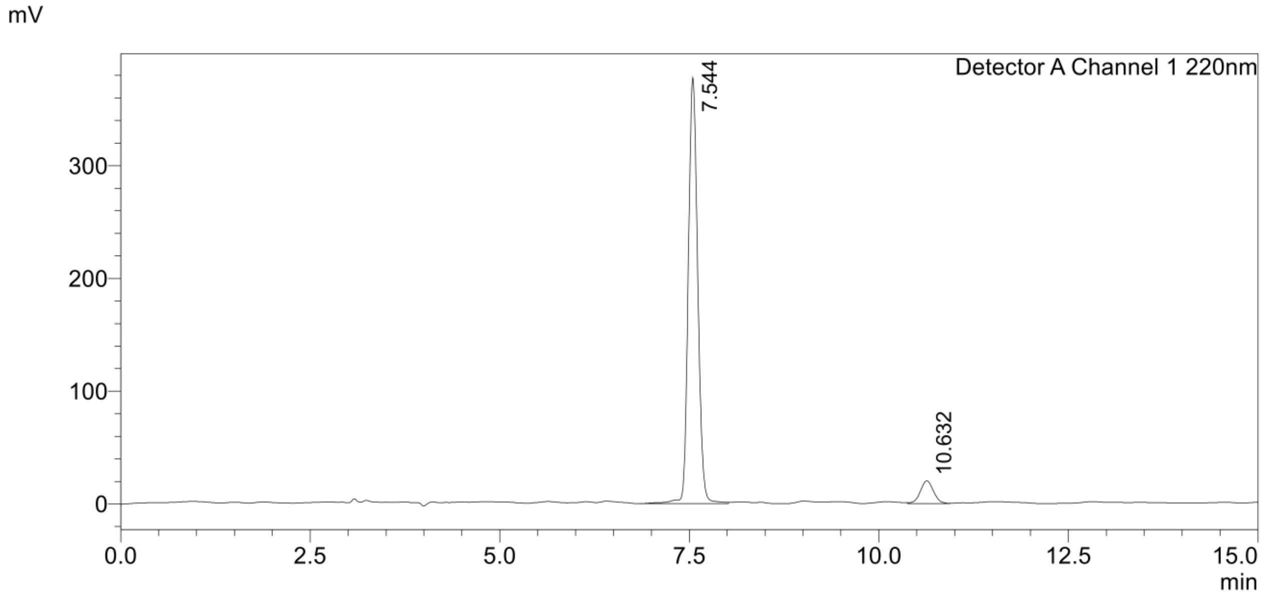

YMC CHIRAL ART Amylose-SA S-5 µm, 220 nm, 25 °C, 1 % *i*PrOH in heptane, 1.0 ml/min					
Peak #	t_R / min	Area	Height	Area / %	Compound
1	7.544	3242589	377721	92.909	*endo*-**3c**
2	10.632	247467	20189	7.091	*endo*-**3c**
Total		3490057	397911	100.000	

10.4 5-Nitro-6-(4-chlorophenyl)bicyclo[2.2.1]hept-2-ene (3d)

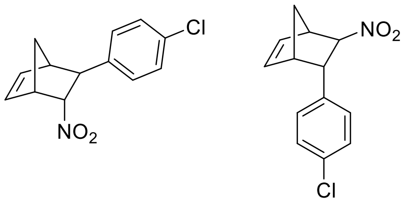

endo-**3d** *exo*-**3d**

For clarity, only one enantiomer is shown for both diastereomers.

10.4.1 Racemate

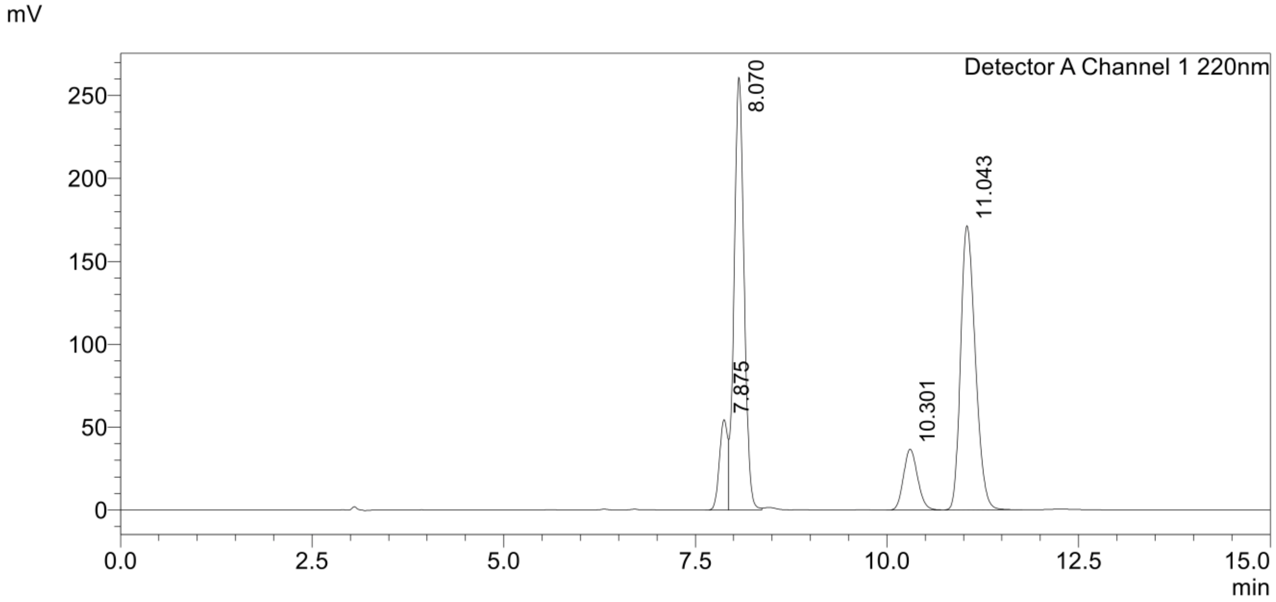

YMC CHIRAL ART Amylose-SA S-5 µm, 220 nm, 25 °C, 1 % *i*PrOH in heptane, 1.0 ml/min					
Peak #	t_R / min	Area	Height	Area / %	Compound
1	7.875	412080	54425	7.438	*exo*-**3d**
2	8.070	2361515	260786	42.624	*endo*-**3d**
3	10.301	466375	36728	8.418	*exo*-**3d**
4	11.043	2300435	171481	41.521	*endo*-**3d**
Total		5540404	523419	100.000	

10.4.2 Isolation at 50 µmol scale and 50 mM concentration with 10 mol% catalyst loading (Section 4.5.1)

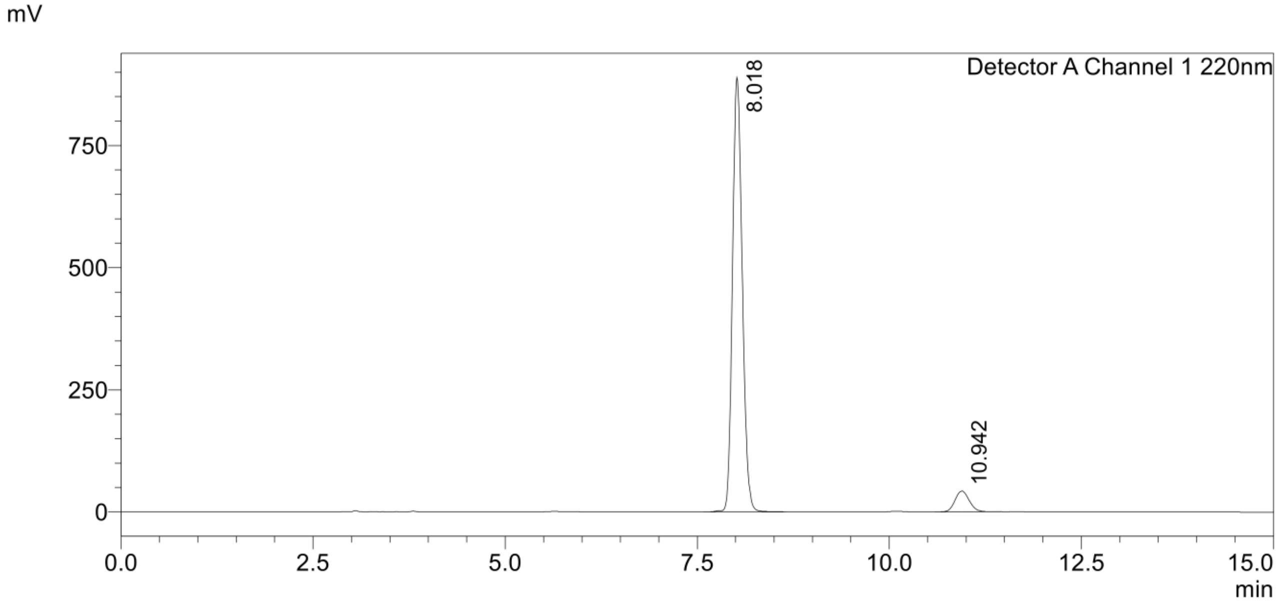

YMC CHIRAL ART Amylose-SA S-5 µm, 220 nm, 25 °C, 1 % *i*PrOH in heptane, 1.0 ml/min					
Peak #	t_R / min	Area	Height	Area / %	Compound
1	8.018	7928110	888900	93.543	*endo*-**3d**
2	10.942	547224	42720	6.457	*endo*-**3d**
Total		8475334	931620	100.000	

10.4.3 Isolation at 100 µmol scale and 50 mM concentration with 10 mol% catalyst loading (Section 4.5.2)

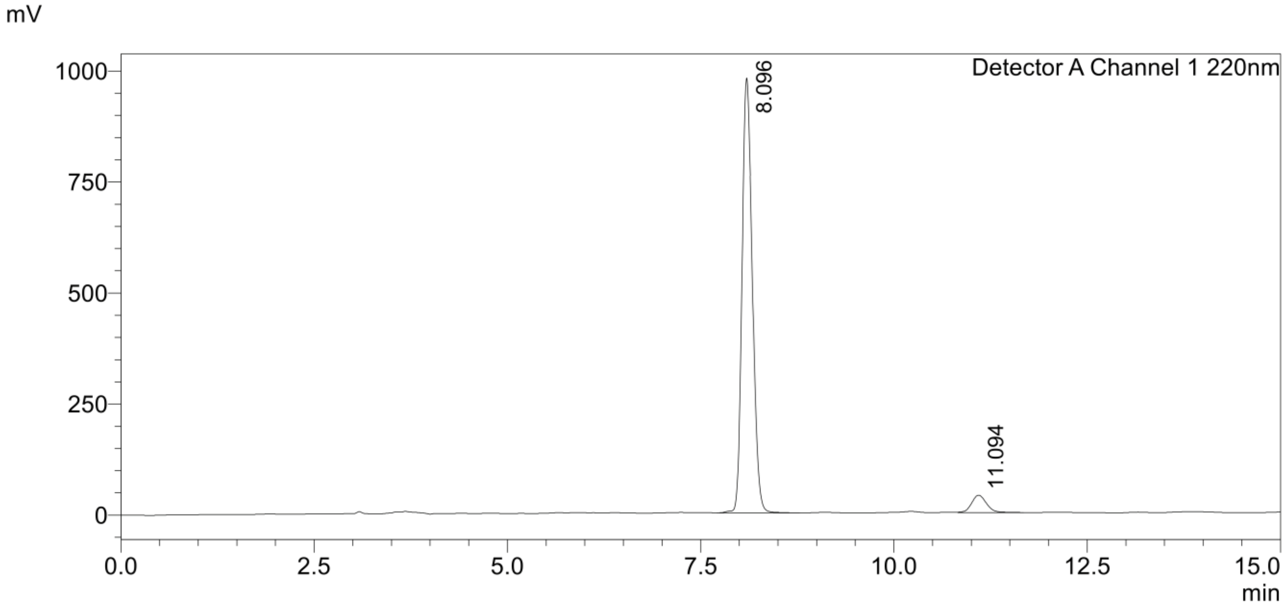

YMC CHIRAL ART Amylose-SA S-5 µm, 220 nm, 25 °C, 1 % *i*PrOH in heptane, 1.0 ml/min					
Peak #	t_R / min	Area	Height	Area / %	Compound
1	8.096	9045632	978761	94.451	*endo*-**3d**
2	11.094	531460	39186	5.549	*endo*-**3d**
Total		9577093	1017947	100.000	

10.5 5-Nitro-6-(4-methoxyphenyl)bicyclo[2.2.1]hept-2-ene (3e)

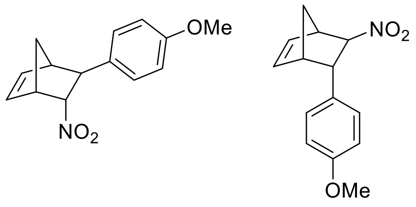

endo-**3e** *exo*-**3e**

For clarity, only one enantiomer is shown for both diastereomers.

10.5.1 Racemate

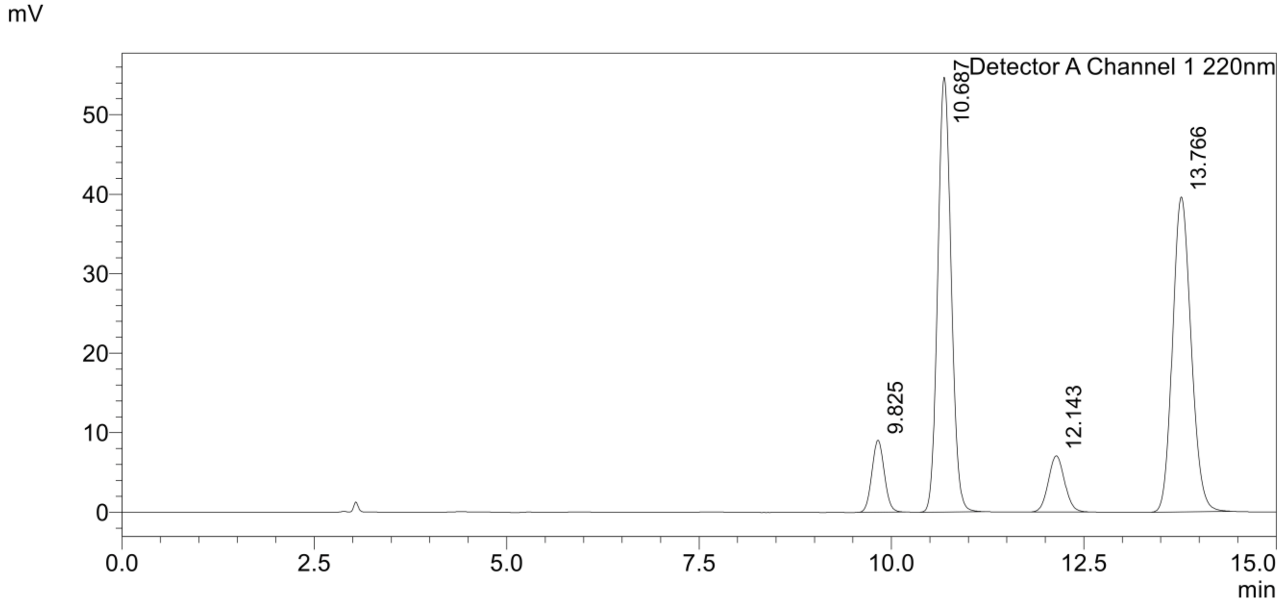

YMC CHIRAL ART Amylose-SA S-5 µm, 220 nm, 25 °C, 1 % *i*PrOH in heptane, 1.0 ml/min					
Peak #	t_R / min	Area	Height	Area / %	Compound
1	9.825	102347	9067	6.660	*exo*-**3e**
2	10.687	666594	54661	43.378	*endo*-**3e**
3	12.143	103103	7052	6.709	*exo*-**3e**
4	13.766	664675	39629	43.253	*endo*-**3e**
Total		1536720	110409	100.000	

10.5.2 Isolation at 50 µmol scale and 50 mM concentration with 10 mol% catalyst loading (Section 4.6.1)

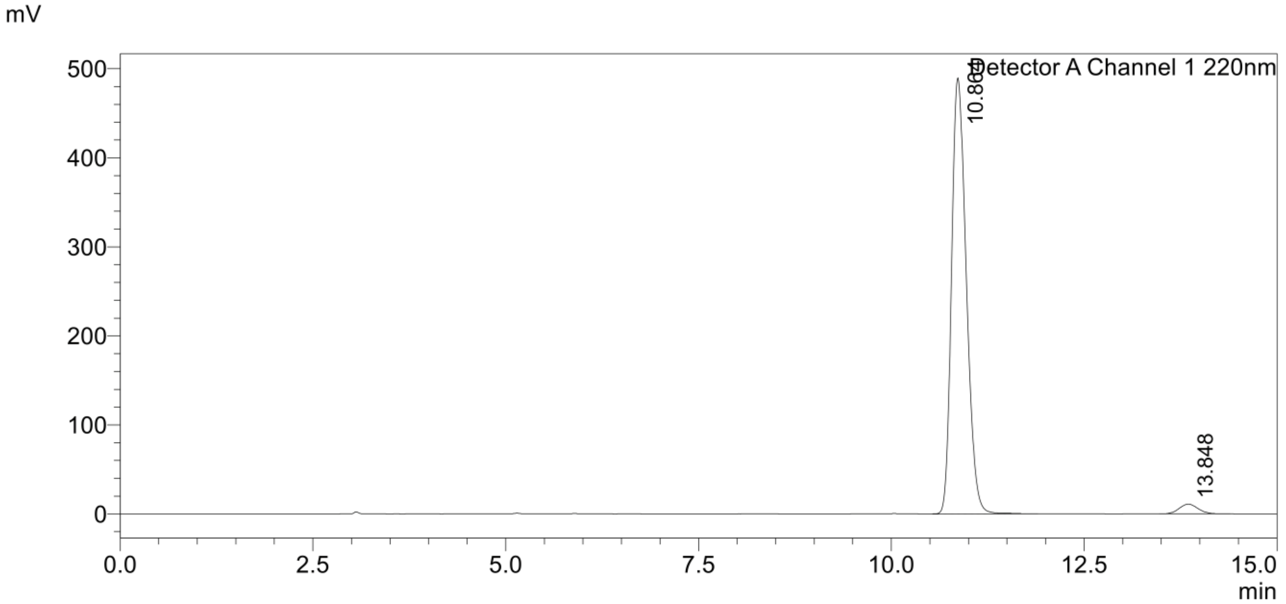

YMC CHIRAL ART Amylose-SA S-5 µm, 220 nm, 20 °C, 1 % *i*PrOH in heptane, 1.0 ml/min					
Peak #	t_R / min	Area	Height	Area / %	Compound
1	10.864	6447388	489555	97.147	*endo*-**3e**
2	13.848	189320	10928	2.853	*endo*-**3e**
Total		6636708	500482	100.000	

10.5.3 Isolation at 100 µmol scale and 50 mM concentration with 5 mol% catalyst loading (Section 4.6.2)

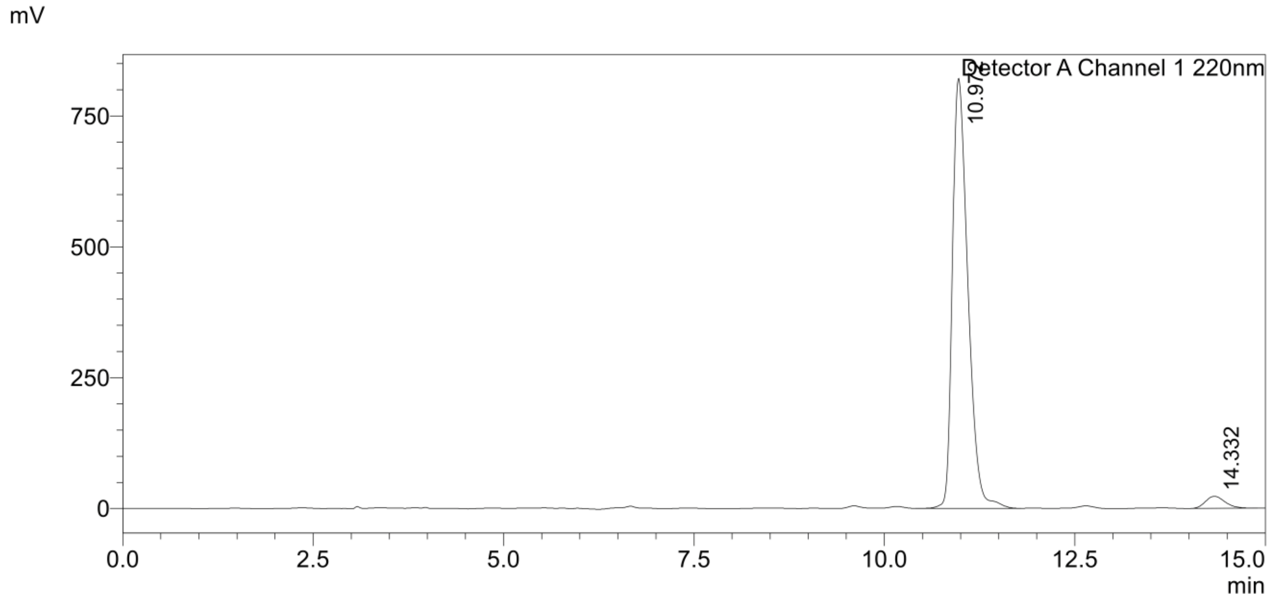

YMC CHIRAL ART Amylose-SA S-5 µm, 220 nm, 25 °C, 1 % *i*PrOH in heptane, 1.0 ml/min					
Peak #	t_R / min	Area	Height	Area / %	Compound
1	10.967	11433301	817051	96.605	*endo*-**3e**
2	14.317	401742	22660	3.395	*endo*-**3e**
Total		11835042	839711	100.000	

10.6 5-Nitro-6-(2-methoxyphenyl)bicyclo[2.2.1]hept-2-ene (3f)

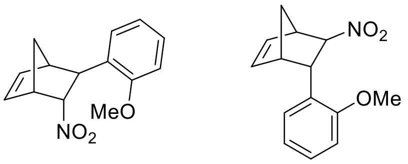

endo-**3f** *exo*-**3f**

For clarity, only one enantiomer is shown for both diastereomers.

10.6.1 Racemate

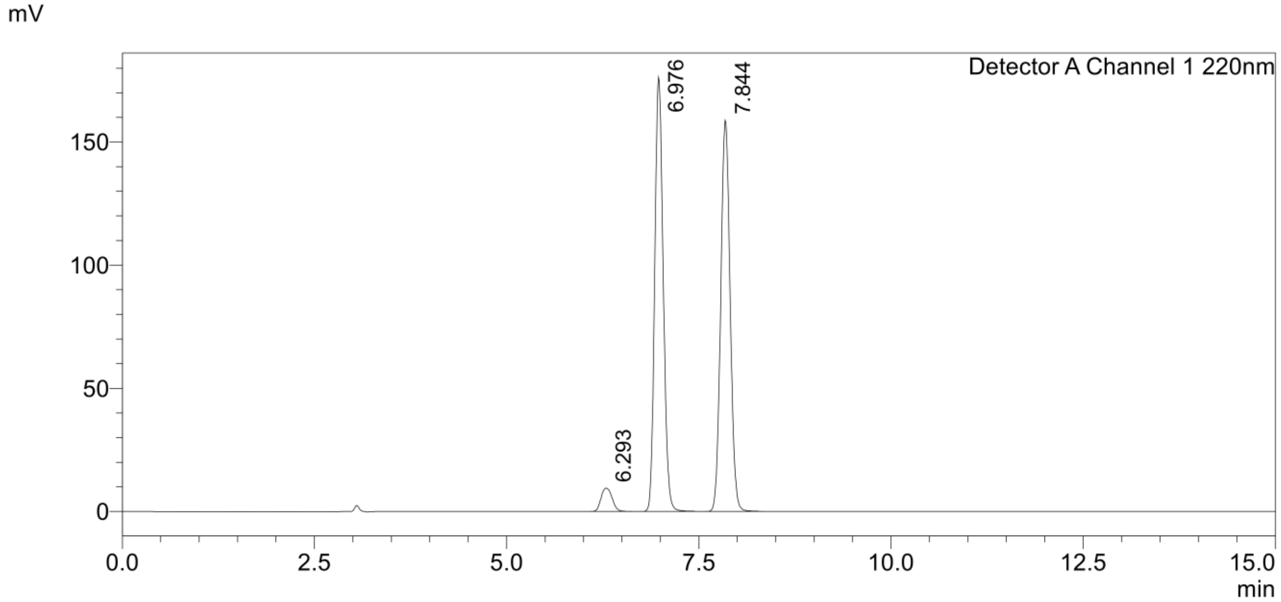

YMC CHIRAL ART Amylose-SA S-5 µm, 220 nm, 25 °C, 1 % *i*PrOH in heptane, 1.0 ml/min					
Peak #	t_R / min	Area	Height	Area / %	Compound
1	6.293	95613	9497	3.386	*exo*-**3f**
2	6.976	1363746	176228	48.298	*endo*-**3f**
3	7.844	1364272	158736	48.316	*endo*-**3f**
Total		2823631	344461	100.000	

10.6.2 Isolation at 50 µmol scale and 50 mM concentration with 10 mol% catalyst loading (Section 4.7.1)

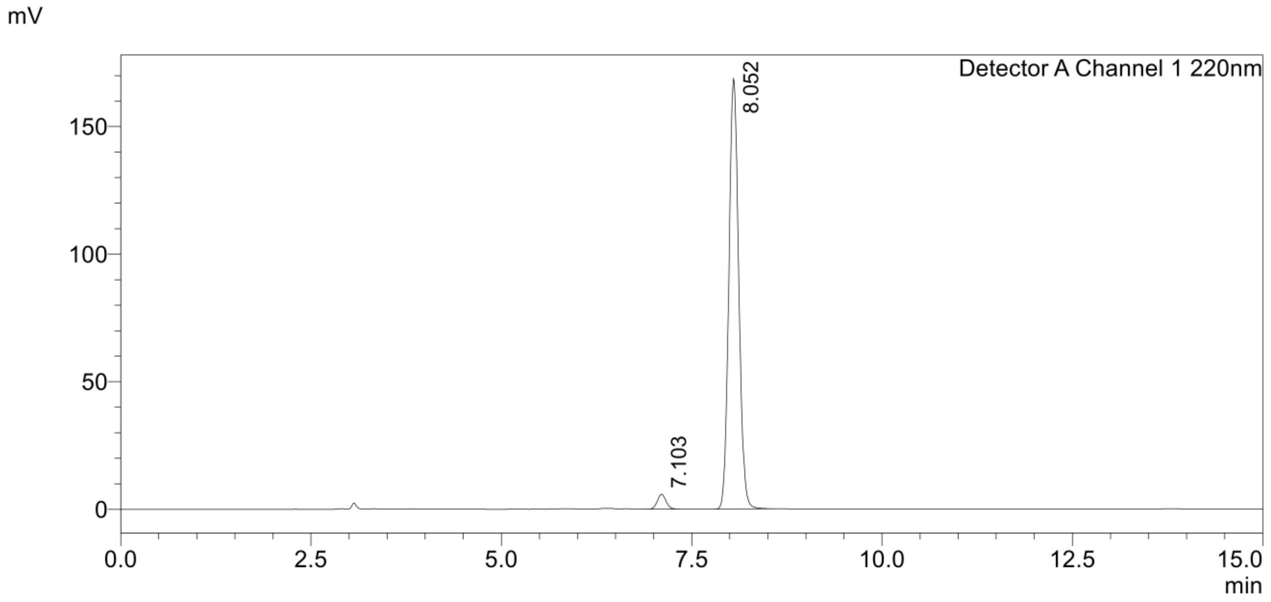

YMC CHIRAL ART Amylose-SA S-5 µm, 220 nm, 20 °C, 1 % *i*PrOH in heptane, 1.0 ml/min					
Peak #	t_R / min	Area	Height	Area / %	Compound
1	7.103	46416	5797	2.961	*endo*-**3f**
2	8.052	1521209	168586	97.039	*endo*-**3f**
Total		1567625	174384	100.000	

10.6.3 Isolation at 100 µmol scale and 50 mM concentration with 5 mol% catalyst loading (Section 4.7.2)

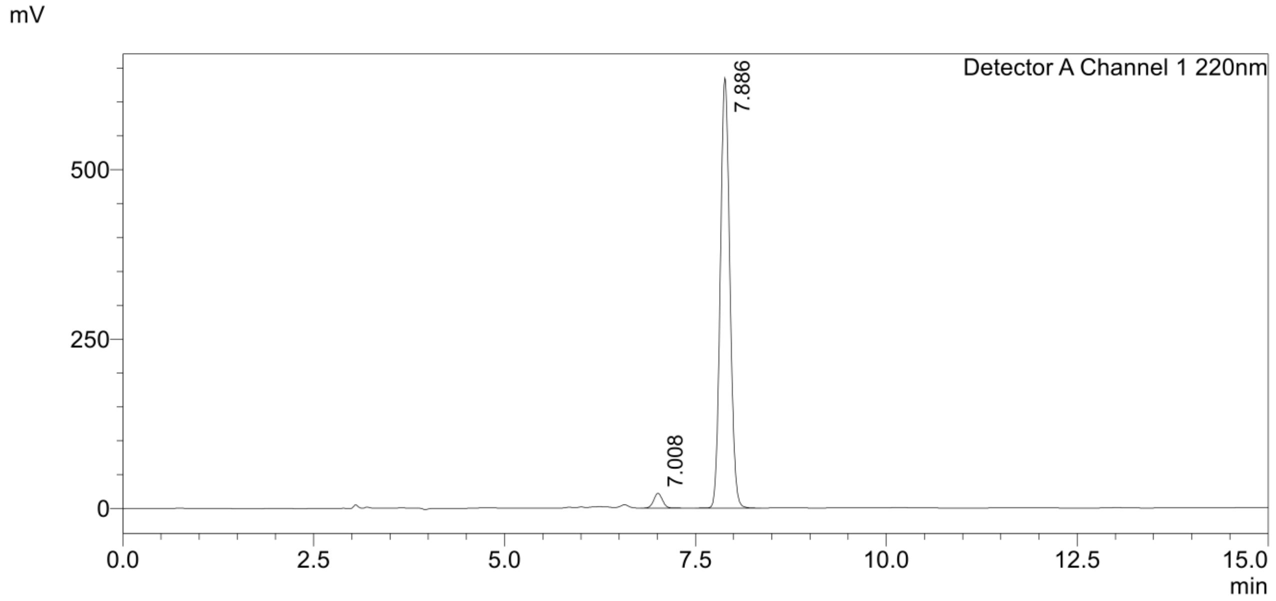

YMC CHIRAL ART Amylose-SA S-5 µm, 220 nm, 25 °C, 1 % *i*PrOH in heptane, 1.0 ml/min					
Peak #	t_R / min	Area	Height	Area / %	Compound
1	7.008	173908	21780	3.021	*endo*-**3f**
2	7.886	5581893	634516	96.979	*endo*-**3f**
Total		5755801	656295	100.000	

10.7 5-Nitro-6-(2,4-dimethoxyphenyl)bicyclo[2.2.1]hept-2-ene (3g)

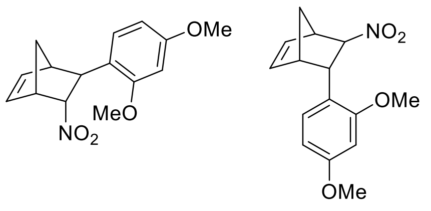

endo-**3g** *exo*-**3g**

For clarity, only one enantiomer is shown for both diastereomers.

10.7.1 Racemate

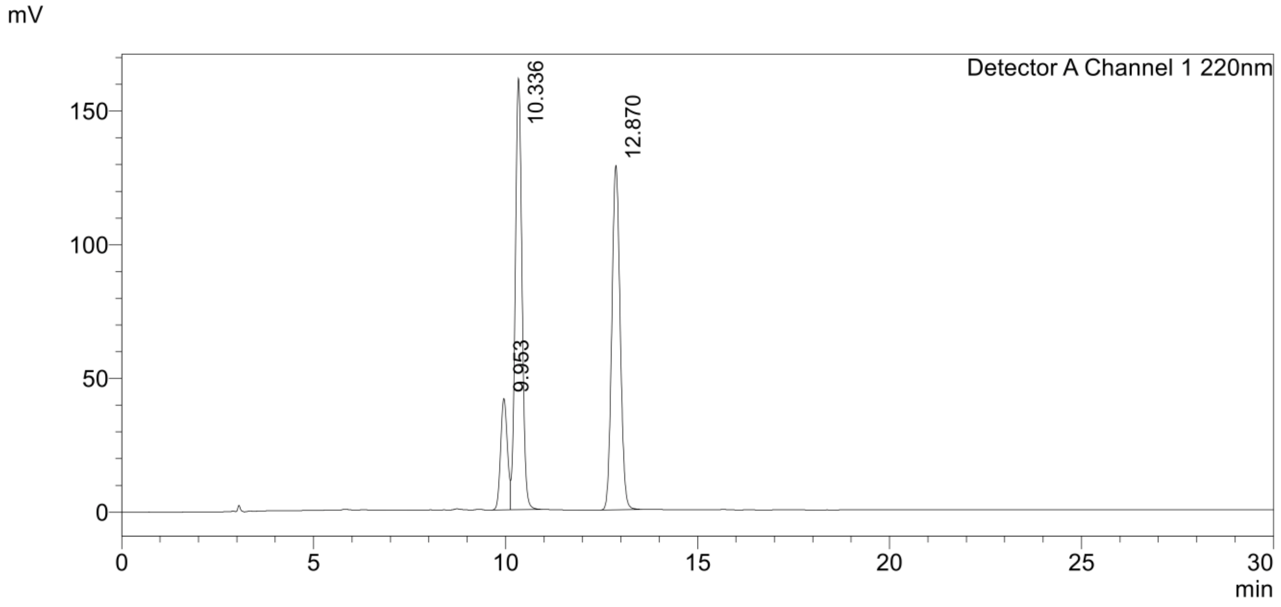

YMC CHIRAL ART Amylose-SA S-5 µm, 220 nm, 25 °C, 1 % *i*PrOH in heptane, 1.0 ml/min					
Peak #	t_R / min	Area	Height	Area / %	Compound
1	9.953	516686	41672	11.632	*exo*-**3g**
2	10.336	1972581	161307	44.408	*endo*-**3g**
3	12.870	1952674	128773	43.960	*endo*-**3g**
Total		4441941	331752	100.000	

10.7.2 Isolation at 50 µmol scale and 50 mM concentration with 10 mol% catalyst loading (Section 4.8.1)

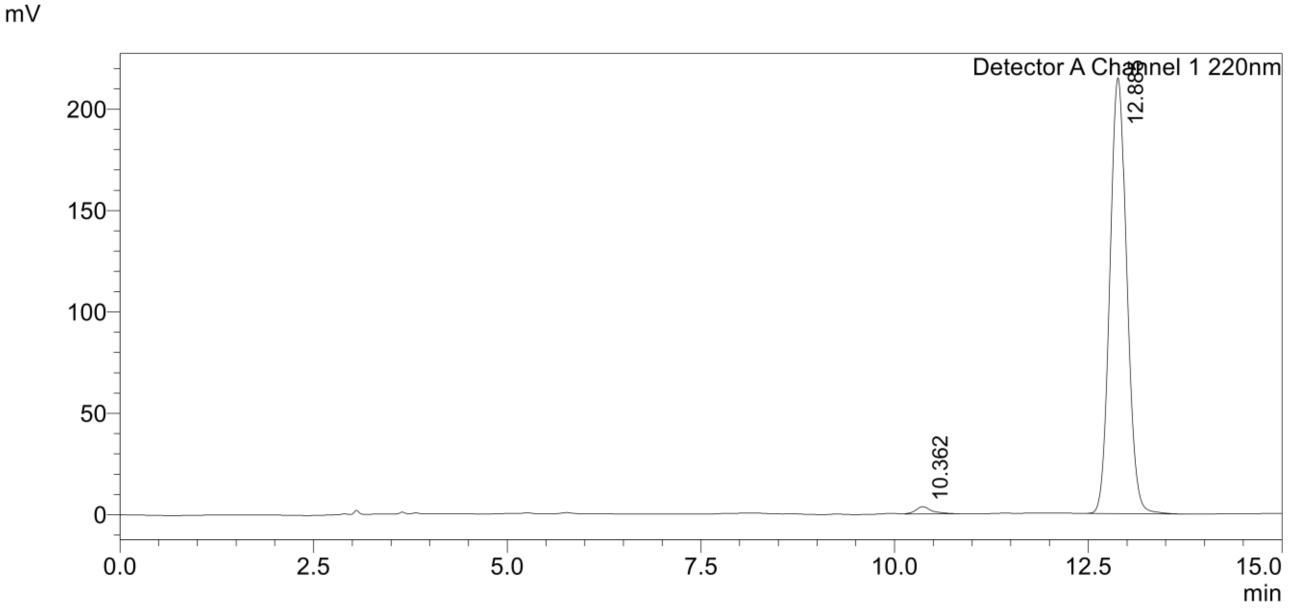

YMC CHIRAL ART Amylose-SA S-5 µm, 220 nm, 25 °C, 1 % *i*PrOH in heptane, 1.0 ml/min					
Peak #	t_R / min	Area	Height	Area / %	Compound
1	10.362	51307	3566	1.549	*endo*-**3g**
2	12.885	3261080	214835	98.451	*endo*-**3g**
Total		3312387	218401	100.000	

10.7.3 Isolation at 100 µmol scale and 50 mM concentration with 5 mol% catalyst loading (Section 4.8.2)

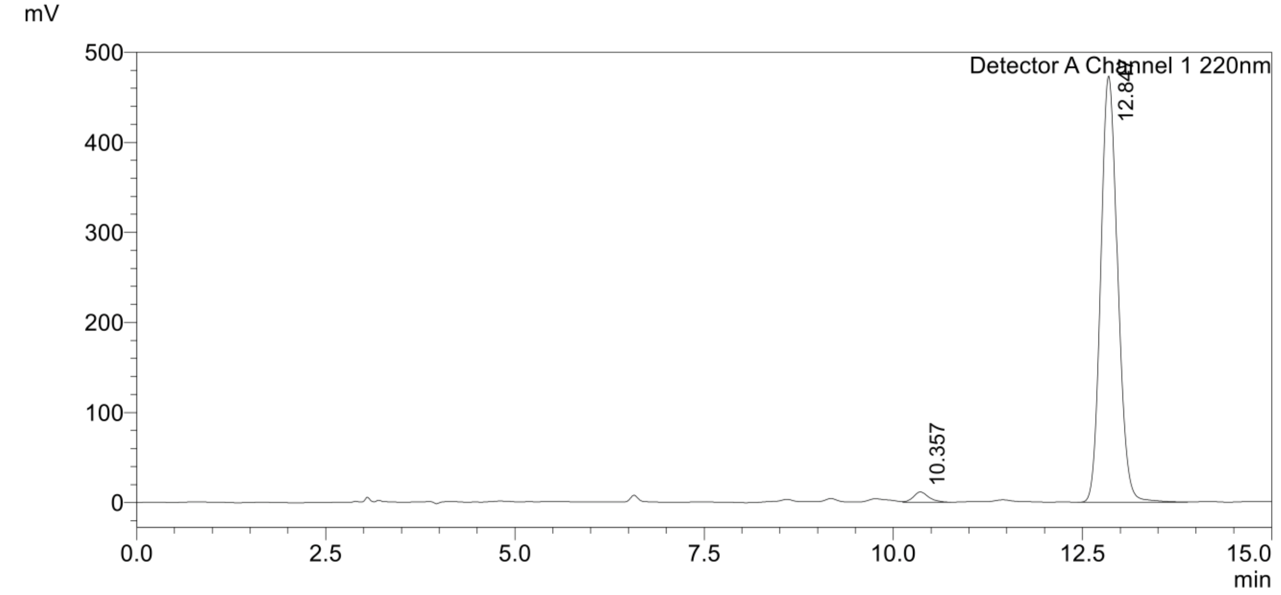

YMC CHIRAL ART Amylose-SA S-5 µm, 220 nm, 25 °C, 1 % *i*PrOH in heptane, 1.0 ml/min					
Peak #	t_R / min	Area	Height	Area / %	Compound
1	10.357	158825	11372	2.112	*endo*-**3g**
2	12.847	7360499	473078	97.888	*endo*-**3g**
Total		7519324	484451	100.000	

10.8 5-Nitro-6-(3,4-dimethoxyphenyl)bicyclo[2.2.1]hept-2-ene (3h)

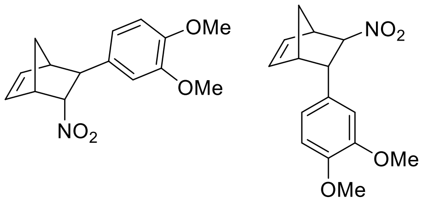

endo-**3h** *exo*-**3h**

For clarity, only one enantiomer is shown for both diastereomers.

10.8.1 Racemate

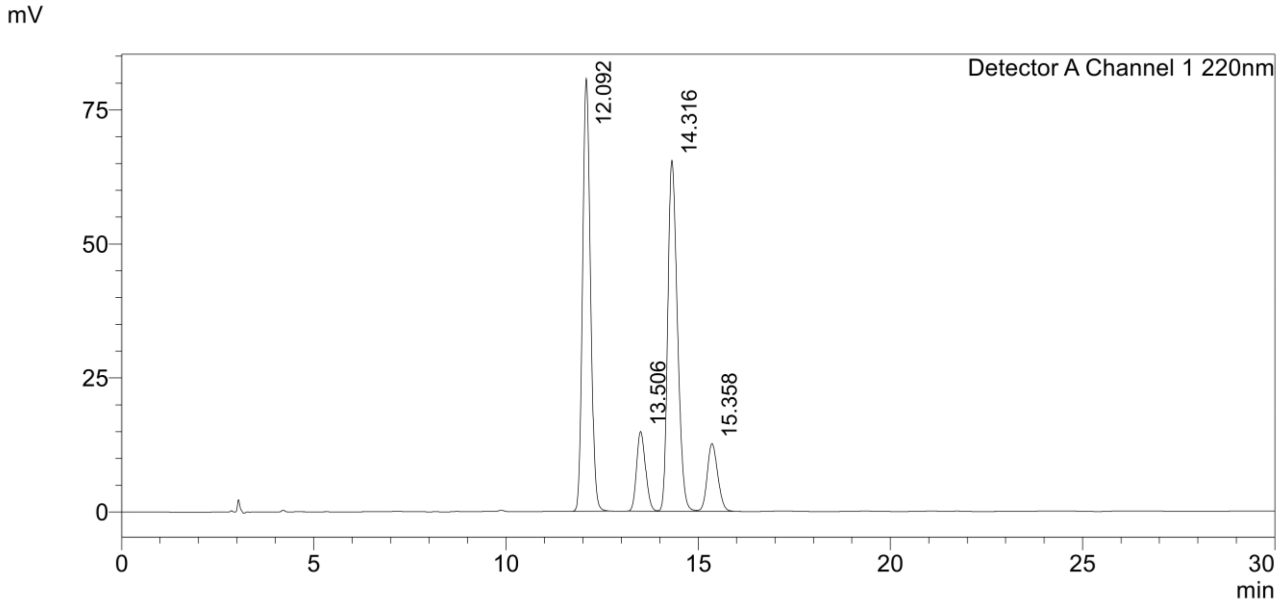

YMC CHIRAL ART Amylose-SA S-5 µm, 220 nm, 25 °C, 3 % *i*PrOH in heptane, 1.0 ml/min					
Peak #	t_R / min	Area	Height	Area / %	Compound
1	12.092	1149271	80689	41.346	*endo*-**3h**
2	13.506	238406	14852	8.577	*exo*-**3h**
3	14.316	1151432	65418	41.424	*endo*-**3h**
4	15.358	240523	12618	8.653	*exo*-**3h**
Total		2779632	173577	100.000	

10.8.2 Isolation at 50 µmol scale and 50 mM concentration with 10 mol% catalyst loading (Section 4.9.1)

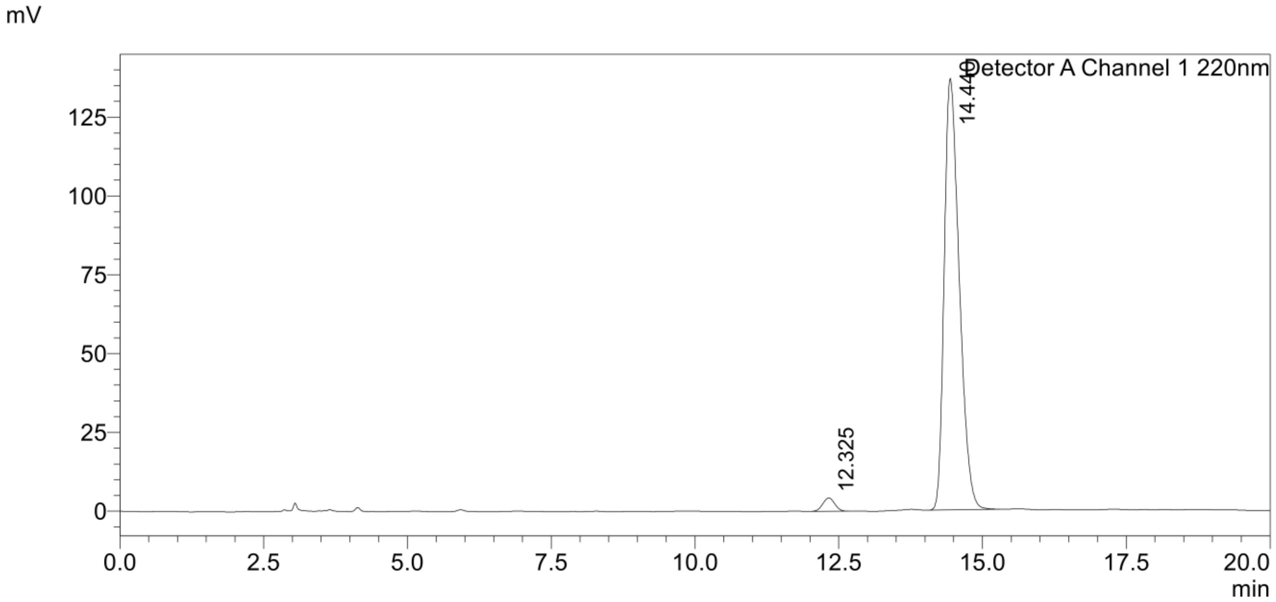

YMC CHIRAL ART Amylose-SA S-5 µm, 220 nm, 25 °C, 3 % *i*PrOH in heptane, 1.0 ml/min					
Peak #	t_R / min	Area	Height	Area / %	Compound
1	12.325	60788	4249	2.388	*endo*-**3h**
2	14.440	2485088	136825	97.612	*endo*-**3h**
Total		2545875	141074	100.000	

10.8.3 Isolation at 100 µmol scale and 50 mM concentration with 5 mol% catalyst loading (Section 4.9.2)

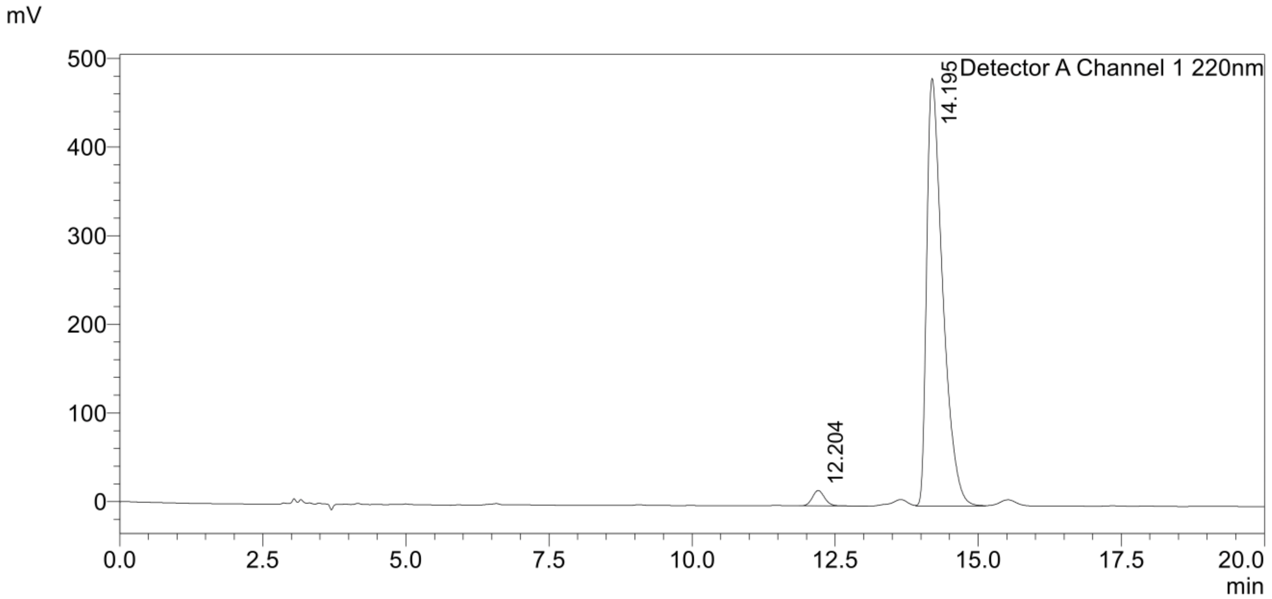

YMC CHIRAL ART Amylose-SA S-5 µm, 220 nm, 25 °C, 3 % *i*PrOH in heptane, 1.0 ml/min					
Peak #	t_R / min	Area	Height	Area / %	Compound
1	12.204	260435	17328	2.702	*endo*-**3h**
2	14.195	9376726	482533	97.298	*endo*-**3h**
Total		9637161	499861	100.000	

10.9 2-(3-Nitrobicyclo[2.2.1]hept-5-en-2-yl)thiophene (SI-25)

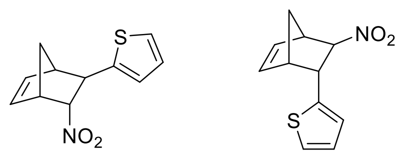

endo-**SI-25** *exo*-**SI-25**

For clarity, only one enantiomer is shown for both diastereomers.

10.9.1 Racemate

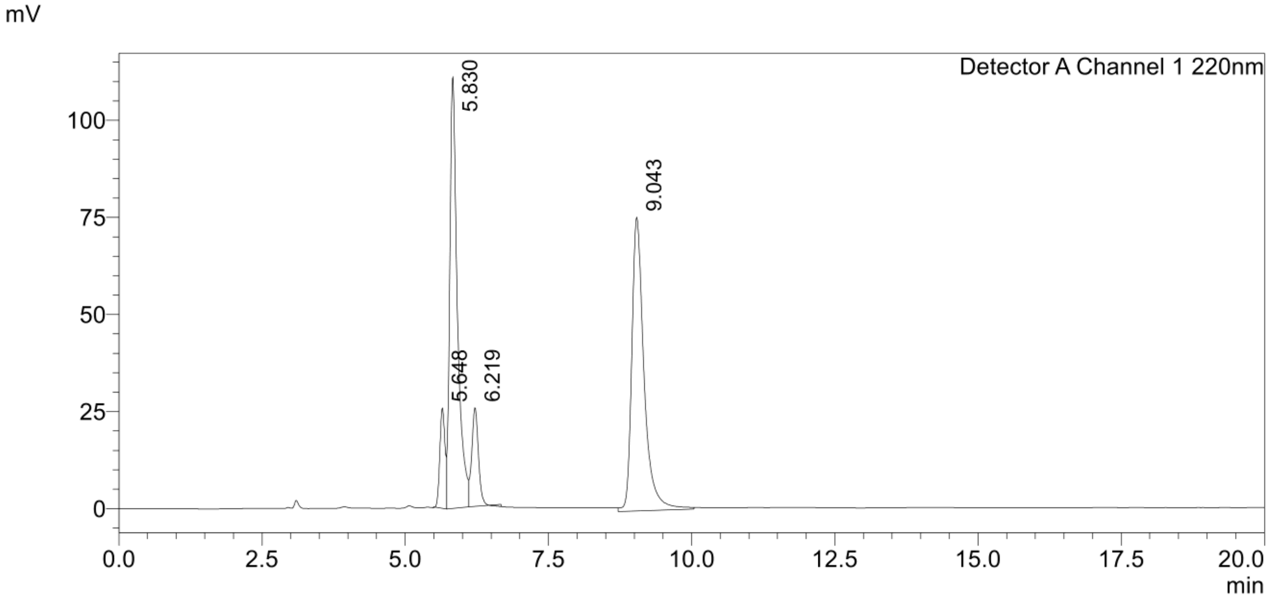

Dr. Maisch ReproSil Chiral-AM 5 µm, 220 nm, 25 °C, 3 % iPrOH in heptane, 1.0 ml/min					
Peak #	t_R / min	Area	Height	Area / %	Compound
1	5.648	170864	25788	6.768	*exo*-**SI-25**
2	5.830	1028974	111039	40.760	*endo*-**SI-25**
3	6.219	203954	25392	8.079	*exo*-**SI-25**
4	9.043	1120650	75561	44.392	*endo*-**SI-25**
Total		2524441	237780	100.000	

10.9.2 First experiment in analytical scale (12.5 µmol scale, 50 mM concentration with 10 mol% catalyst loading, no isolation; Section 4.11)

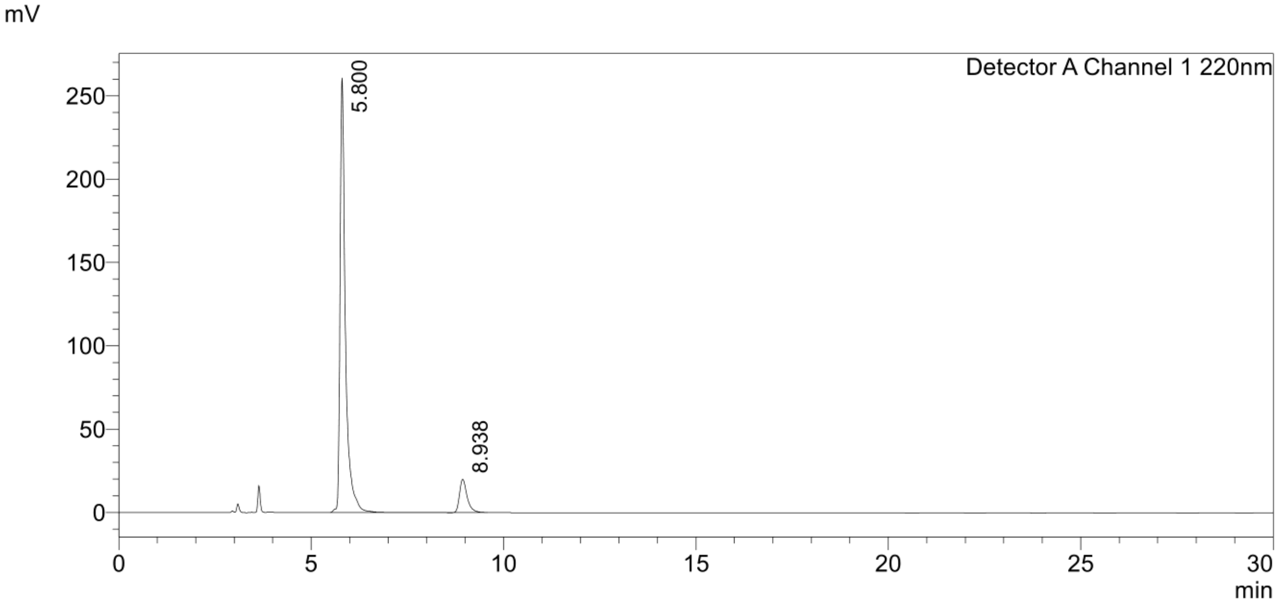

Dr. Maisch ReproSil Chiral-AM 5 µm, 220 nm, 25 °C, 3 % iPrOH in heptane, 1.0 ml/min					
Peak #	t_R / min	Area	Height	Area / %	Compound
1	5.800	2523109	260790	90.546	*endo*-**SI-25**
2	8.938	263450	19943	9.454	*endo*-**SI-25**
Total		2786560	280733	100.000	

10.9.3 Second experiment in analytical scale (12.5 µmol scale, 50 mM concentration with 10 mol% catalyst loading, no isolation; Section 4.11)

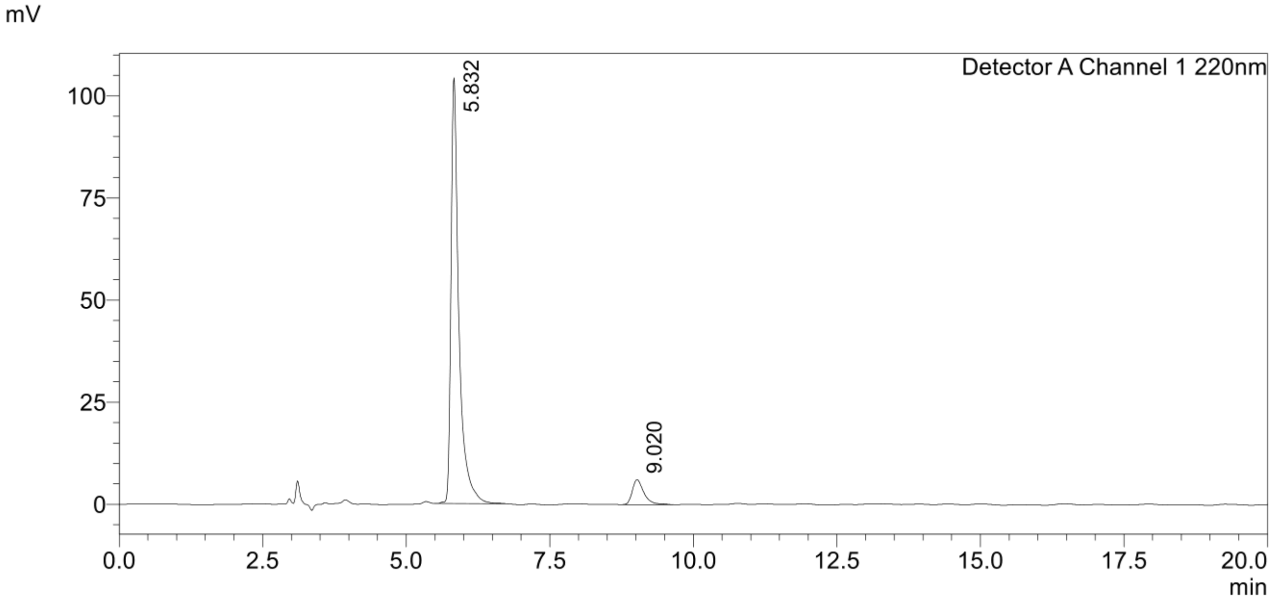

Dr. Maisch ReproSil Chiral-AM 5 µm, 220 nm, 25 °C, 3 % iPrOH in heptane, 1.0 ml/min					
Peak #	t_R / min	Area	Height	Area / %	Compound
1	5.832	994359	104253	92.035	*endo*-**SI-25**
2	9.020	86055	6125	7.965	*endo*-**SI-25**
Total		1080414	110377	100.000	

10.10 5-(2-chlorophenyl)-6-nitrobicyclo[2.2.1]hept-2-ene (SI-26)

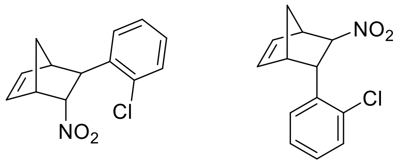

endo-**SI-26** *exo*-**SI-26**

For clarity, only one enantiomer is shown for both diastereomers.

10.10.1 Racemate

For this racemate two fractions were separated by column chromatography. F1 contained mostly minor diastereomer *exo*-**SI-26** and small amounts of major diastereomer *endo*-**SI-26**. F2 contained pure major diastereomer *endo*-**SI-26**:

Fraction F1:

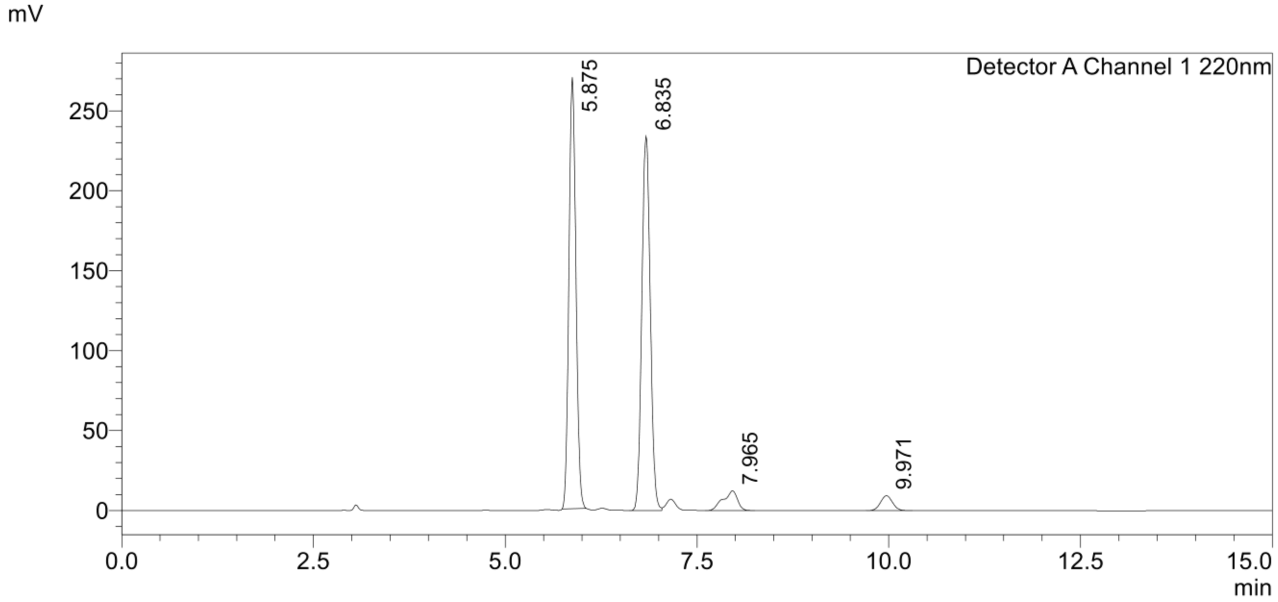

YMC CHIRAL ART Amylose-SA S-5 µm, 220 nm, 25 °C, 1 % *i*PrOH in heptane, 1.0 ml/min					
Peak #	t_R / min	Area	Height	Area / %	Compound
1	5.875	1721620	269745	46.007	*exo*-**SI-26**
2	6.835	1757356	233966	46.962	*exo*-**SI-26**
3	7.965	161376	12316	4.312	*endo*-**SI-26** (+ impurity)
4	9.971	101718	9261	2.718	*endo*-**SI-26**
Total		3742070	525289	100.000	

Fraction F2:

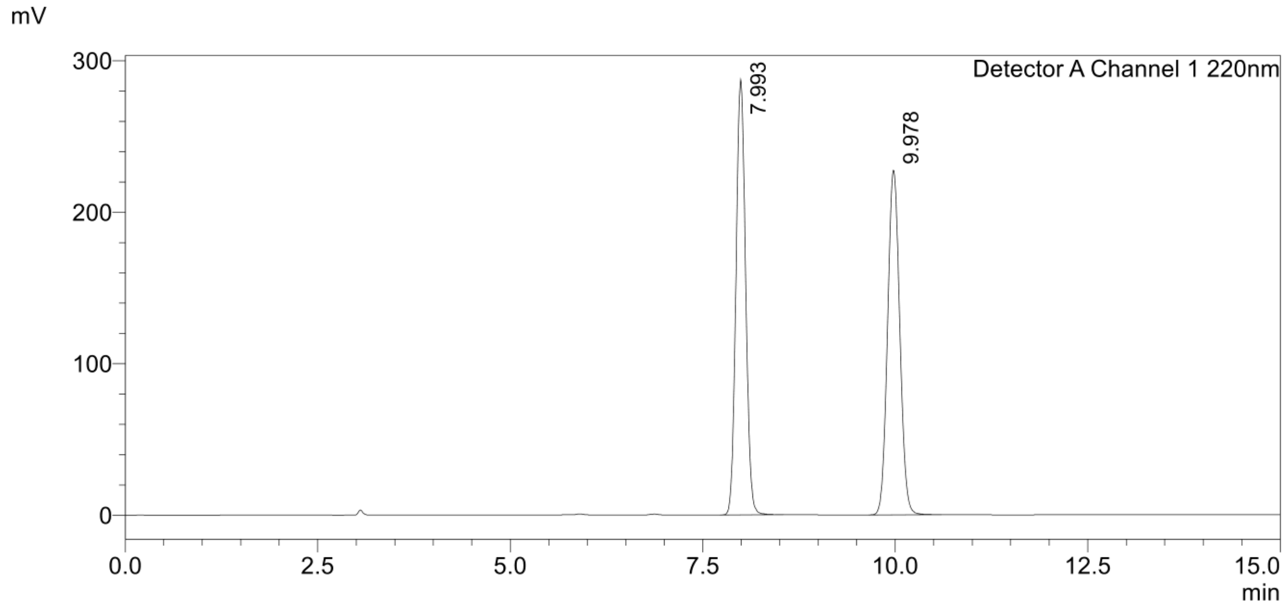

YMC CHIRAL ART Amylose-SA S-5 µm, 220 nm, 25 °C, 1 % *i*PrOH in heptane, 1.0 ml/min					
Peak #	t_R / min	Area	Height	Area / %	Compound
1	7.993	2508835	287343	49.937	*endo*-**SI-26**
2	9.978	2515138	227532	50.063	*endo*-**SI-26**
Total		5023973	514875	100.000	

10.10.2 First experiment in analytical scale (12.5 µmol scale, 50 mM concentration with 10 mol% catalyst loading, no isolation; Section 4.11)

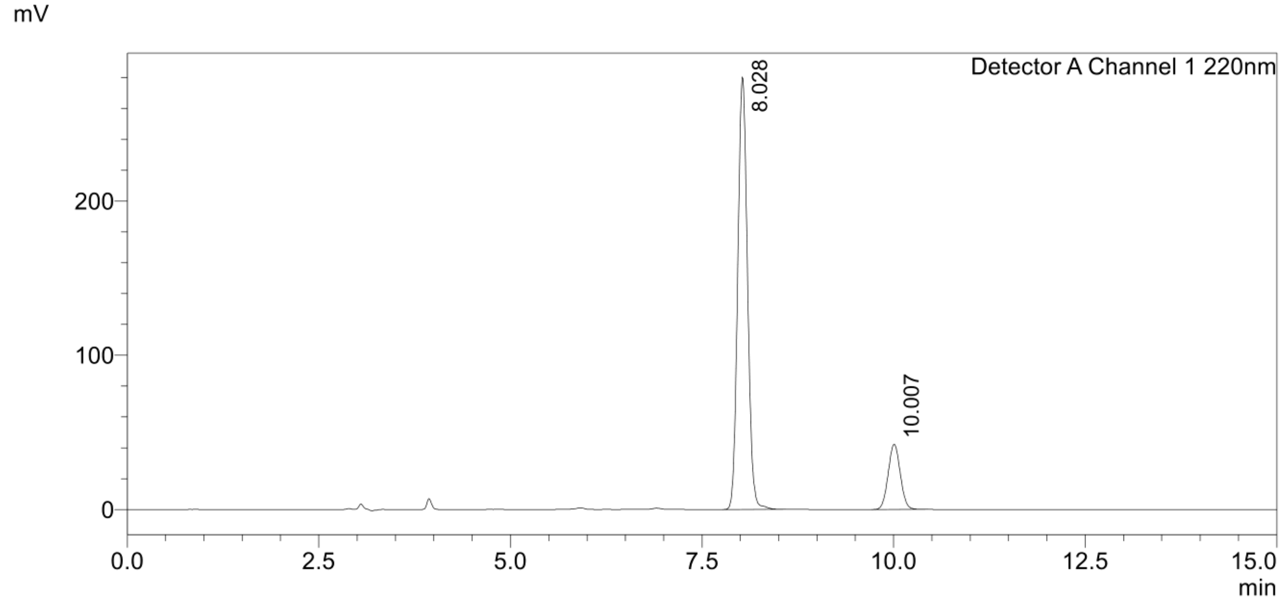

YMC CHIRAL ART Amylose-SA S-5 µm, 220 nm, 25 °C, 1 % *i*PrOH in heptane, 1.0 ml/min					
Peak #	t_R / min	Area	Height	Area / %	Compound
1	8.028	2480543	280178	84.141	*endo*-**SI-26**
2	10.007	467540	42264	15.859	*endo*-**SI-26**
Total		2948083	322441	100.000	

10.10.3 Second experiment in analytical scale (12.5 µmol scale, 50 mM concentration with 10 mol% catalyst loading, no isolation; Section 4.11)

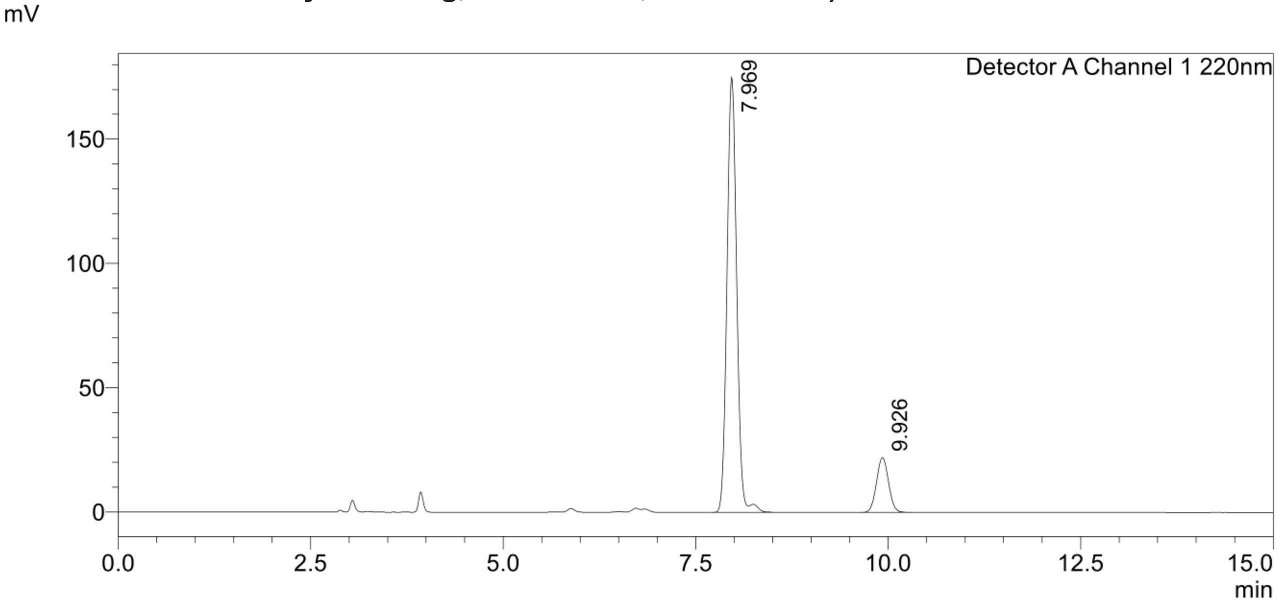

YMC CHIRAL ART Amylose-SA S-5 µm, 220 nm, 25 °C, 1 % *i*PrOH in heptane, 1.0 ml/min					
Peak #	t_R / min	Area	Height	Area / %	Compound
1	7.969	1530932	174913	86.394	*endo*-**SI-26**
2	9.926	241095	22077	13.606	*endo*-**SI-26**
Total		1772027	196991	100.000	

10.11 *N*-ethyl-3-phenylbicyclo[2.2.1]heptan-2-amine (12)

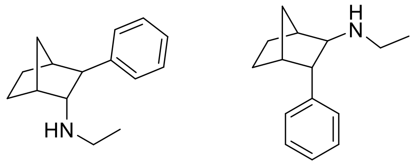

endo-**12** *exo*-**12**

For clarity, only one enantiomer is shown for both diastereomers. The shown configuration for *endo*-**12** was determined to be the major enantiomer of *endo*-**3a** (section 6), which was used to synthesize *endo*-**12**.

10.11.1 Racemate

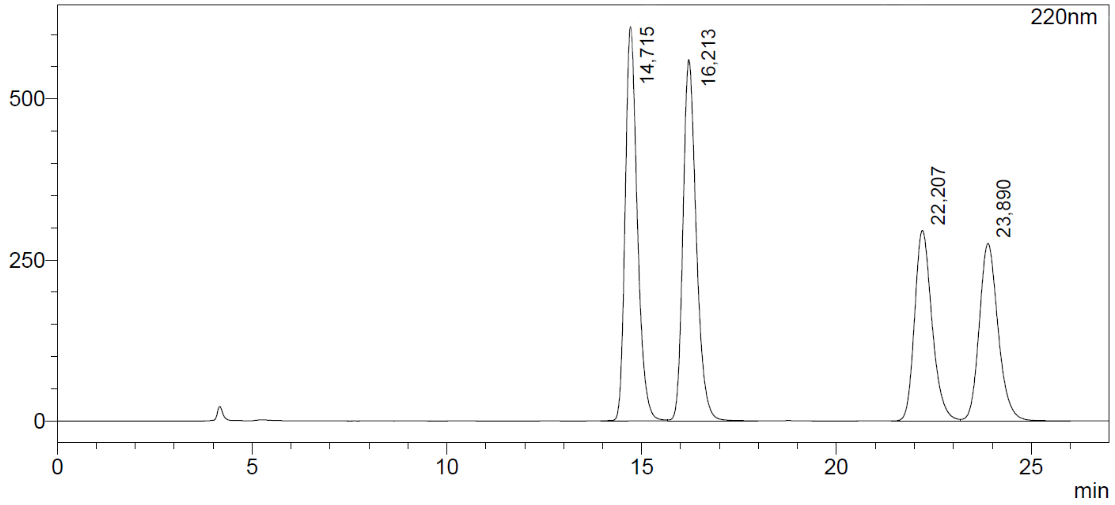

Daicel Chiralpak AD (10 µm), 220 nm, 25 °C, 10 % NH_4HCO_3 (20 mM aq.) in MeOH, 1.0 ml/min			
Peak #	t_R / min	Area / %	Compound
1	14.715	29.403	*endo*-**12**
2	16.213	29.582	*endo*-**12**
3	22.207	20.441	*exo*-**12**
4	23.890	20.574	*exo*-**12**
Total		100.000	

10.11.2 Synthesis of *endo*-12 from enantioenriched *endo*-3a (section 5)

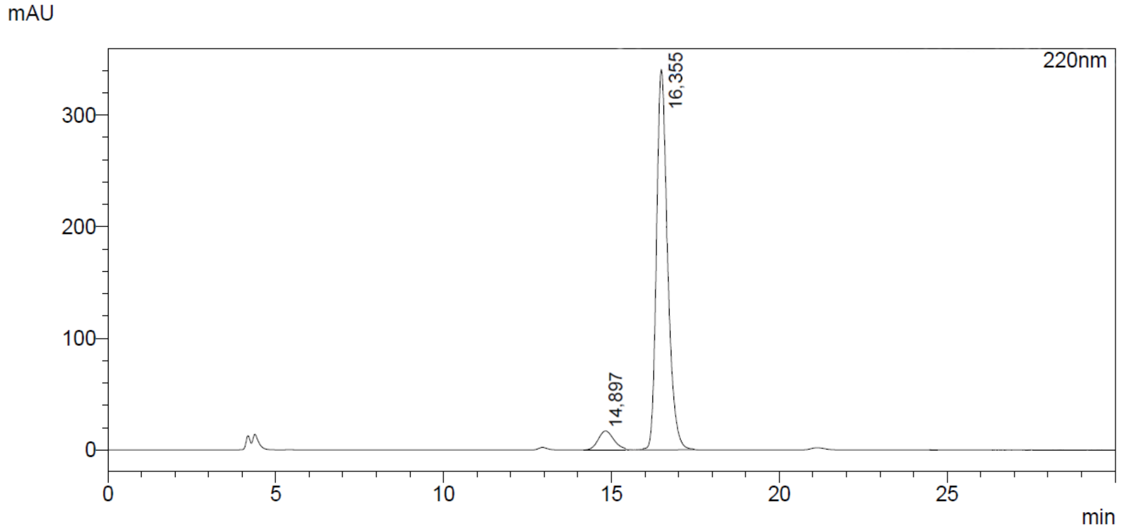

Daicel Chiralpak AD (10 µm), 220 nm, 25 °C, 10 % NH_4HCO_3 (20 mM aq.) in MeOH, 1.0 ml/min			
Peak #	t_R / min	Area / %	Compound
1	14.897	5.743	*endo*-**12**
2	16.355	94.257	*endo*-**12**
Total		100.000	

12. GC traces

12.1 *Endo*-5-cyclohexyl-6-nitrobicyclo[2.2.1]hept-2-ene (*endo*-3i)

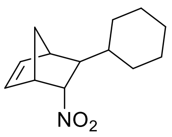

endo-**3i**

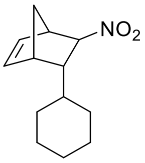

exo-**3i**

For clarity, only one enantiomer is shown for both diastereomers.

12.1.1 Racemate

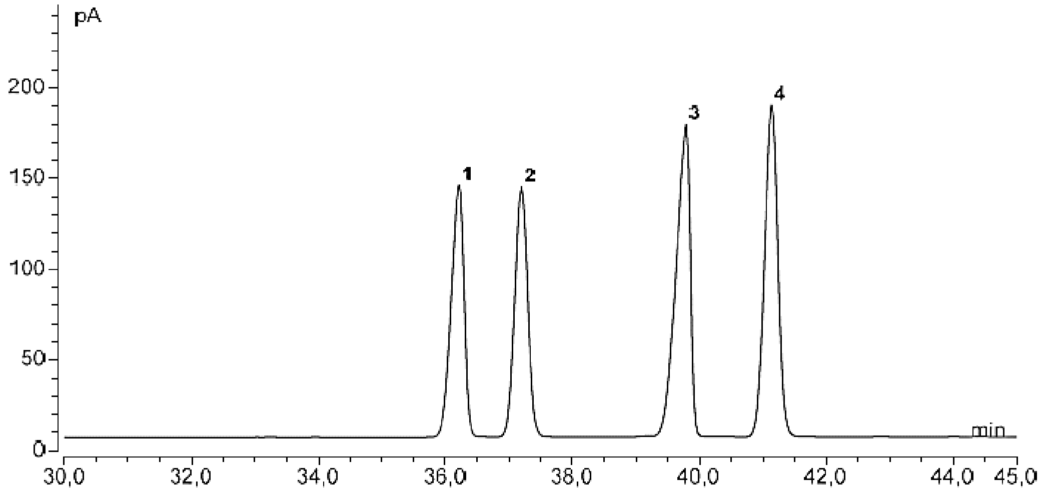

BGB-176 30m x 0.25mm, injection temperature: 220 °C, 80 °C, 0.5 bar He			
Peak #	t_R / min	Area / %	Compound
1	36.21	21.00	*exo*-**3i**
2	37.20	20.98	*exo*-**3i**
3	39.79	28.95	*endo*-**3i**
4	41.14	29.07	*endo*-**3i**
Total		100.00	

12.1.2 Isolation at 100 µmol scale and 50 mM concentration with 10 mol% catalyst loading (Section 4.10.1)

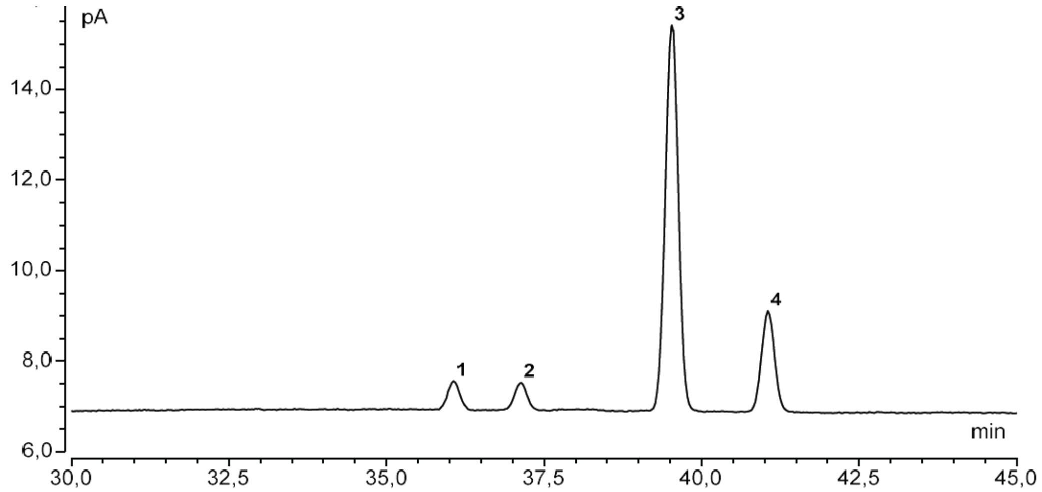

BGB-176 30m x 0.25mm, injection temperature: 220 °C, 80 °C, 0.5 bar He			
Peak #	t_R / min	Area / %	Compound
1	36.06	4.95	*exo*-**3i**
2	37.13	4.75	*exo*-**3i**
3	39.53	71.42	*endo*-**3i**
4	41.05	18.89	*endo*-**3i**
Total		100.00	
